# Eyeless razor clam *Sinonovacula constricta* discriminates light spectra through opsins to guide Ca^2+^ and cAMP signaling pathways

**DOI:** 10.1016/j.jbc.2023.105527

**Published:** 2023-12-01

**Authors:** Fei Kong, Zhaoshou Ran, Mengqi Zhang, Kai Liao, Deshui Chen, Xiaojun Yan, Jilin Xu

**Affiliations:** 1 Key Laboratory of Aquacultural Biotechnology Ministry of Education, Ningbo University, Ningbo, Zhejiang, China; 2 Key Laboratory of Marine Biotechnology of Zhejiang Province, Ningbo University, Ningbo, Zhejiang, China; 3 Fujian Dalai Seedling Technology Co, LTD, Luoyuan, Fujian, China

**Keywords:** phototransduction, rhodopsin, photoreceptor, G protein-coupled receptor, invertebrate, nonvisual photosensitivity, marine bivalves, marine photoecology

## Abstract

Phototransduction is based on opsins that drive distinct types of Gα cascades. Although nonvisual photosensitivity has long been known in marine bivalves, the underlying molecular basis and phototransduction mechanism are poorly understood. Here, we introduced the eyeless razor clam *Sinonovacula constricta* as a model to clarify this issue. First, we showed that *S. constricta* was highly diverse in opsin family members, with a significant expansion in xenopsins. Second, the expression of putative *S. constricta* opsins was highly temporal-spatio specific, indicating their potential roles in *S. constricta* development and its peripheral photosensitivity. Third, by cloning four *S. constricta* opsins with relatively higher expression (*Sc_opsin1*, *5*, *7*, and *12*), we found that they exhibited different expression levels in response to different light environments. Moreover, we demonstrated that these opsins (excluding Sc_opsin7) couple with Gαq and Gαi cascades to mediate the light-dependent Ca^2+^ (Sc_opsin1 and 5) and cAMP (Sc_opsin12) signaling pathways. The results indicated that Sc_opsin1 and 5 belonged to Gq-opsins, Sc_opsin12 belonged to Gi-opsins, while Sc_opsin7 might act as a photo-isomerase. Furthermore, we found that the phototransduction function of *S. constricta* Gq-opsins was dependent on the lysine at the seventh transmembrane domain, and greatly influenced by the external light spectra in a complementary way. Thus, a synergistic photosensitive system mediated by opsins might exist in *S. constricta* to rapidly respond to the transient or subtle changes of the external light environment. Collectively, our findings provide valuable insights into the evolution of opsins in marine bivalves and their potential functions in nonvisual photosensitivity.

Light is a crucial environmental factor that impacts both terrestrial and aquatic ecosystems on Earth. Consequently, light perception plays a critical role in the development and survival of the entire animal kingdom. For example, visual photosensitivity enables animals to identify mates, predators, and preys, while nonvisual photosensitivity allows them to regulate circadian rhythms, physiological metabolism, and even behaviors (1, 2). However, due to the rapid development of modern society, light pollution has emerged as a significant concern. To effectively harness light, it is imperative to comprehend how animal sense and respond to it.

Opsins are the primary molecules that regulate light sensitivity and utilization in animals, enabling them to perform various biological functions (3). Specifically, opsins belong to the G protein-coupled receptors (GPCRs) superfamily and possess seven transmembrane (TM) helixes, characterized by a lysine (K) residue at the seventh helix (3). Through this K residue, opsins form functional visual pigments (photopigments) when covalently bound to a vitamin A-derived chromophore, such as 11-cis-retinal, *via* a Schiff base (3, 4, 5, 6). When photopigments absorb light (photons), the chromophore undergoes isomerization from a cis to an all-trans state, resulting in a conformational change in the opsin. This conformational change allows the opsin to bind to a corresponding specific heterotrimeric G protein, leading to the dissociation of the G protein. Subsequently, the alpha subunit of the G protein (Gα protein) dissociates and interacts with downstream second messenger systems, triggering the depolarization or hyperpolarization of the cell membrane potential (7, 8, 9, 10).

With the increasing availability of animal genomes, research on the classification and evolution of the opsin family has reached deeper levels than ever before. To date, the opsin family can generally be divided into five major lineages: the rhabdomeric (r-) opsins (also known as Gq-opsins, which are Gq-protein-coupled opsins), the ciliary (c-) opsins, the Cnidops, the Group 4 opsins [including the Go-opsin, retinal G protein-coupled receptor opsin (RGR opsin), peropsin, retinochrome, and neuropsin], and the xenopsins (4, 5, 11). However, compared to vertebrates and arthropods (12, 13), the evolution and function of opsins in marine bivalves are still poorly understood. Besides, previous studies have primarily focused on species with well-developed eyes, such as cephalopods (14, 15), gastropods (16), and scallops (17, 18, 19, 20, 21). In contrast, the photosensitivity and underlying molecular basis of eyeless marine bivalves have been largely overlooked (22), including their potential phototransduction pathways.

The razor clam *Sinonovacula constricta* (Lamarck 1818) is a typical eyeless marine bivalve species found along the western Pacific coast (23). It holds significant economic and nutritional value (23). Despite lacking eyes and adopting a burrowing lifestyle after metamorphosis, *S. constricta* demonstrates robust photosensitivity throughout its life, as established by our lab and other researchers. Specifically, *S. constricta* larvae exhibit positive responses to light during their pelagic phases (24). Optimal light intensity benefits *S. constricta* by shortening spawning time, increasing spawning capacity, enhancing the survival rate of planktonic larvae, and promoting juvenile growth (25). Interestingly, yellow light significantly stimulates the growth, digestion ability, and antioxidant capability of juvenile *S. constricta* (26). In adult *S. constricta*, a rhythmic fluctuation of melatonin exists, entrained by photoperiod (27). Additionally, adult *S. constricta* possesses a light-entrained circadian clock (28). Hence, *S. constricta* has potential as a model organism for studying the nonvisual photosensitivity in marine bivalves.

In the present study, we characterized the complete repertoire of the opsin family in *S. constricta* using its genomic data (23). Additionally, we investigated the Gα protein family in this bivalve, as it plays a crucial role in coupling with specific opsins to activate their respective phototransduction pathways. To gain insight into opsin differentiation in marine bivalves, we further identified the opsin family in four other typical bivalves from their genomes. Those include three eyeless species (*Mercenaria mercenaria*, *Modiolus philippinarum*, and *Crassostrea gigas*), as well as one species featuring numerous non-cephalic eyes (*Mizuhopecten yessoensis*). Subsequently, we analyzed the temporal and spatial expression patterns of *S. constricta* opsins based on transcriptomic data (23). We also cloned four representative *S. constricta* opsins with relatively higher expression and studied their expression patterns in response to different light spectra. Furthermore, we investigated the phototransduction pathways mediated by the cloned *S. constricta* opsins and determined their spectral sensitivity. These results are valuable for understanding the evolution of opsins in marine bivalves and illuminating the nonvisual photosensitivity and light requirements of these organisms. Ultimately, this research could facilitate the flexible application of light in breeding and farming practices for marine bivalves.

## Results

### Characteristics of putative *S. constricta* opsins and Gα proteins

**Figure 1:**
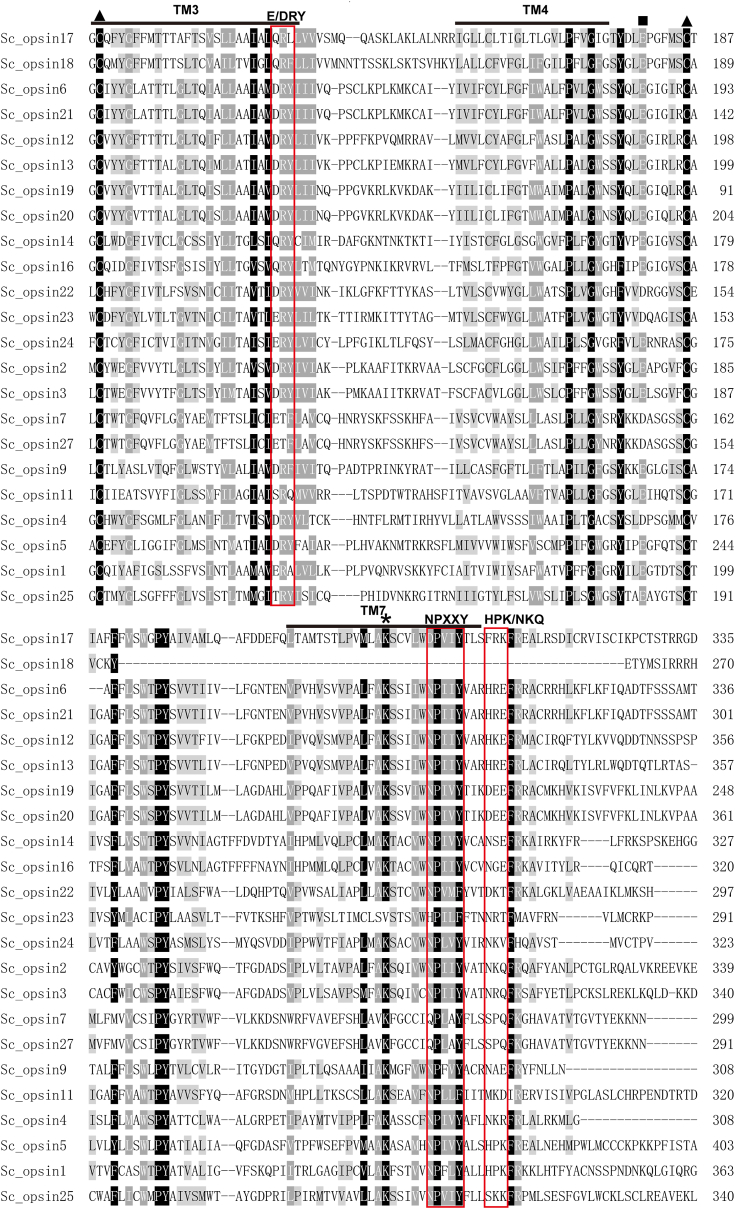
**Partial amino acid (aa) alignment of 23 putative *S. constricta* opsins.** The opsins were named based on their order of identification. Sequence alignment was conducted using Clustalx 2.1 software. Transmembrane domains (TM3, 4, and 7) were highlighted with *solid lines*. The lysine (K) residue covalently bound to the chromophore was marked with an *asterisk* (∗), the counterion site necessary for stabilizing the protonated Schiff base was denoted by a *square* (▪), and the two conserved cysteine (C) residues responsible for forming a disulfide bond were represented by *triangles* (▲). Conserved domains E/DRY, NPXXY, HPK/NKQ were indicated with *red frames*, respectively.

Initially, a total of 28 *opsin-like* sequences (named *Sc_opsin1*-*28*) were screened from the *S. constricta* genome. Following the removal of sequences with a similarity of over 98% and those lacking 7 TM helixes, a final set of 23 opsin homologs were selected as the targeted genes (Tables S1 and S2). Through amino acids (aa) sequence alignment, these homologs were found to encompass most of the conserved aa and domains characteristic of typical opsins (Fig. 1). These features included a K residue in the TM7 domain for chromophore binding, two cysteines (C) for disulfide bond formation, a counterion site with a negatively charged aa (glutamic acid, E) for stabilizing the protonated Schiff base (9, 29), the NPXXY motif for maintaining structural integrity and stability of the visual pigment (30), the typical HPK/NKQ pattern of r/c-opsins (31, 32, 33), and the E/DRY motif for stabilizing the inactive-state conformation of opsins (34). These results suggest that *S. constricta* opsins have undergone conserved evolution and likely possess potential phototransduction functions.

**Figure 2:**
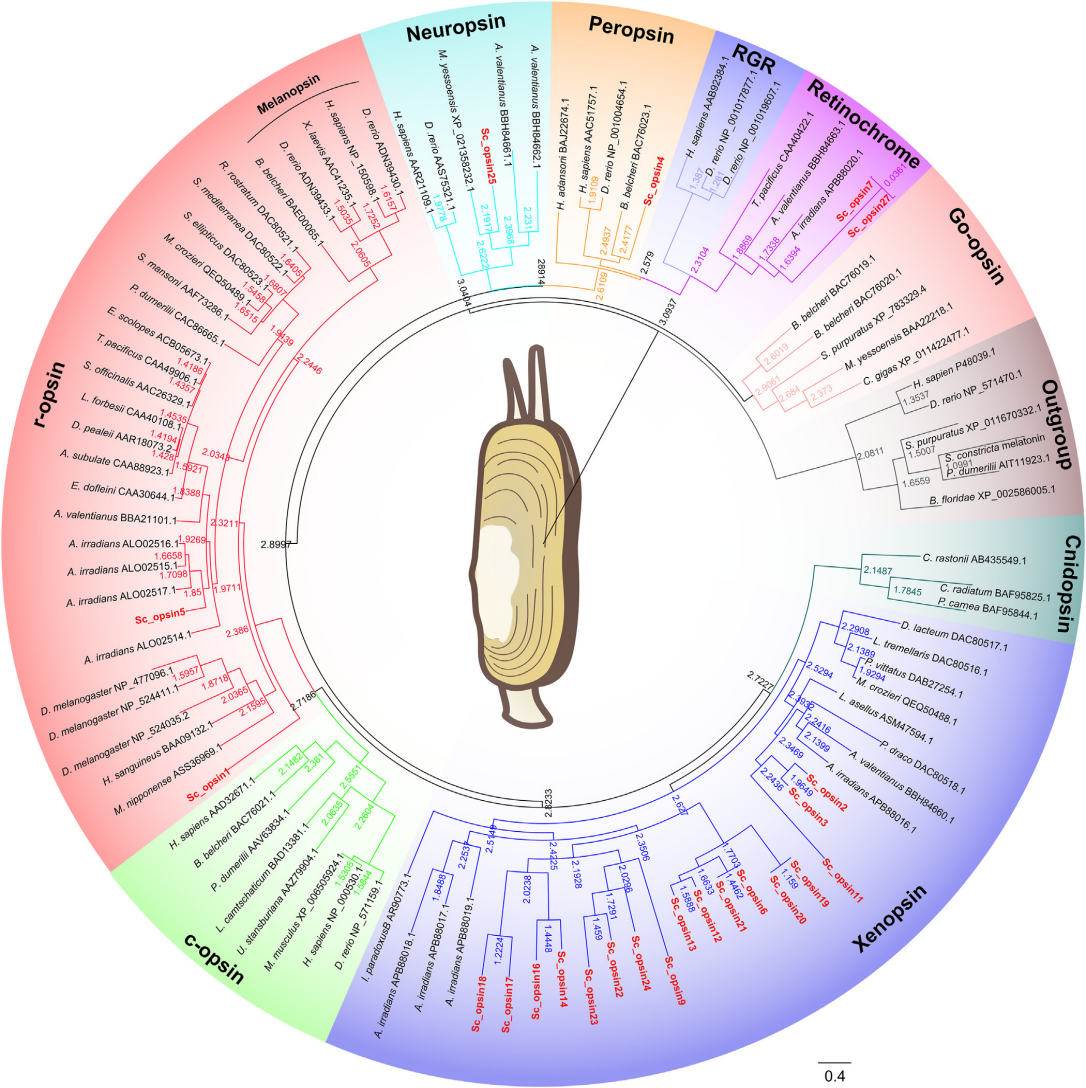
**Phylogenetic tree comparing deduced aa sequences of 23 putative *S. constricta* opsins with well-classified opsins from representative metazoan.** The tree was constructed using the maximum-likelihood method *via* SeaView software (PhyML algorithm) and visualized using Figtree v1.4.3 and Adobe Photoshop CS (version 6.0). Branch lengths of the tree topology were determined by minimizing the sum of squared differences between evolutionary and patristic distances. The 23 putative *S. constricta* opsins were highlighted in *red*. Different background colors denoted the various opsin subfamilies, as indicated in the figure, with the melatonin group selected as an outgroup to root the phylogenetic tree.

By constructing a phylogenetic tree, we categorized the opsin homologs from *S. constricta* into five distinct groups: two r-opsins (Sc_opsin1 and 5), one neuropsin (Sc_opsin25), one peropsin (Sc_opsin4), two retinochromes (Sc_opsin7 and 27), and seventeen xenopsins (Fig. 2). When compared to the opsin families of four other marine bivalves (Fig. S1 and Table S1), it was found that the xenopsin subfamily was significantly expanded within the genome of *S. constricta*. However, the Go-opsin, originally identified in the scallop *Patinopecten yessoensis* (18), was not present. Additionally, we identified a total of 19, 11, 15, and 16 opsin homologous sequences within the genomes of *M. mercenaria*, *M. philippinarum*, *C. gigas*, and *M. yessoensis*, respectively (Tables S1 and S3). This observation indicates a considerable divergence of the opsin family composition among marine bivalves (Fig. S1).

Meanwhile, a total of 6 Gα protein sequences were identified within the *S. constricta* genome: Gαq, Gαo, Gαi, Gαs1, Gαs2, and Gα12 (Table S2). The phylogenetic tree revealed the grouping of *S. constricta* Gα proteins with the corresponding Gα proteins of *Homo sapiens*, *Drosophila melanogaster*, *Platyneresis dumerilii*, and *Maritigrella crozieri* (Fig. S2*A*). Notably, the specificity of Gα proteins is primarily determined by their C-terminal aa sequence (35, 36). Consequently, we aligned the *S. constricta* Gα protein sequences with those of *H. sapiens* (Fig. S2*B*). The results demonstrated a pronounced conservation of the C-terminal aa sequence within the *S. constricta* Gα proteins, indicating their conserved functions throughout evolution.

### Temporal and spatial expression patterns of putative *S. constricta opsins*

**Figure 3:**
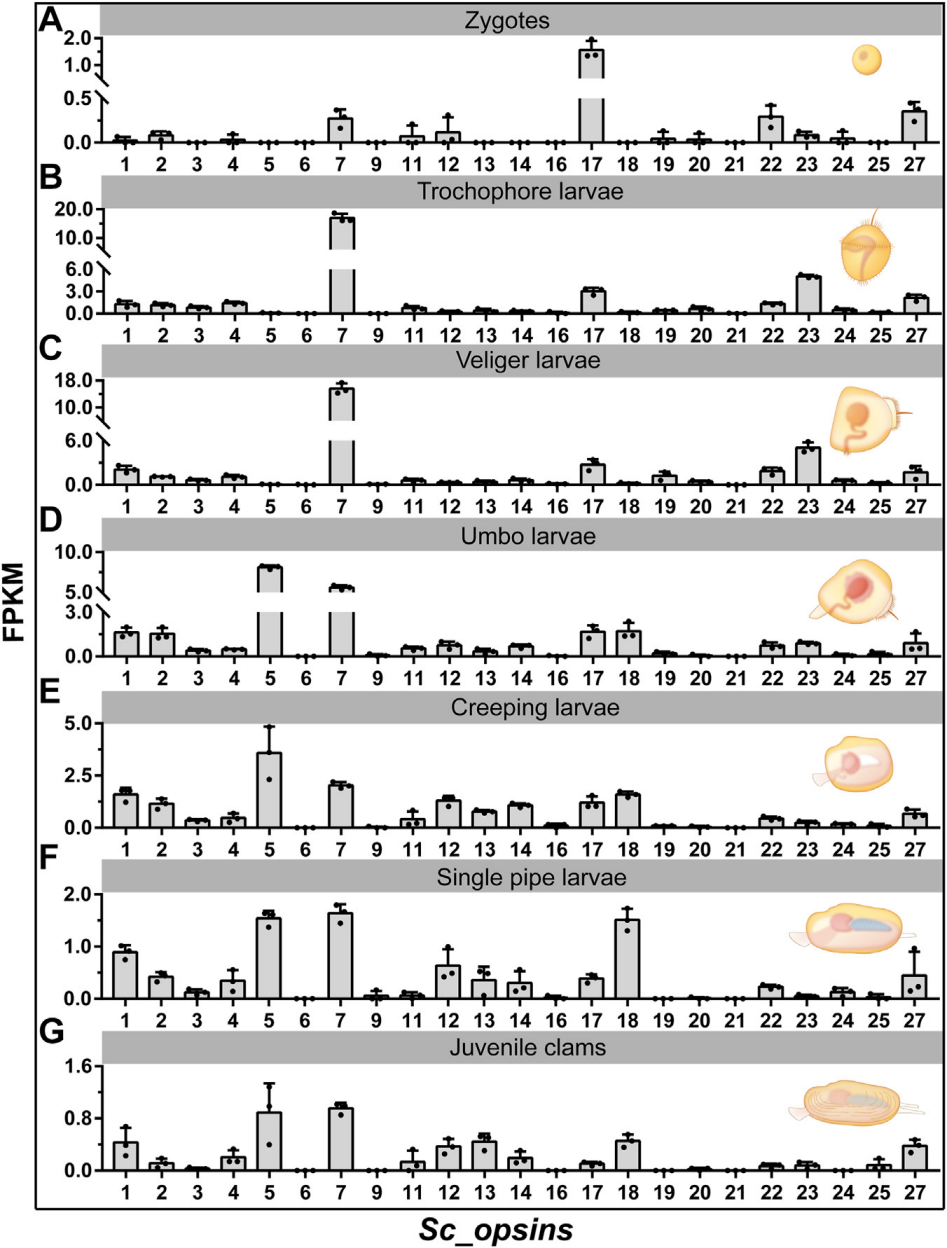
**Expression patterns of 23 putative *S. constricta opsins* at various developmental stages based on transcriptomic data**. The bar graph was constructed using GraphPad Prism 7 software. Values (mean ± SD, n = 3 independent replicates) were derived from FPKM values in the transcriptomic data (Table S4). The examined developmental stages included zygotes (*A*), trochophore larvae (*B*), veliger larvae (*C*), umbo larvae (*D*), creeping larvae (*E*), single pipe larvae (*F*), and juvenile clams (*G*). Visual maps illustrating the morphologies of these developmental stages were provided alongside the expression data.

As shown in Figure 3 and Table S4, the expression of putative *opsins* exhibited significant variations throughout the developmental stages of *S. constricta*. When considering the expression patterns of different *opsins* at a specific developmental stage, the zygotes were characterized by strong representation of *Sc_opsin17*, followed by *Sc_opsin27*, *22*, and *7* (Fig. 3*A*). The trochophore larvae and veliger larvae predominantly featured *Sc_opsin7*, followed by *Sc_opsin23*, *17*, and *27* (Fig. 3, *B* and *C*). The umbo larvae exhibited high expression of *Sc_opsin5* and *7*, followed by *Sc_opsin1*, *2*, *17*, and *18* (Fig. 3*D*). The creeping larvae were dominated by *Sc_opsin5*, followed by *Sc_opsin7*, *1*, *18*, *12*, *2*, *17*, and *14* (Fig. 3*E*). The single pipe larvae were represented by *Sc_opsin7*, *5*, and *18*, followed by *Sc_opsin1*, *12*, and *27* (Fig. 3*F*). Finally, the juvenile clams demonstrated dominance of *Sc_opsin7* and *5*, followed by *Sc_opsin1*, *13*, *18*, *27*, and *12* (Fig. 3*G*). Regarding the expression patterns of the same *S. constricta opsin* across different developmental stages (Fig. S3), most *opsins*, including *Sc_opsin1*, *2*, *3*, *4*, *7*, *11*, *17*, *19*, *20*, *22*, *23*, *24*, and *27*, displayed significantly high expression levels in the trochophore larvae and veliger larvae. However, *Sc_opsin5* displayed elevated expression in the umbo larvae, while *Sc_opsin12*, *13*, and *14* exhibited high expression levels in the creeping larvae. *Sc_opsin18* showed high expression in the umbo larvae, creeping larvae, single pipe larvae, and juvenile clams. In contrast, the remaining *opsins* demonstrated relatively low and showed no significant differences among all developmental stages.

**Figure 4:**
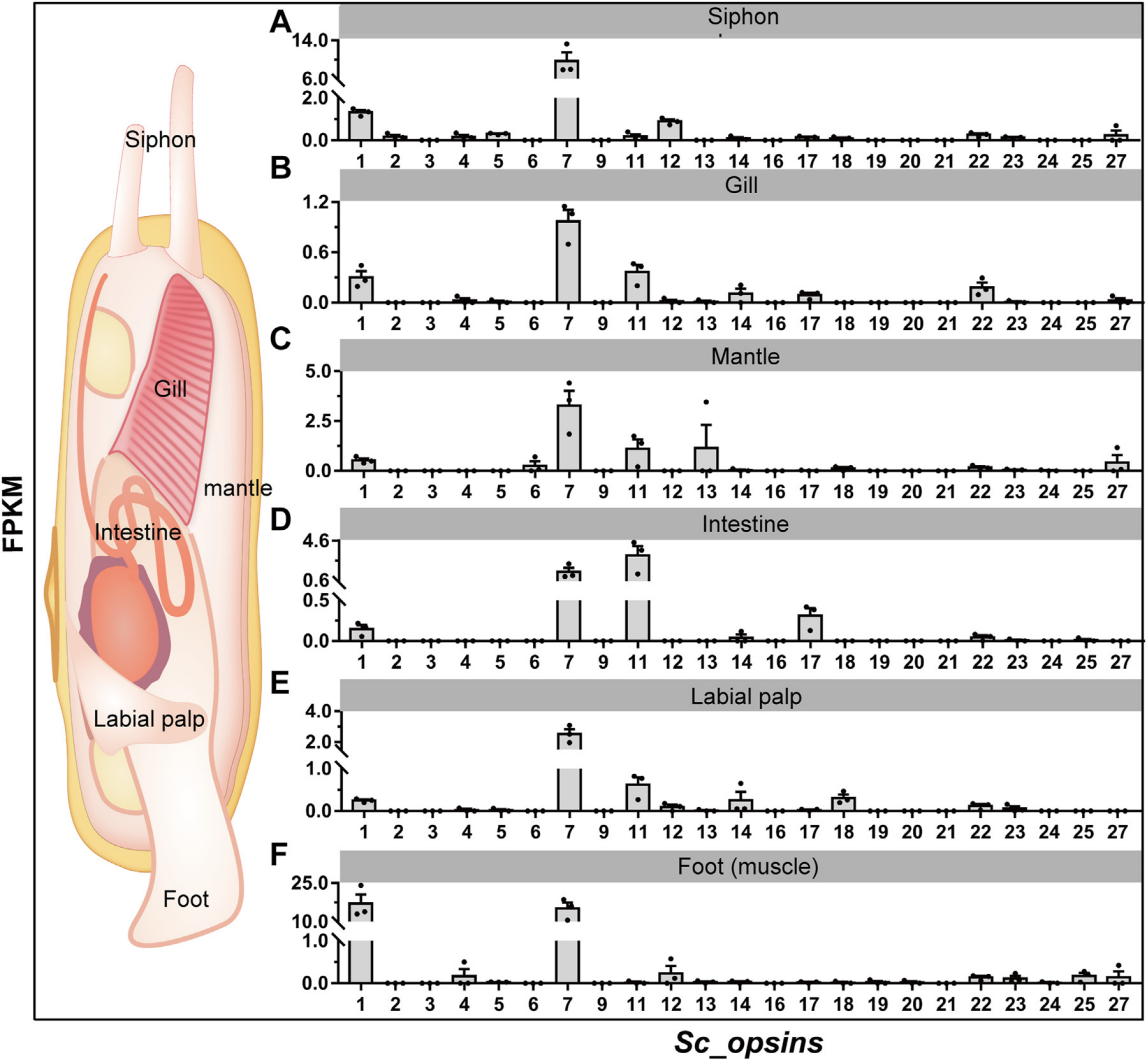
**Expression patterns of 23 putative *S. constricta opsins* in various tissues based on transcriptomic data.** The bar graph was constructed using GraphPad Prism 7 software. Values (mean ± SD, n = 3 independent replicates) were derived from FPKM values in the transcriptomic data (Table S5). The examined tissues included siphon (*A*), gill (*B*), labial palp (*C*), intestine (*D*), mantle (*E*), and foot (*F*). These tissues were obtained from adult *S. constricta* with an average shell length of 55.23 ± 3.31 mm (mean ± SD). A visual map displaying the prominent anatomical features of *S. constricta* was provided alongside the expression data.

As exhibited in Figure 4 and Table S5, the expression of putative *opsins* also displayed significant variations across different tissues of *S. constricta*. Considering the expression patterns of different *opsins* within the same tissue, we found that the siphon exhibited representation of *Sc_opsin7*, followed by *Sc_opsin1* and *12* (Fig. 4*A*). In the gill tissue, *Sc_opsin7* dominated, followed by *Sc_opsin11* and *1*, then *22*, *14*, and *17* (Fig. 4*B*). Within the mantle, *Sc_opsin7* led the expression, followed by *Sc_opsin11* and *13*, then *Sc_opsin27* and *1* (Fig. 4*C*). In the intestine, *Sc_opsin11* dominated, followed by *Sc_opsin7*, and then *Sc_opsin17* and *1* (Fig. 4*D*). The labial palp featured representation by *Sc_opsin7*, followed by *Sc_opsin11*, *18*, *14*, and *1* (Fig. 4*E*). Finally, the foot tissue was characterized by dominance of *Sc_opsin1* and *7*, followed by *Sc_opsin12*, *25*, *27*, and *4* (Fig. 4*F*). Shifting focus to the expression patterns of the same *S. constricta opsin* within distinct tissues (Fig. S4), we observed that *Sc_opsin1*, *7*, and *25* exhibited high expression levels in the foot, while *Sc_opsin5* and *12* were notably expressed in the siphon. *Sc_opsin11* and *17* were prominently expressed in the intestine, and *Sc_opsin18* displayed elevated expression in the labial palp. In contrast, the remaining *opsins* showed relatively low expression levels and no significant differences among all tissues.

### Expression plasticity of four cloned putative *S. constricta opsins*

**Figure 5:**
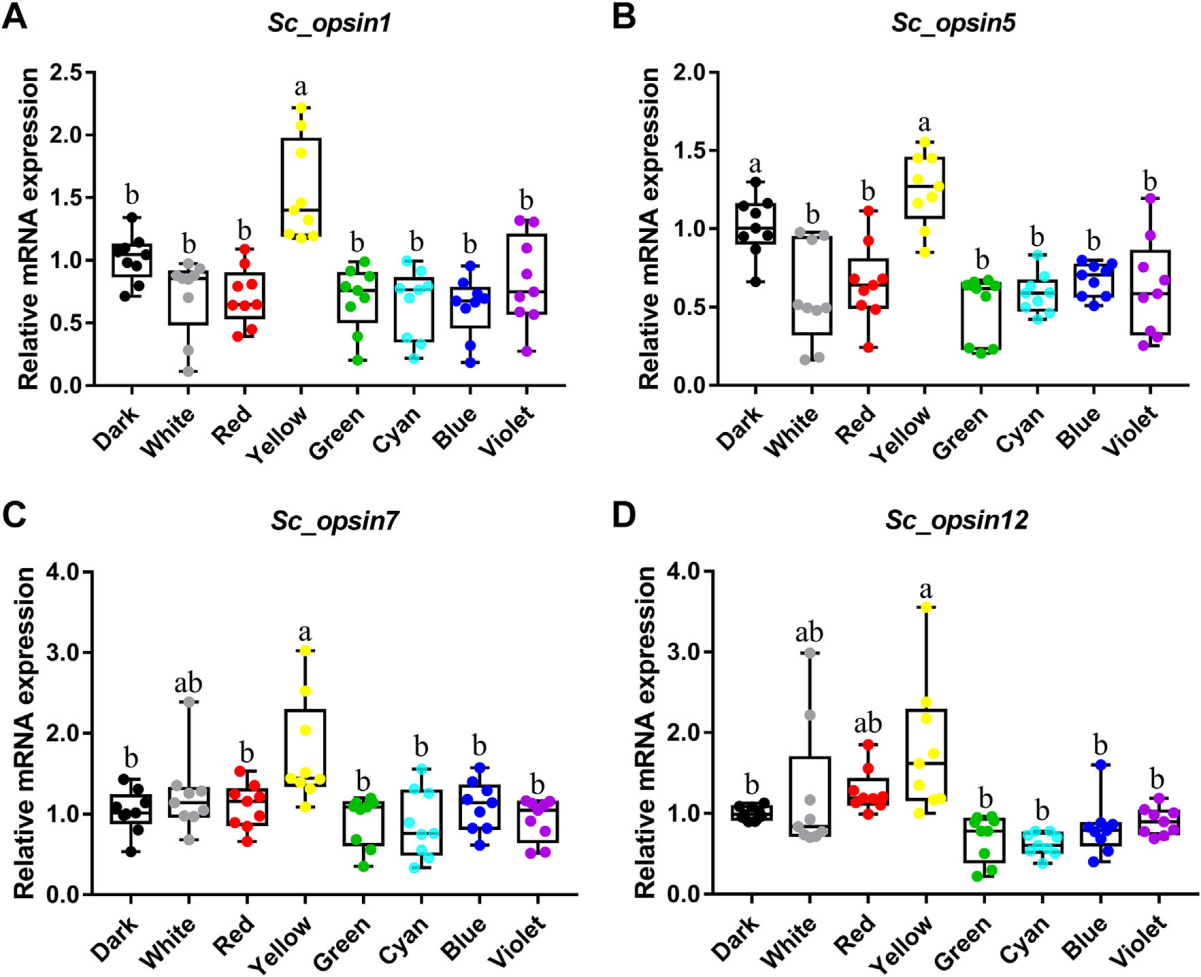
**Expression responses of four cloned putative *S. constricta opsins* to various light spectra.** The four *opsins* were *Sc_opsin1* (*A*), *Sc_opsin5* (*B*), *Sc_opsin7* (*C*), and *Sc_opsin12* (*D*). We exposed juvenile *S. constricta* to light spectra, including *white*, *red*, *yellow*, *green*, *cyan*, *blue*, and *violet light*, with the dark condition as the control. The box plots were constructed using GraphPad Prism 7 software, and the values were obtained through qPCR analysis after 1 week of acclimation under specific light conditions. All values were normalized to those of the control group. The acclimation involved three independent biological experiments, each performed in triplicate. In the graphs, the boxes represent the median and interquartile range, and the whiskers range from the minimum to maximum values. Different letters indicate significant differences at *p* < 0.05. Statistical analysis was performed using SPSS 20 software with one-way ANOVA followed by Tukey’s test.

As depicted in Figure 5, the expression of opsin genes (*Sc_opsin1*, *5*, *7*, and *12*) was influenced by different light spectra. Generally, these four *S. constricta opsins* consistently exhibited the highest expression in juveniles acclimated to yellow light, with only slight variations in response to other light spectra. Specifically, *Sc_opsin1* showed the highest expression under yellow light, while it did not significantly differ under other light spectra (Fig. 5*A*). *Sc_opsin5* displayed the highest expression under yellow light and in the dark, with no significant differences under other light spectra (Fig. 5*B*). *Sc_opsin7* had the highest expression under yellow light, followed by white light, and the lowest expression under other light spectra (Fig. 5*C*). Finally, *Sc_opsin12* exhibited the highest expression under yellow light, followed by white and red lights, with no significant differences under other light spectra (Fig. 5*D*).

### Heterologous expression of four cloned putative *S. constricta* opsins in HEK293T cells

**Figure 6:**
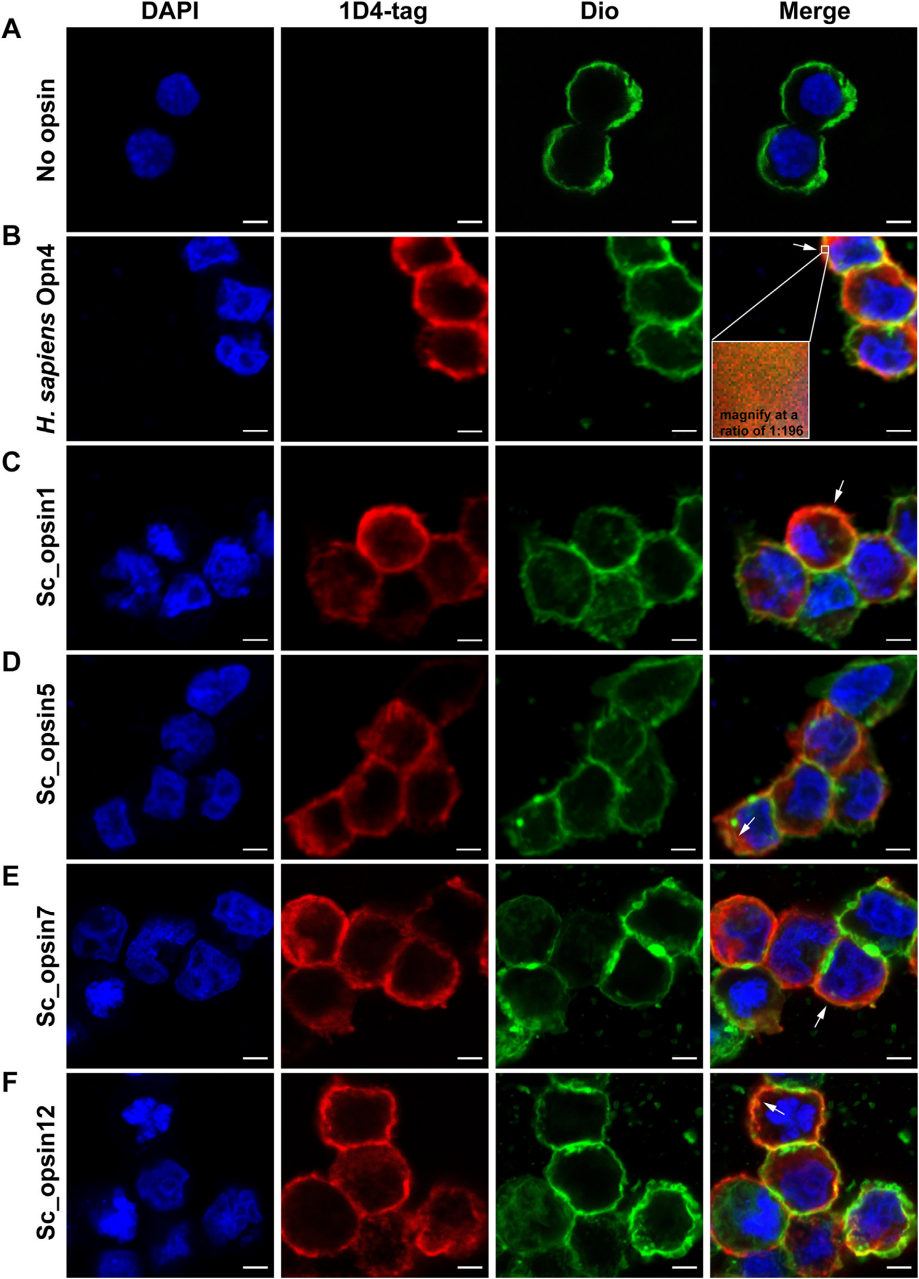
**Heterologous expression of four cloned putative *S. constricta* opsins in HEK293T cells, detected through immunocytochemistry.** Cells were transfected with either the empty vector pcDNA3.1 (*A*, negative control) or recombinant plasmids containing target genes fused with the 1D4 tag. The target genes included *H. sapiens* Opn4 (*B*, positive control), Sc_opsin1 (*C*), Sc_opsin5 (*D*), Sc_opsin7 (*E*), and Sc_opsin12 (*F*). Transfected cells were sequentially stained with the 1D4 antibody (highlighted in *red*), a membrane fluorescent probe (DiO, highlighted in *green*), and a nuclei probe (DAPI, highlighted in *blue*). *Arrows* indicate clear colocalization of opsins with the cell membrane. As an example, the colocalization of *red fluorescence* and *green fluorescence* was magnified in cells expressing *H. sapiens* Opn4 (*B*, *white boxes*). Scale bars: 5 μm.

As shown in Figure 6, the 7 TM proteins of putative *S. constricta* opsins were successfully expressed in HEK293T cells, as confirmed by immunocytochemistry. Specifically, in the negative control group transfected with the empty vector, no red fluorescence associated with the 1D4 tag was detected (Fig. 6*A*). However, in the positive control group transfected with the plasmid containing *H. sapiens* Opn4, red fluorescence was observed and colocalized with the membrane fluorescence (green color) as expected, at least partially (Fig. 6*B*). Similar immunocytochemistry results were found when cells were transfected with the plasmids containing *S. constricta* opsins, as illustrated in Figure 6, *C*–*F*. These results suggest that the HEK293T cells can serve as an effective tool for further investigating the phototransduction function of *S. constricta* opsins, as previously reported (37, 38).

### Phototransduction pathways driven by four cloned putative *S. constricta* opsins

The phototransduction pathway of opsin is mediated by the distinct types of Gα protein, such as Gαq, Gαs, and Gαi, with which it is coupled. In particular, the Gαq cascade leads to an elevation in cytoplasm Ca^2+^ levels, the Gαs cascade causes an increase in cAMP concentration, while the Gαi/o/t (hereafter referred to as Gαi) cascade results in a decrease in cAMP levels and a slight elevation in cytoplasmic Ca^2+^ concentration. However, it is important to note that pertussis toxins selectively deactivate the Gαi cascade without affecting the Gαq or Gαs cascades. To clarify the phototransduction pathways of putative *S. constricta* opsins and their coupled Gα proteins, real-time monitoring of Ca^2+^ and cAMP levels was conducted in HEK293T cells expressing *S. constricta* opsins when exposed to light stimulation. It’s worth noting that as the Gα protein evolution is conserved (Fig. S2), no *S. constricta* Gα proteins were transfected into the HEK293T cells.

**Figure 7:**
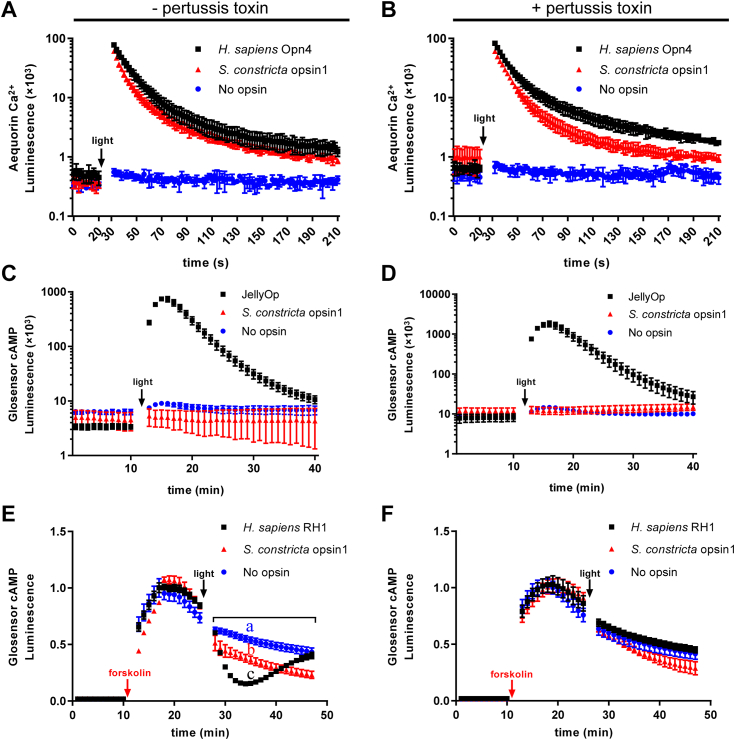
**Phototransduction pathway driven by*****S. constricta*****opsin1.** It is detected by the luminescence of Ca^2+^ (*A* and *B*) and cAMP (*C–F*) levels upon exposure to light. Cells transfected with the empty vector (no opsin) served as the negative control. Cells transfected with *H. sapiens* Opn4 were used as the positive control to measure the luminescence of Ca^2+^ increase (*A* and *B*). Cells transfected with JellyOp served as the positive control to detect the luminescence of cAMP increase (*C* and *D*), while cells transfected with *H. sapiens* RH1 were used as the positive control to detect the luminescence of cAMP decrease (*E* and *F*). The mean luminescence values (mean ± SD) for each treatment were derived from one representative of three independent biological experiments, each with three technical replicates. The other two biological replicates are shown in Fig. S5. Following an initial equilibration period, as indicated in the panels, cells were exposed to *white light* at approximately 20 μmol/m^2^/s for 5 s. Additionally, cells were treated with either (−) (*A*, *C*, and *E*) or (+) (*B*, *D*, and *F*) pertussis toxin, which specifically inhibits the Gαi but not the Gαs or Gαq-coupled signal pathway. To measure the Gαi cascade (*E* and *F*), cells were pre-treated with forskolin to artificially elevate cAMP levels before light exposure. The statistical analysis for comparing luminescence values among different trials in panel *E* was conducted using repeated measures ANOVA with SPSS 20 software, and significant differences were denoted by different letters (*p* < 0.05).

**Figure 8:**
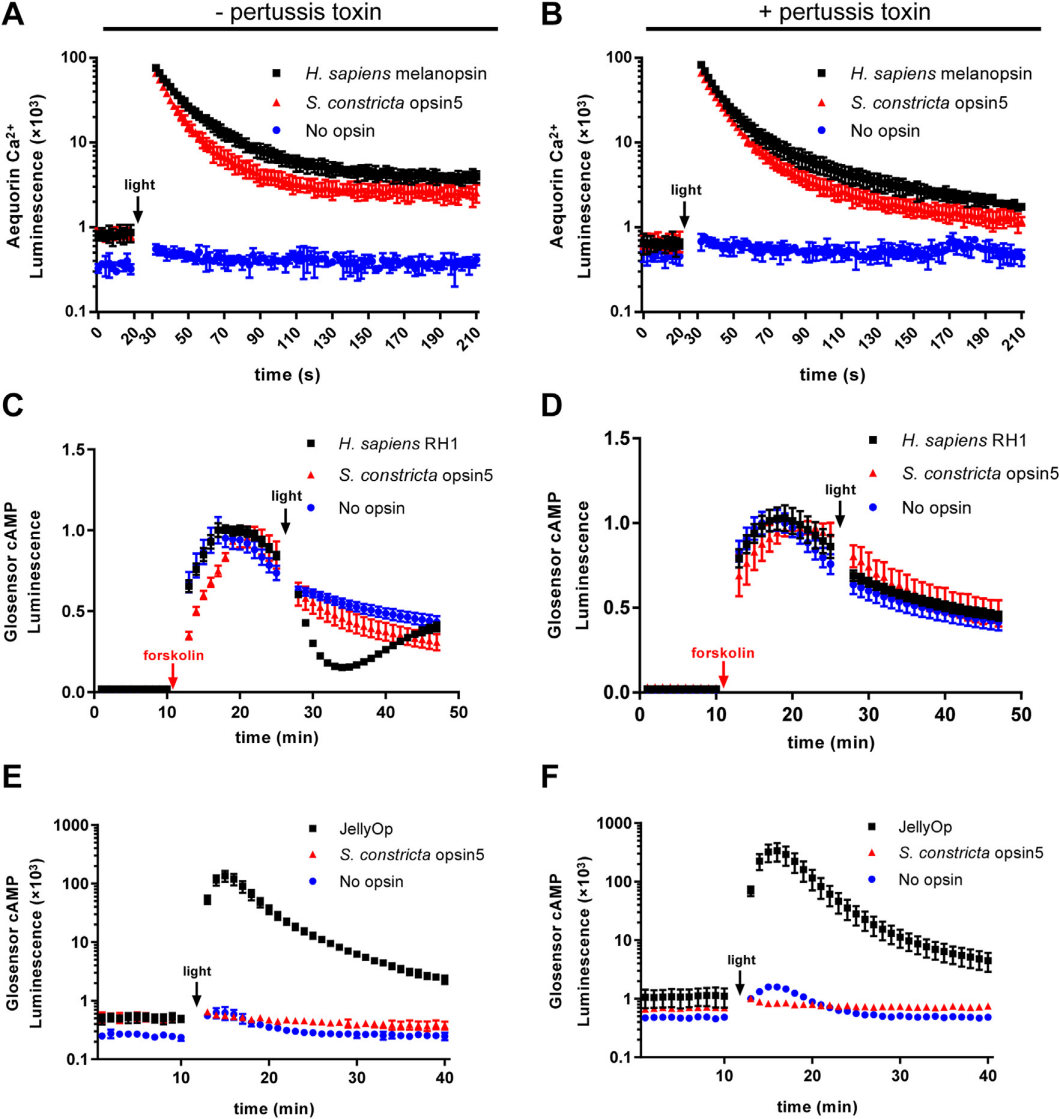
**Phototransduction pathway driven by*****S. constricta*****opsin5.** It is detected by the luminescence of Ca^2+^ (*A* and *B*) and cAMP (*C–F*) levels upon exposure to light. The detailed figure information was identical to that presented in Figure 7. The other two biological replicates are shown in Fig. S6. Notably, the values of the negative and positive controls in panels *A* and *B*, as well as *E* and *F*, are duplicated from Figure 7, respectively.

**Figure 9:**
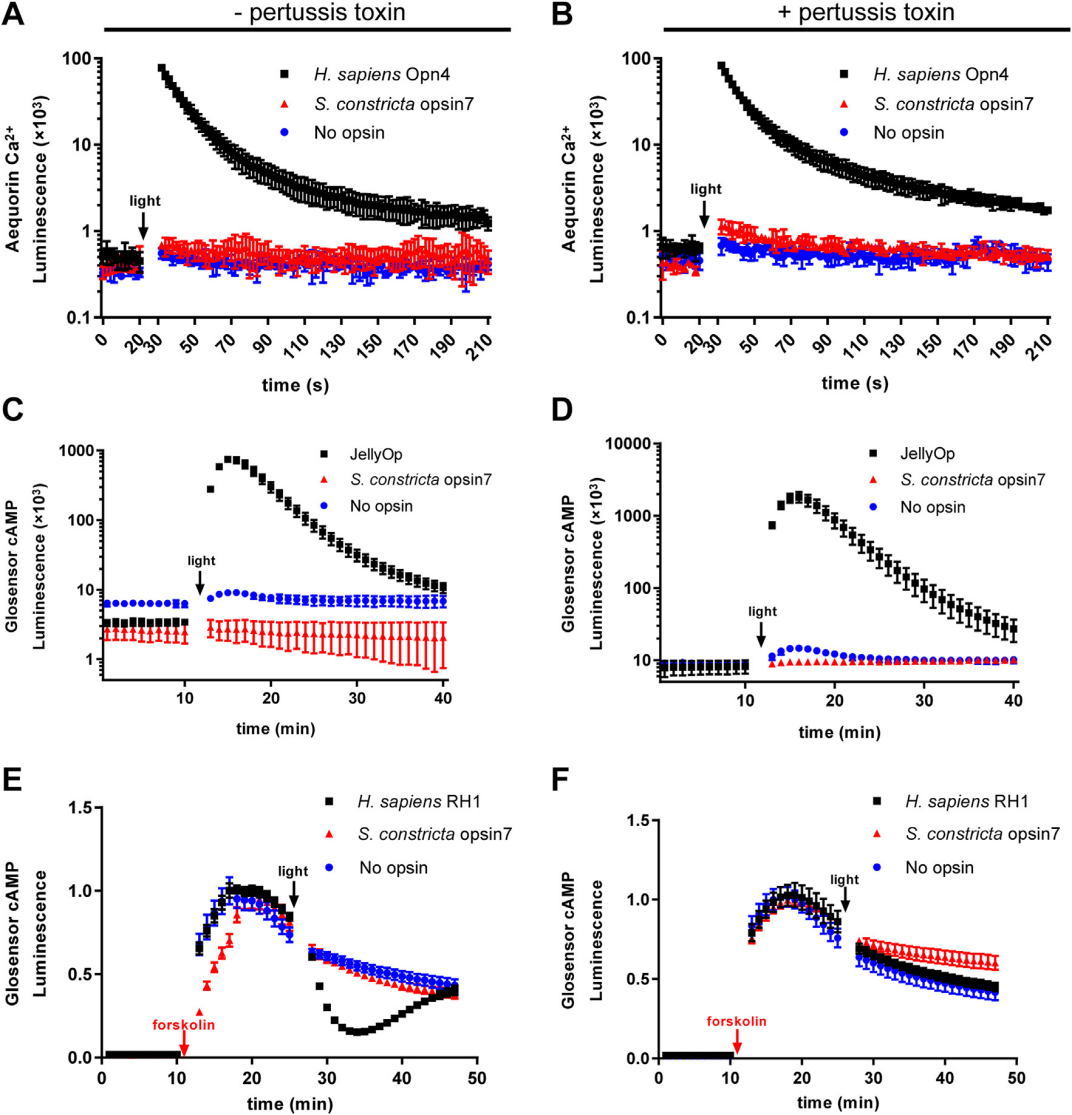
**Phototransduction pathway driven by*****S. constricta*****opsin7.** It is detected by the luminescence of Ca^2+^ (*A* and *B*) and cAMP (*C–F*) levels upon exposure to light. The detailed figure information was identical to that presented in Figure 7. The other two biological replicates are shown in Fig. S7. Notably, the values of all negative and positive controls are duplicated from Figure 7, respectively.

**Figure 10:**
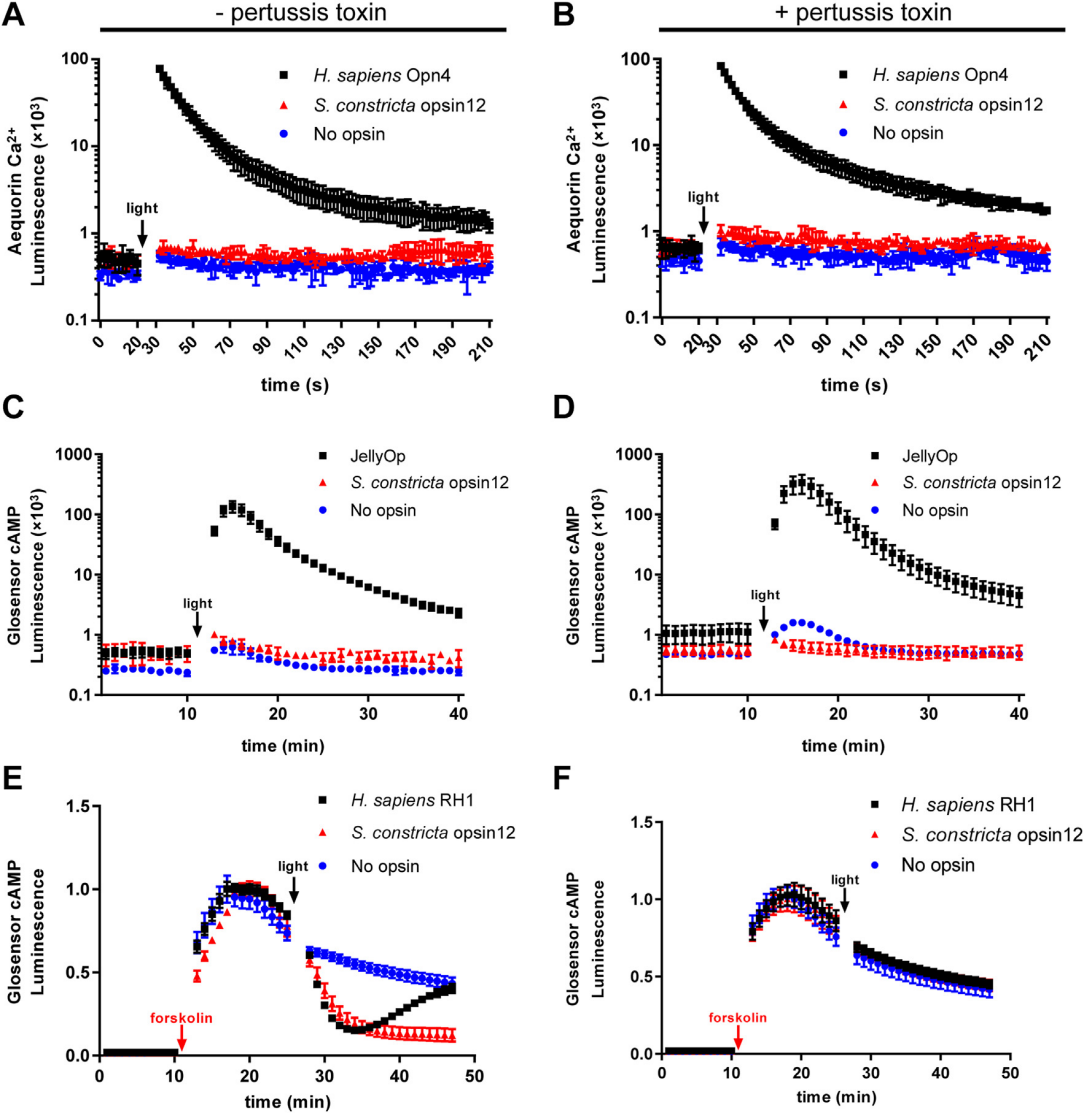
**Phototransduction pathway driven by*****S. constricta*****opsin12.** It is detected by the luminescence of Ca^2+^ (*A* and *B*) and cAMP (*C–F*) levels upon exposure to light. The detailed figure information was identical to that presented in Figure 7. The other two biological replicates are shown in Fig. S8. Notably, the values of negative and positive controls in panels *A* and *B*, as well as *E* and *F*, are duplicated from Figure 7, respectively. The values of negative and positive controls in panels *C* and *D* are duplicated from Figure 8, respectively.

In terms of the Gαq cascade, the cytoplasmic Ca^2+^ levels were significantly increased (>100,000 fold) in HEK293T cells expressing *H. sapiens* Opn4 when exposed to light irradiation, as expected, compared to the negative control (Figure 7, Figure 8, Figure 9, Figure 10*A*). Similar results were observed in cells expressing Sc_opsin1 (Fig. 7*A*) and Sc_opsin5 (Fig. 8*A*) but not in cells expressing Sc_opsin7 (Fig. 9*A*) and Sc_opsin12 (Fig. 10*A*). Meanwhile, the addition of pertussis toxins did not have any effect on the increase of Ca^2+^ in those cells (Figure 7, Figure 8, Figure 9, Figure 10*B*). These findings suggest that Sc_opsin1 and 5 may be functional in phototransduction by coupling with the Gαq protein to increase the cytoplasmic Ca^2+^ levels.

Regarding the Gαs cascade, the cytoplasmic cAMP levels were significantly increased (>100,000 fold) in HEK293T cells expressing JellyOp when exposed to light irradiation, as expected, compared to the negative control (Figure 7, Figure 8, Figure 9, Figure 10*C*). Moreover, the increase in cAMP in those cells was not influenced by the addition of pertussis toxins (Figure 7, Figure 8, Figure 9, Figure 10*D*). However, no significant differences in cAMP changes were observed in cells expressing the four *S. constricta* opsins (Figure 7, Figure 8, Figure 9, Figure 10*C*) when compared to the negative control. Hence, it can be concluded that Sc_opsin1, 5, 7, and 12 do not couple with Gαs protein to increase cAMP levels.

As far as the Gαi cascade is concerned, given the inherently low basal cAMP level in HEK293T cells, the cAMP level was artificially elevated by treating cells with forskolin before light irradiation (Figs. 7–10, *E* and *F*). The cAMP level was significantly decreased in HEK293T cells expressing *H. sapiens* RH1 when exposed to light irradiation, as expected, compared to the negative control (Figure 7, Figure 8, Figure 9, Figure 10*E*). Similar results were observed in the cells expressing Sc_opsin12 (Fig. 10*E*), but not in cells expressing Sc_opsin5 (Fig. 8*E*) and Sc_opsin7 (Fig. 9*E*). Interestingly, in cells expressing *H. sapiens* RH1, there was a subsequent increase in cAMP levels over time, whereas in cells expressing Sc_opsin12, the cAMP levels continued to decrease (Fig. 10*E*). Additionally, the decrease of cAMP in those cells was completely inhibited by the addition of pertussis toxins (Figure 7, Figure 8, Figure 9, Figure 10*F*). These results suggest that Sc_opsin12 may play a functional role in phototransduction by coupling with the Gαi protein to decrease the cAMP levels. Notably, the artificially elevated cAMP levels were also decreased significantly (*p* < 0.05) in cells expressing Sc_opsin1 when exposed to light irradiation (Fig. 7*E*). However, this decrease was not pronounced as observed in the positive control. This suggests that, in addition to mediating the Gαq cascades, Sc_opsin1 may also play a role in mediating the Gαi cascades to decrease the cAMP level to some extent.

### Critical aa for phototransduction function of putative *S. constricta* opsins

**Figure 11:**
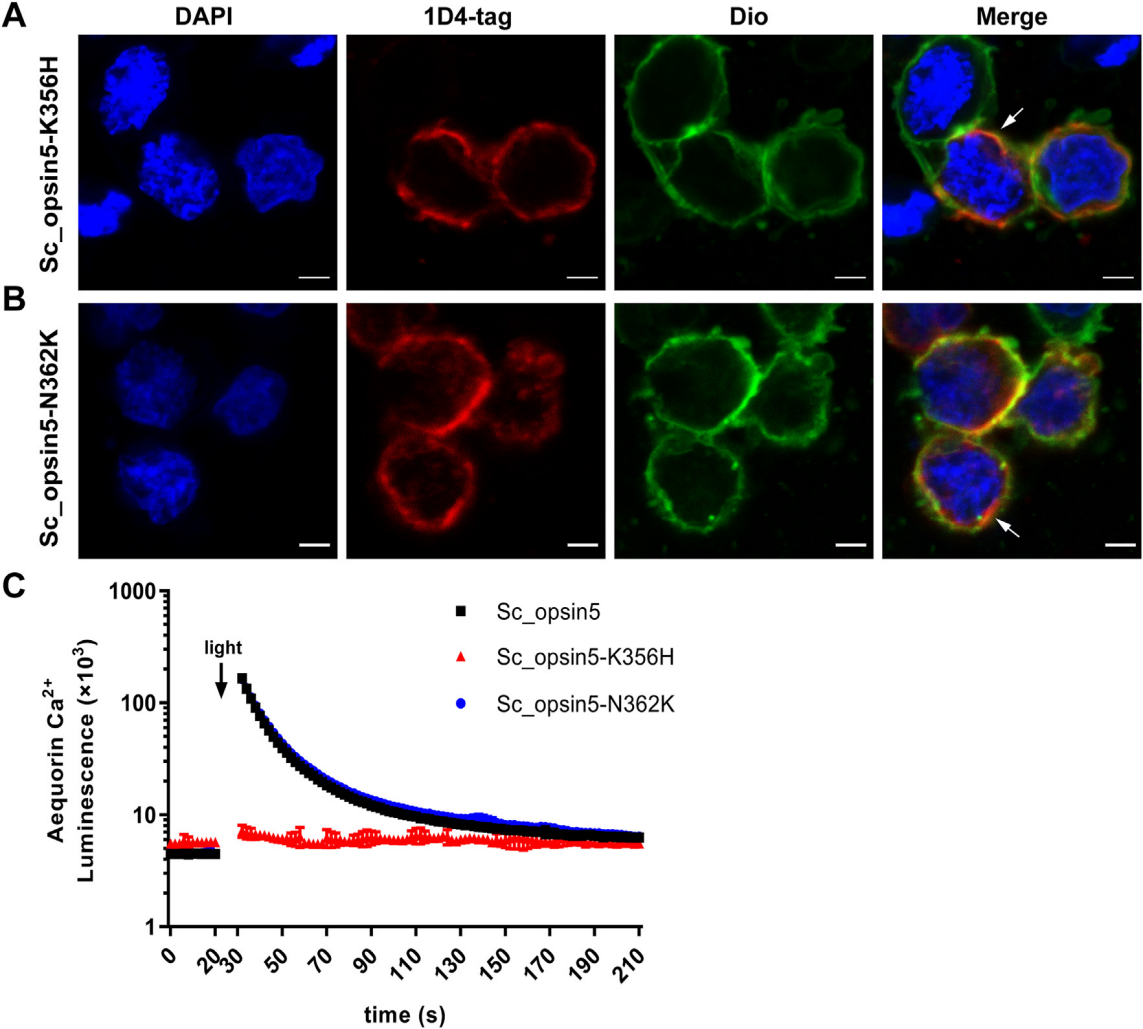
**Heterologous expression of *S. constricta* opsin5 (Sc_opsin5) mutants and their phototransduction sensitivity.** The heterologous expression is carried out in HEK293T cells, detected through immunocytochemistry (*A* and *B*), while the phototransduction sensitivity is determined by measuring luminescence of Ca^2+^ levels upon exposure to light (*C*). The mutants included Sc_opsin5-K356H and N362K, where K356H represented the substitution of K at TM7 of Sc_opsin5 with histidine (H), and N362K represented the substitution of asparaginate (N) at the NPXXY motif of Sc_opsin5 with K. *A* and *B*, transfected cells were sequentially stained with the 1D4 antibody (highlighted in *red*), a membrane fluorescent probe (DiO, highlighted in *green*), and a nuclei probe (DAPI, highlighted in *blue*). *Arrows* indicated clear colocalization of opsins with the cell membrane. Scale bars: 5 μm. *C*, cells transfected with wild-type Sc_opsin5 served as the positive control. The mean luminescence values (mean ± SD) for each treatment were derived from one representative of three independent biological experiments, each with three technical replicates. The other two biological replicates are shown in Fig. S9. After an initial equilibration period, as indicated in the panel, cells were exposed to white light at approximately 20 μmol/m^2^/s for 5 s.

Using the Sc_opsin5 as an example, two mutants, K356H (altering K356 at TM7) and N362K (modifying N362 at NPXXY), were constructed and subsequently transfected into HEK293T cells. As shown in Figure 11, *A* and *B*, the two mutant proteins could be successfully expressed in HEK293T cells. Regarding the function of signal transduction, the Gαq-mediated increase in Ca^2+^ remained efficiently functional in cells expressing the Sc_opsin5 mutant N362K (Fig. 11*C*). In contrast, the same increase in Ca^2+^ was not observed in cells expressing the K356H mutant (Fig. 11*C*). This result suggests that the retinal binding site is also essential for the phototransduction function of *S. constricta* opsins, at least for Sc_opsin5.

### Spectral sensitivity of phototransduction function of putative *S. constricta* opsins

**Figure 12:**
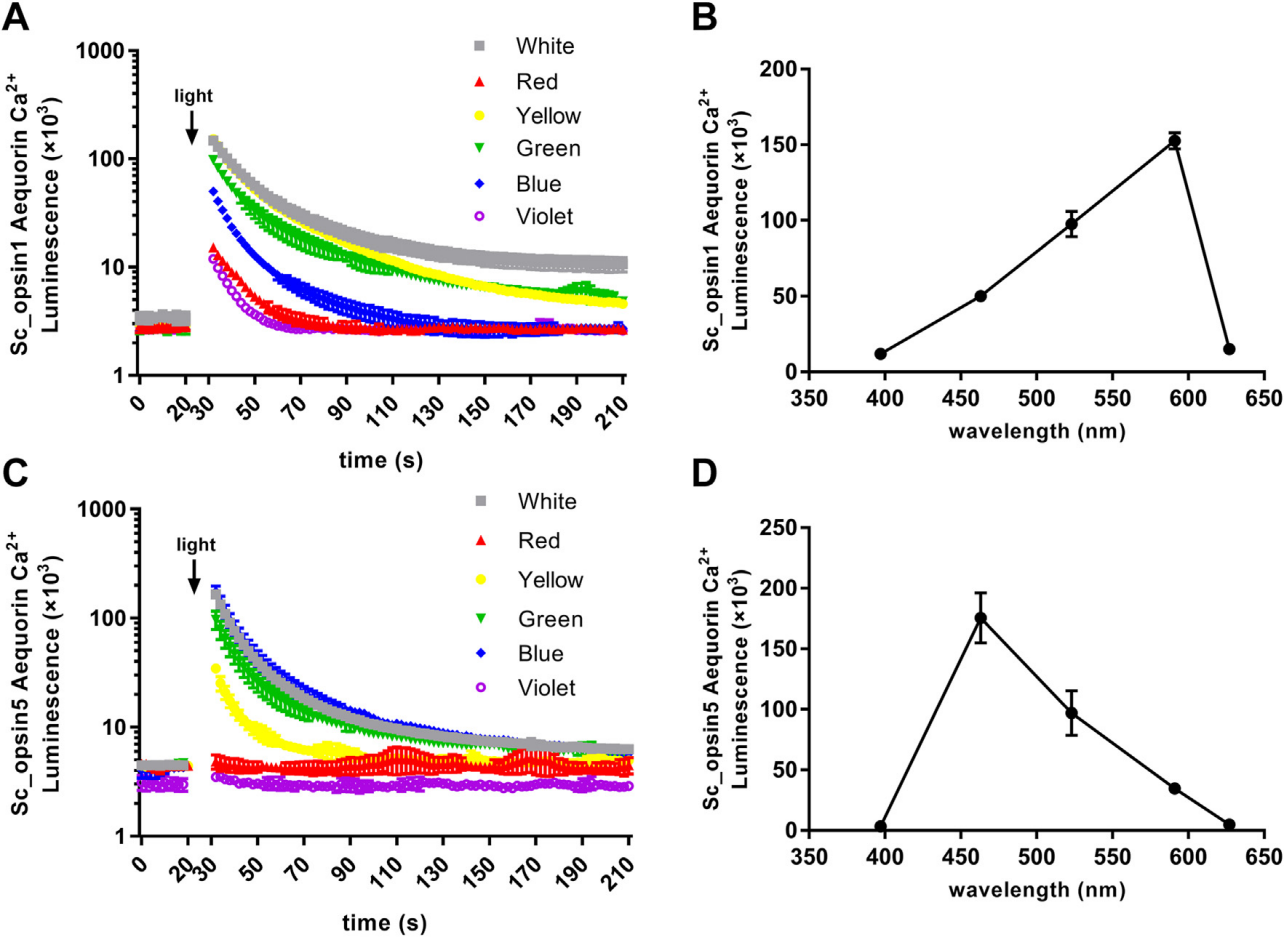
**Phototransduction sensitivity driven by *S. constricta* opsin1 (Sc_opsin1) and *S. constricta* opsin5 (Sc_opsin5), as detected by the luminescence of Ca^2+^ levels upon exposure to different light spectra.***A* and *B*, Sc_opsin1. *C* and *D*, Sc_opsin5. The light spectra included *white*, *red*, *yellow*, *green*, *blue*, and *violet*. The mean luminescence values (mean ± SD) for each treatment were derived from one representative of three independent biological experiments, each with three technical replicates. The other two biological replicates are shown in Fig. S10. After an initial equilibration period, as indicated in the panels, cells were exposed to the respective light spectra at approximately 20 μmol/m^2^/s for 5 s. Meanwhile, to enhance the interpretation of the response-versus-wavelength relationship, *straight lines* were used to connect the maximum values under each light irradiation condition (*B* and *D*).

Using the *S. constricta* Gq-opsins (Sc_opsin1 and 5) as examples, cells expressing Sc_opsin1 or 5 were exposed to flashes of different light spectra. As depicted in Figure 12*A*, compared to the initial level, the Ca^2+^ level in cells expressing Sc_opsin1 showed the highest increase when flashed with white, yellow, and green lights (>100,000 fold), followed by blue light (>50,000 fold), and then red and violet lights (>10,000 fold). Similarly, as shown in Figure 12*C*, compared to the initial level, the Ca^2+^ level in cells expressing Sc_opsin5 exhibited the highest increase when flashed with white, blue, and green lights (>100,000 fold), followed by yellow light (>50,000 fold), while no responses were observed when flashed with red and violet lights. Furthermore, to enhance the interpretation of the response versus wavelength relationship, we presented the averages of the maximum values under each light irradiation condition in Figure 12, *B* and *D*, connecting them with straight lines. These results suggest that the phototransduction intensities of *S. constricta* opsins are highly dependent on the light spectrum, even for the same types of opsins.

## Discussion

Marine bivalves represent a highly diverse and evolutionarily successful group of organisms in the marine ecosystem. Confronted with the challenging light environment, these organisms have evolved a wide range of photoreceptor organs (39), making them a valuable source for studying the evolutionary adaptation to marine photoecology. In the present study, our aim was to investigate the photoreceptor molecule opsins and their underlying phototransduction mechanisms in a representative eyeless species, *S. constricta*.

### Molecular system of phototransduction is conserved in *S. constricta*

Gene duplication, followed by subsequent functional divergence of opsins, has played a crucial role in expanding the photoreceptive capabilities of animals (40, 41). In this study, the opsin genes were found to be abundant in the five marine bivalves under investigation, as shown in Table S1 and Fig. S1. This finding underscores the significance of light in their developmental processes and survival. Notably, a novel opsin group known as xenopsin was identified and found to be significantly expanded in *S. constricta* compared to the other four bivalves, while the c-opsin was absent. Xenopsins have been recently identified in several protostomes, including mollusks, brachiopods, rotifers, platyhelminths, annelids, and cnidarians (5, 11, 37, 42). It has been discovered that xenopsin exhibits similarity to c-opsin and is functional in coupling with Gαi protein to drive the light signal transduction in flatworms (37). Furthermore, unlike other bivalves, *S. constricta* lacks Go-opsin, which is initially identified in ciliary photoreceptors. This opsin type induces hyperpolarization through a cGMP-gated channel, akin to vertebrate rhodopsins and cone opsins (18, 43, 44). Therefore, it is speculated that the expanded presence of xenopsins within *S. constricta* may potentially compensate for the absence of other opsin types.

Additionally, Gq-opsins have been recognized as the primary photoreceptors involved in phototransduction in invertebrates (19, 45, 46). In this study, two Gq-opsins (Sc_opsin1 and 5) were identified in the genome of *S. constricta*, whereas the other four marine bivalves investigated here possessed 4 to 6 Gq-opsins. This disparity may be also attributed to the significant expansion of xenopsins in *S. constricta*, which likely alleviated the selective pressure on Gq-opsins. Overall, the distinct differentiation in the composition of opsin families among the five marine bivalves might be closely linked to their specific light environments. This, in turn, could contribute to the emergence of lineage-specific biological features.

Moreover, homologous sequences of retinochrome, peropsin, and neuropsin were also identified in the *S. constricta* genome. Previous studies have demonstrated that retinochrome and peropsin serve as photo-isomerases, specifically converting all-trans retinal into cis-retinal to restore opsin to its active state, without interacting with any type of G proteins (16, 47, 48). Therefore, a similar function of photo-isomerases might also occur for *S. constricta*’s retinochrome and peropsin. On the other hand, neuropsin has been shown to be a bistable UV-responsive photopigment (49) and is expressed in the mammalian retina, brain, and even ears (50). However, its function in marine bivalves remains unclear thus far.

Importantly, the phototransduction pathway of a specific opsin is mediated by distinct types of Gα proteins to which it is coupled. In this study, a total of 6 Gα proteins belonging to five classes were identified. All of them contained the corresponding conserved C-terminal sequences found in mammalian Gα proteins, indicating the conserved evolution of the Gα protein and their functions. Taken together with the afore-mentioned results of *S. constricta* opsins, it can be concluded that a functional and complex photosensitive system likely exists in this eyeless bivalve species.

### Expression of *S. constricta opsins* is dependent on developmental stages and tissues

Investigating gene expression patterns, especially across various developmental stages and tissues, can provide valuable insights into predicting their potential functions. In teleosts, it has been observed that opsins’ expression varies significantly during development, likely as an adaptation to changes in the light environment (51, 52). A similar phenomenon was noted in *S. constricta* opsins, which might also be closely linked to the shifting light conditions throughout its development. During the life cycle of *S. constricta*, it progresses through distinct stages of “planktonic-metamorphosis-attachment-benthic”, marked by significant changes in light exposure, transitioning from relatively strong and longer wavelength spectra to dimmer and shorter wavelength spectra. Notably, a majority of *S. constricta* opsins exhibited high expression levels in the trochophore larvae and veliger larvae stages, which are critical for the bivalve’s metamorphosis. Additionally, specific opsins were also prominently expressed in other corresponding developmental stages of *S. constricta*. These findings imply that opsins likely play a crucial regulatory role in *S. constricta*’s development. Collectively, these results underscore the importance of an appropriate light environment for the successful breeding and cultivation of *S. constricta*.

Meanwhile, as a representative eyeless marine bivalve, *S. constricta* displays pronounced photosensitivity (25, 26, 27, 28), although the underlying photosensitive tissues have yet to be elucidated. Recently, studies have demonstrated the photosensitivity of the eyeless *C. gigas* (53). Subsequently, a rhodopsin-like gene was identified in *C. gigas*, showing high expression in the mantle tissue. Notably, the photosensitive capability of *C. gigas* was significantly hindered upon knockdown of this gene (22). Likewise, sea urchin tube feet, considered their photosensitive organs, express r-opsins and the paired box 6 (PAX6) protein (54, 55). Furthermore, certain eyeless cnidarians have exhibited photosensitivity, with opsin expression linked to extraocular photoreception (56, 57). Hence, the tissues expressing opsins could be potentially serve as sites for nonvisual photosensitivity in organisms. In this study, more than four opsins were found to be highly expressed in peripheral tissues like the mantle, siphon, and foot of *S. constricta*, suggesting their potential role as photosensitive tissues in this bivalve. Notably, several opsins also displayed high expression in internal tissues like the intestine and labial palp. This suggests that these opsins might have roles beyond light sensitivity in *S. constricta*, such as functions related to taste, hearing, and thermosensation, as indicated in other studies (58, 59, 60). Additionally, only *Sc_opsin7* exhibited relatively higher expression across all tissues, underscoring its pivotal role in *S. constricta*’s photosensitive system.

### Responsiveness of *S. constricta opsins* to light spectra is evident

It was hypothesized that if *S. constricta* opsins were functional in phototransduction, their expression would be influenced by the environmental light spectra, as reported in other aquatic animals (61, 62, 63). As anticipated, the expression of the four cloned *S. constricta opsins* exhibited distinct variations in juvenile *S. constricta* after 1 week of acclimation to different light spectra. Remarkably, their highest expression levels were consistently observed under yellow light conditions. This finding aligns with our previous study, which demonstrated that the growth, feeding rate, digestion ability, and antioxidant capability of juvenile *S. constricta* peaked when cultured under yellow light (26). Similar patterns have been documented in other studies, where opsin expression showed a positive correlation with growth (61, 62). This relationship could be attributed to the activation of the phototransduction pathway when opsin perceive light, subsequently triggering feeding behavior and other growth-related metabolic processes. Supporting this hypothesis, increased transcripts of growth hormone (GH), insulin-like growth factor (IGF-Ⅰ), and neuropeptide Y (NPY) were observed in *Epinephelus malabaricus* when reared under beneficial blue light (61).

However, in juvenile *S. constricta*, the expression levels of these four opsins generally remained unchanged when exposed to light spectra other than yellow light. This could be attributed to species-specific variations in opsin responses or potentially influenced by the duration of acclimation. For instance, reports indicate that opsin expression in African cichlids *Metriaclima mbenji* can rapidly change in response to varying light environments within a short span of 3 days (64). In contrast, certain teleosts such as *Cyprinella lutrensis* requires a longer acclimation period (30 days) to adapt to external light changes (65).

Additionally, we found distinct variations in the expression levels of *S. constricta opsins* between white and yellow light conditions, despite white light containing all spectra, including yellow. This phenomenon might arise from the competition or inhibition effects that different light spectra within white light, except for yellow, could have on influencing the perception of the yellow spectrum by *Sc-opsins*. Alternatively, it’s possible that the intensity of the yellow spectrum within white light is relatively weaker compared to that of pure yellow light. This disparity in intensity could also contribute to the observed variations in the expression of *Sc-opsins*, as previously reported in other aquatic animals (66).

### Phototransduction function of *S. constricta* opsins is intricately mediated by specific Gα proteins

To date, investigations into the phototransduction pathways driven by marine bivalve opsins have predominantly focused on identifying critical components, such as Gα proteins and downstream enzymes (17, 18, 67). However, direct evidence of Gα protein cascades remains limited. To our knowledge, only one study has reported the existence of such evidence in the context of the scallop *P. yessoensis* opsin2 (AB006455.1) (68). Specifically, this study revealed a light-dependent increase in cAMP level observed in HEK293T cells expressing the scallop *P. yessoensis* opsin2. Importantly, this response remained unaffected by the treatment of pertussis toxin (68).

In this study, we confirmed that Sc_opsin1 and 5 belong to the Gq-opsin category, coupling with Gαq protein, while Sc_opsin12 falls into the Gi-opsin classification, coupling with Gαi protein. Additionally, Sc_opsin1 was found to function partially as a Gi-opsin as well. Similar observations have been reported previously, demonstrating that, like other GPCRs, opsins are capable of activating different members of Gα proteins, thereby initiating both Ca^2+^ and cAMP signal transduction cascades (69). This diversity in the biological functions of opsins could be closely linked to their ability to couple with multiple Gα proteins, leading to different phototransduction pathways with varying intensities and kinetics (68, 70). However, in cells expressing Sc_opsin7, no luminescence related to cAMP and Ca^2+^ changes were detected upon exposure to light. This suggests that Sc_opsin7 might not couple with any Gα protein cascade. This observation aligns with the phylogenetic analysis, which places Sc_opsin7 within the retinochrome group. This group functions as photo-isomerases, without coupling with any type of G proteins (16, 47, 48). However, it cannot be excluded that poor surface expression of Sc_opsin7 in HEK293T cells might mask its potential function in phototransduction, which requires further investigation.

Furthermore, although both *H. sapiens* RH1 and Sc_opsin12 led to a light-dependent decrease in cAMP levels in HEK293T cells, a distinct difference in kinetics was observed. A similar phenomenon has been reported in the lamprey *Petromyzon marinus* parapinopsin, where blue light irradiation induced a continuous decrease in cAMP level in HEK293S cells (71). This divergence in kinetics might arise from the incompatibility of the light signal termination mechanism (such as GPCR kinases and arrestins) in HEK293T/S cells with exogenous opsins. Another explanation could be that the activated Sc_opsin12/*P. marinus* parapinopsin remains stable or bistable in the phototransduction system (10). Moreover, reports suggest that to adapt to dim light, vertebrate rod opsins exhibit a prolonged signaling state, resulting in slower response kinetics, transduction pathway, and recovery of visual sensitivity after bleaching, compared to cone opsins. This adaptation greatly enhances their photosensitivity (72). Based on these observations, we speculated that the extended and sustained Gα protein cascade triggered by Sc_opsin12 might serve similar roles to those of vertebrate rod opsins. This prolonged response could be advantageous for amplifying photosensitivity to detect dim light within the natural habitat of *S. constricta*.

### K residue at TM7 is essential for the phototransduction function of *S. constricta* opsins

As anticipated, the Sc_opsin5-K356H mutant was unable to induce light-dependent changes in Ca^2+^ levels. This deficiency can be attributed to the fact that the K residue at TM7 serves as the binding site for the opsin’s chromophore. It’s worth noting that studies on the evolution of opsins have proposed that the common ancestor of opsins lacked the chromophore-binding site represented by the K residue. This evolutionary development occurred later in the history of opsins (73). Supporting this hypothesis, placopsins (73) and pseudopsins (74) are both devoid of the critical K residue. Similarly, in our study, two *S. constricta* xenopsins (Sc_opsin18 and 23) also lacked the K residue at the retinal-binding site. Given the multifaceted functions of opsins, including roles unrelated to light sensitivity such as mechanoreception, chemoreception, and thermoception (7, 75), the perspective that the retinal-binding domain is an absolute requirement for opsins has been called into question.

Regarding the NPXXY domain, numerous studies have reported its association with G protein binding (9, 76). However, our current study indicates that the Gαq protein cascade remains effectively driven by the Sc_opsin5-N362K mutation. This suggests that, at least for Sc_opsin5, the NPXXY domain might be not essential for the photosensitivity of *S. constricta* opsins. Nevertheless, it is imperative to conduct further studies to validate this hypothesis. One potential approach is to completely delete this domain and observe the resulting effects. Therefore, beyond comprehensive sequence alignment and evolutionary analysis, it is essential to conduct relevant biochemical or cellular experiments to rigorously ascertain the exact function or functional domains of these new putative opsins.

### Phototransduction function of *S. constricta* opsins exhibits sensitivity to different light spectra

It is widely recognized that each opsin possesses a specific absorption for an optimal light spectrum, known as the wavelength of maximum absorbance (λmax). The precise determination of λmax often requires direct micro-spectrometric measurements in the opsin’s natural environment or by measuring the λmax of the recombinant visual pigment through *in vitro* purification (21, 77). However, these methods remain challenging for GPCR proteins like opsins due to their extremely low expression levels *in situ* and *in vitro*. Given the high sensitivity achieved through luminescence detection, our study directly measured the photosensitivity of two representative *S. constricta* Gq-opsins (Sc_opsin 1 and 5) in response to various light spectra by monitoring Ca^2+^ luminescence in HEK293T cells. As anticipated, the increased in Ca^2+^ levels significantly differed in cells expressing Sc_opsin 1 or 5 when exposed to different light spectra. This approach proved to be effective and suitable for predicting opsin λmax. Specifically, both Sc_opsin1 and 5 exhibited the highest photosensitivity under white light, which might be attributed to the comprehensive spectral composition of white LED light. Distinctly, besides white light, Sc_opsin1 displayed increased photosensitivity under yellow and green lights, while Sc_opsin5’s photosensitivity was heightened under blue and green lights. These outcomes suggest that the Sc_opsin1’s preference for λmax might be approximately within the range of ∼515 to 596 nm, while Sc_opsin5’s λmax could lie around ∼440 to 535 nm.

Furthermore, while red and violet lights induced a slight increase in Ca^2+^ levels in cells expressing Sc_opsin1, no response was observed in cells expressing Sc_opsin5. Although it’s widely accepted that nearly all opsins exhibit a significant shoulder in the blue-violet region of the absorption spectra, the elevated absorbance in the blue-violet range may not be solely due to photopigment activity. It could potentially result from varying amounts of an “endogenous absorbing” pigment (78). Therefore, *S. constricta* opsins, including opsin5, may also exhibit absorbance in the blue-violet part when examined in the absorption spectra, similar to other photopigments. However, it’s worth noting that the absorbance spectrum of the opsin protein is distinct from its spectral sensitivity during activation. The results in Figure 12 can only suggest the functional responses of Sc_opsins under various light irradiations. Taken together, these findings imply that Sc_opsin1 and 5 may work in tandem to guide the Gαq cascade in *S. constricta*, enabling it to adapt to the dynamic light environment of its aquatic habitat.

In summary, this study presents the first molecular and functional evidence for nonvisual photosensitivity in marine bivalves. The findings strongly underscore the vital roles that nonvisual photosensitivity plays in the growth and development of *S. constricta*. Despite lacking conventional eyes, *S. constricta* employs opsins to discriminate light spectra, thereby guiding the Ca^2+^ and cAMP signaling pathways. Moreover, the outcomes propose the potential existence of a collaborative photosensitive system mediated by opsins in *S. constricta*. This system enables rapid responses to transient or subtle shifts in the external light environment. Beyond advancing our understanding of opsin molecular biology and evolution, these results contribute valuable insights into the evolutionary adaptation of marine bivalves to their aquatic photoecology and shed light on their light requirement throughout their life histories.

## Experimental procedures

All animal experiments in this study were approved by the Animal Research and Ethics Committees of Ningbo University.

### Genomic identification of putative *S. constricta* opsins and Gα proteins

In terms of the putative *S. constricta* opsins, two approaches were employed. First, the homologous genes were obtained by blasting against the *S. constricta* genome (23), using the aa sequences of typical opsins from *H. sapiens*, *D. melanogaster*, *Branchiostoma floridae*, *M. yessoensis*, *Argopecten irradians*, and *Euprymna scolopes* (Table S6) as queries. Secondly, all potential opsin sequences were directly retrieved from the *S. constricta* genome based on functional annotation (23). Subsequently, sequences obtained through the afore-mentioned methods with aa identities greater than 98% were excluded, retaining the longest sequence for further analysis. Following this step, the candidate opsins underwent additional validation through searching against NCBI BLAST and were submitted to the GPCRHMM webserver (https://gpcrhmm.sbc.su.se/). Ultimately, only those opsins with clear annotations and containing the essential 7 TM helixes characteristic of typical opsins were considered as putative *S. constricta* opsins.

When it comes to the putative *S. constricta* Gα proteins, similar strategies as mentioned above were employed. Notably, the typical Gα proteins from *H. sapiens* and *D. melanogaster* (Table S6) were selected as queries. The detailed sequences of the putative *S. constricta* opsins and Gα proteins are shown in Table S2.

Furthermore, to elucidate the distribution and divergence of the opsin family in marine bivalves, putative opsins from three other eyeless bivalves *M. mercenaria* (genome accession number: GCF_014805675.1), *C. gigas* (GCF_902806645.1), and *M. philippinarum* (79), as well as one species featuring numerous non-cephalic eyes of *M. yessoensis* (GCF_002113885.1), were identified using similar methods as described above, based on their genomes. Detailed sequences of their putative opsins are given in Table S3.

### Sequence and phylogenetic analyses of putative *S. constricta* opsins and Gα proteins

To provide essential insights for predicting the functions and evolution of the putative *S. constricta* opsins and Gα proteins, we conducted multiple sequence alignment and phylogenetic tree construction. For putative *S. constricta* opsins, the aa sequence alignment was performed using Clustalx 2.1 software. The phylogenetic tree construction was performed using the maximum-likelihood method through SeaView software (PhyML algorithm) (80), and visualized with Figtree v1.4.3 and Adobe Photoshop CS (version 6.0). The branch lengths of the tree topology were computed by minimizing the sum of squared differences between evolutionary and patristic distances. Notably, for the phylogenetic analysis, well-classified typical opsin sequences from representative species were selected as the intergroup, whereas melatonin sequences from *H. sapiens*, *B. floridae*, *P. dumerilii*, *Strongylocentrotus purpuratus*, and *S. constricta* were utilized as the out-group. Additionally, the aa sequences of all genes used for the phylogenetic analysis underwent alignment using Clustalx 2.1 software. Subsequently, the aligned dataset was trimmed to exclude the N- and C-terminal sequences, retaining only the TM helixes and loop regions for further phylogenetic tree construction.

As for the Gα proteins, aa sequence alignment was conducted using Clustalx 2.1 software. The phylogenetic tree construction was performed using MEGA 7 software, employing the maximum-likelihood method based on the JTT matrix-based model. The confidence in the resulting branch topology of the phylogenetic tree was measured through bootstrapping, which involved 1000 iterations.

### Analysis of temporal and spatial expression patterns of putative *S. constricta opsins*

To reveal the potential roles of putative opsins in *S. constricta* development and to identify potential photosensitive tissues, we analyzed their temporal and spatial expression patterns using our previously published transcriptome data. For the temporal expression analysis, transcriptome data from various developmental stages, encompassing zygotes, trochophore larvae, veliger larvae, umbo larvae, creeping larvae, single pipe larvae, and juvenile clams were available at SRA under accession numbers SRR8325910 to SRR8325916 (23). In terms of spatial expression analysis, transcriptome data from different tissues, including siphon, gill, intestine, labial palp, mantle, and foot (muscle) were available at GEO with accession numbers GSM7511488 to GSM7511505. To quantify the abundance of putative *S. constricta* opsin genes, we employed FPKM (Fragments Per Kilobase of transcript per Million mapped reads) values. These values were detailed in Table S4 for temporal expression and Table S5 for spatial expression. The data were then visualized using GraphPad Prism 7 software, allowing for a comprehensive understanding of the expression patterns.

### Cloning of four representative putative *S. constricta opsins* with relatively higher expression

Based on the expression abundance data derived from the transcriptome analysis (Figs. 3 and 4), we cloned four putative *S. constricta opsins* with relatively higher expression levels for further functional investigations. These included *S. constricta opsin1*, *5*, *7*, and *12* (designed based on their recording order). Additionally, the rationale behind choosing these four specific opsins was guided by insights from the phylogenetic analysis (Fig. 2). Specifically, *Sc_opsin1* and *5* belonged to Gq-opsins, a well-recognized category responsible for primary photoreception in invertebrates (19, 45, 46). *Sc_opsin12* belonged to xenopsins, known to employ a signaling cascade similarly to c-opsins (5, 11, 37, 42). While *Sc_opsin7* belonged to Group 4 opsins, which are generally associated with photo-isomerase activity (16, 47, 48). It’s worth noting that another xenopsin, opsin13, did indeed exhibit relatively high expression in juvenile *S. constricta* (Fig. 3*G*). However, its expression was notably low in the tissues of adult *S. constricta*, especially in external tissues like the siphon (Fig. 4*A*) and foot (Fig. 4*F*), which are considered potential photosensitive tissues. These results strongly suggest a higher likelihood of phototransduction function for opsin12 compared to opsin13. Consequently, we chose to focus on opsin12 rather than opsin13. Furthermore, as Gq-opsins are widely recognized as functional opsins in phototransduction among invertebrates, we have included both of them in this study.

First, total RNA was extracted from fresh mixed tissues of adult *S. constricta*, including foot, siphon, and labial palp, using the Total RNA Kit II (Omega). The quality and concentration of the extracted RNA were evaluated using the NanoDrop One (Thermo). Secondly, a total of 1 μg RNA was reverse transcribed into cDNA using the HiScript III first Strand cDNA Synthesis Kit (Vazyme). Third, the open reading frames (ORFs) of the selected opsins were amplified using the synthesized cDNA as a template. Specific primers were designed using Premier 5 software and are listed in Table S7. The amplification was run on a PCR instrument (Eppendorf) using the MightyAmp DNA Polymerase Ver.3 (TakaRa). Fourth, the PCR products were separated and size-screened using 1% agarose gel electrophoresis and the bands of expected size were excised, purified, and subsequently cloned into the pMD 18-T vector (TaKaRa). The constructed vectors were transformed into competent *Escherichia coli* DH5α cells and plated on solid LB medium containing 100 μg/ml ampicillin. Finally, the positive colonies were identified, cultivated, and the plasmids were isolated. The cloned inserts were sequenced by Hangzhou Youkang Biotechnology Co, Ltd to verify their sequences.

### Analysis of expression response of four cloned putative *S. constricta opsins* to different light spectra

To unravel the photosensitive adaptability of the cloned putative *S. constricta opsins*, we conducted three independent biological experiments, each in triplicate. The average shell length (mean ± SD) of the juveniles in the three batches was 693.79 ± 10.05 μm, 701.23 ± 8.11 μm, and 705.07 ± 6.34 μm, respectively. These juveniles were obtained from Fujian Dalai Seedling Technology Co, Ltd and were acclimated under various light spectra conditions. Further details about the juvenile culture can be found in our previous publication (26). Briefly, the juveniles were initially acclimatized for 3 days within aquariums (dimensions: 20 × 20 × 20 cm) in a completely dark environment. These aquariums contained a layer of fresh sea mud (1 cm) and seawater (15 psu, practical salinity units) with a depth of 15 cm. The breeding density of the juveniles was maintained at 2 to 3 individuals per square centimeter. Following this period, the aquariums were exposed to various light spectra treatments for a week each. These treatments encompassed complete darkness, as well as white light (with a peak at 400–800 nm), red light (with a peak at 627 nm), yellow light (with a peak at 591 nm), green light (with a peak at 523 nm), cyan light (with a peak at 501 nm), blue light (with a peak at 463 nm), and violet light (with a peak at 397 nm) (Fig. S11). The LEDs emitting different light spectra were procured from Shenzhen Yamingjie Intelligent Technology Co, Ltd and were positioned on the top of the aquariums at a height of 20 cm above the water surface. The light intensity was consistently set at 10.665 ± 0.089 μmol/m^2^/s, as established from our previous findings (25). The photoperiod was set as 12 h light (8:00 AM–20:00 PM):12 h dark.

Throughout the entire acclimation period, the juveniles were fed twice a day (at 8 AM and 6 PM) with a mixture of microalgae, specifically *Isochrysis galbana* and *Chaetoceros calcitrans* (1:1, v/v), at a concentration of approximately 300 to 500 cells/μl. Prior to each feeding, half of the culture seawater was replaced. The experimental was carried out within an air-condition room, sustaining a temperature of 19 °C ± 0.5 deg. C. Continuous aeration was ensured to provide optimal conditions for the specimens. At the end of the experiment, the juvenile clams were subjected to a 1-day fasting period and subsequently collected within their respective light spectra environments. Notably, for the dark treatment, both the feeding and collection procedures were conducted under dim red light. The collected individuals were immediately frozen in liquid nitrogen and then stored at −80 °C for subsequent gene expression analysis.

The relative expression of the cloned putative *S. constricta opsins* were carried out by quantitative real time PCR (qPCR) using the specific primers in Table S7. In brief, total RNA was extracted from the collected samples following the procedure described earlier. Then, 1 μg RNA was reverse transcribed into cDNA using the PrimeScript RT Master Mix (Perfect Real Time, TaKaRa). The qPCR was conducted on a quantitative thermal cycler (Longgene Q2000A) utilizing the TB Green *Premix Ex Taq* II (TaKaRa). The procedure of qPCR consisted of an initial denaturation step at 95 °C for 30 s, followed by 40 cycles of denaturation at 95 °C for 5 s and annealing at 60 °C for 30 s. Subsequently, a melting curve analysis was performed, gradually increasing the temperature from 58 to 95 °C at a rate of 1.8 °C per minute. Finally, the relative mRNA expression of the target genes was normalized by the housekeeping gene *β-actin* using the 2^−ΔΔCT^ method (81).

### Analysis of phototransduction pathways driven by four cloned putative *S. constricta* opsins

To elucidate the phototransduction pathways driven by the four cloned putative *S. constricta* opsins, we employed a comprehensive approach. This involved the construction of luminescent reporter vectors for both Ca^2+^ and cAMP, alongside recombinant expression vectors for the opsins. Subsequently, luminescence signals driven by *S. constricta* opsins expressed in HEK293T cells were detected using a Varioskan Flash multifunction microplate reader (Thermo). These signals were measured following transient light irradiation. The detailed methods employed were outlined below.

### Construction of expression vectors

A luminescent calcium reporter (Aequorin) was engineered by employing the photoprotein aequorin derived from *Aequorea Victoria* (NCBI accession number: AEVAQ440X) (82). The construction procedure adhered to the methodology outlined in a previous publication (38). For a luminescent cAMP reporter (pGlosensor 22F), procurement was made from Promega (E2301). In the construction of the expression vectors, we initially inserted a six-base linker (GCTGCA) and a 24-base tag protein encoding the 1D4 epitope (ETSQVAPA) into the C-termini of the mammalian expression vector pcDNA3.1 (Invitrogen). Subsequently, the ORFs of the four cloned putative *S. constricta* opsins (excluding termination codons) were integrated into the pcDNA3.1 vector with 1D4 tag. This integration was achieved using primers that featured *Hind* III/*Sac* II restriction sites (Table S7). It’s important to note that the base sequences of *S. constricta* opsins were retained without undergoing humanization. Meanwhile, for the purpose of detecting the Gαq, Gαi, and Gαs-coupled phototransduction pathway, the *H. sapiens* melanopsin (Opn4), *H. sapiens* rhodopsin (RH1), and Jellyfish opsin (referred to as JellyOp), respectively, were chosen as positive controls (37, 38). Their sequences were directly synthesized by Hangzhou Youkang Biotechnology Co, Ltd.

Furthermore, to illustrate the critical functional aa in the phototransduction pathway of *S. constricta* opsins, we specifically targeted the K356 residue in the TM7 domain responsible for retinal binding (3), and the asparagine (N362) within the NPXXY motif, recognized for maintaining the structural integrity and stability of visual pigment (30), within Sc_opsin5. Mutations were introduced at those positions, with histidine (H) and K being employed as substitutions, respectively. The corresponding mutant sequences were directly synthesized by Hangzhou Youkang Biotechnology Co, Ltd.

When necessary, the recombinant plasmids were transformed into *E. coli* DH5α cells and sequenced as mentioned above. Ultimately, *E. coli* DH5α cells containing the recombinant plasmids with accurate sequences were further utilized to isolate the corresponding recombinant plasmids using the Endo-free Plasmid Mini Kit II (Omega).

### Heterologous expression in HEK293T cells detected by immunocytochemistry

To confirm the successful expression of *S. constricta* opsins, HEK293T cells (National Infrastructure of Cell Line Resource) transfected with the corresponding recombinant pcDNA3.1 plasmids were subjected to immunocytochemistry.

In brief, HEK293T cells were initially seeded in six-well cell culture plates (Jet Biofil) containing preloaded tissue-culture treated 24 mm coverslips (Solarbio). Cells were cultured in high glucose dulbecco's modified Eagle's medium (DMEM, Corning) supplemented with 10% fetal bovine serum (FBS, Corning) and 100 IU/ml penicillin-streptomycin (Solarbio), and maintained at 37 °C in a humidified incubator with 5% CO_2_. After reaching 80% confluent 24 h post-seeding, cells were transiently transfected with recombinant plasmids of *H. sapiens* Opn4 (the positive control), empty vector (no opsin, the negative control), or the corresponding *S. constricta* opsin at a concentration of 2500 ng/well, using Lipofectamine 3000 (Thermo) according to the manufacturer’s instructions. Each treatment was performed in triplicate. Subsequently, 10 h after transfection, the culture medium was replaced with DMEM supplemented with 10% FBS and 10 μM 9-cis retinal (Sigma), and cells were further incubated for an additional 30 h. Importantly, all subsequent steps were carried in a dark or dim red-light environment as needed.

Following incubation, cells were gently rinsed twice with 1×PBS and then fixed with 4% paraformaldehyde for 20 min. After fixation, cells were washed twice with 1×PBS, followed by permeabilization using 1×PBS containing 0.1% TritonX-100 (Beyotime) for 5 min. Next, cells were blocked with 1×PBS containing 5% BSA (Sigma) for 1 h at room temperature, followed by overnight incubation at 4 °C with the monoclonal 1D4 rhodopsin antibody (diluted 1:500 in PBS) (Thermo, MA1-722). Subsequently, cells were gently washed six times with 1×PBS and then incubated with goat anti-mouse Alexa 647 secondary antibody (diluted 1:500 in PBS) (Abcam, ab150115) for 1.5 h at room temperature. After another three washes with 1×PBS, cells were stained with the cell membrane probe 3,3′-dioctadecy loxacarbocyanine perchlorate (DiO, Beyotime) at 37 °C for 10 min. Following three washes with 1×PBS, cells were further incubated with 0.5 μg/ml 2-(4-Amidinophenyl)-6-indolecarbamidine dihydrochloride (DAPI, Beyotime) for 5 min at room temperature. Finally, cells were gently washed twice with 1×PBS, cover-slipped using Fluoromount-G (Beyotime), and then subjected to fluorescence detection. Fluorescence images were captured using a Zeiss laser scanning confocal microscope (LSM880, 294 Carl Zeiss) equipped with a × 63 objective lens.

### Luminescent second messenger assays

The luminescent second messenger assays were conducted following previously described methods (37, 38). Initially, HEK293T cells were seeded in 96-well white opaque cell culture plates (Biosharp) with each well containing 200 μl of DMEM and 10% FBS. Upon reaching 80% confluent, the cells were co-transfected with the relevant reporter and pcDNA3.1-1D4-opsin at a concentration of 100 ng/plasmid/well using Lipofectamine 3000. Each treatment was carried out in a minimum of three independent biological experiments, with each experiment including three technical replicates. Subsequently, after 10 h of transfection, the culture medium was substituted with DMEM containing 10% FBS and 10 μM 9-cis retinal (Sigma), and incubated for an additional 24 h. Notably, from this point onward, all procedures were conducted in a dark or a dim red-light environment as needed. The specific procedures for detecting different phototransduction pathways were described below.

For the Gαq-mediated Ca^2+^ increase signal pathway: The cells were co-transfected with Aequorin and pcDNA3.1-1D4-opsin plasmids. After the 24 h incubation, the culture medium was replaced with CO_2_-independent DMEM (Thermo) containing 10% FBS and 10 μM coelenterazine h (MCE, HY-D1024 for Figure 7, Figure 8, Figure 9, Figure 10, Figure 11, Figure 12; GlpBio, GC43292 for Figs. S5–S10), followed by an additional 2 h incubation. Subsequently, the cells were subjected to the luminescence measurement. After an initial 10 s equilibration period, a white LED (same with the one in Fig. S11) was used to flash the cells at approximately 20 μmol/m^2^/s for 5 s. Immediately after the flash, luminescence was recorded for 210 s with a cycle of 2 s. It’s important to note that during the recording well exposed to a flash of light, the other wells within the same plate were shielded from light to prevent exposure to the flash.

For the Gαs-mediated cAMP increase signal pathway: The cells were co-transfected with pGlosensor 22F and pcDNA3.1-1D4-opsin plasmids. After the 24 h incubation, the culture medium was replaced with the CO_2_-independent DMEM containing 10% FBS and 2% GloSensor cAMP reagent (Promega, E1290). Subsequently, the cells were incubated for an additional 2 h before luminescence measurement commenced. After a 10 min equilibration period, the cells in the plate were exposed to a 5 s flash of white LED light. Following the flash, luminescence reading was taken every minute for a duration of 40 min.

For the Gαi-mediated cAMP decrease signal pathway, the overall procedures mirrored those described for the Gαs-coupled cAMP increase signal pathway. However, there was an exception. Due to the challenge of measuring cAMP decrease from the baseline cAMP luminescence, the cells were treated with 2 μM forskolin (Sigma) to artificially elevate the cAMP levels before light exposure. The luminescence values in the test were subsequently normalized based on the maximum value induced by the forskolin treatment.

Moreover, to further confirm the specific Gα protein involved in the signal pathway, the cells were subjected to treatment either with or without 100 ng/ml pertussis toxin (Glpbio, GC17532) during the incubation with the retinal. This pertussis toxin selectively inactivates the Gαi-mediated signal pathway without affecting the Gαs or Gαq pathways.

Furthermore, to reveal the sensitivity of *S. constricta* opsins’ phototransduction to various light spectra, the cells were exposed to different light conditions. This involved flashing the cells with red, yellow, green, blue, and violet lights (consistent with those in Fig. S11) at approximately 20 μmol/m^2^/s, in addition to white light.

### Statistical analysis

Statistical analysis was conducted using SPSS 20 software. For the assessment of *Sc_opsin* expression under different light spectra, we employed one-way ANOVA, followed by Tukey’s test for post hoc analysis. In the case of repeated measure data, like cAMP levels, we utilized repeated measures ANOVA (83). Statistical significance was considered when *p* < 0.05.

## Data availability

All relevant data can be found in the main text and supporting information.

## Supporting information

This article contains supporting information.

## Conflict of interest

The authors declare that they have no conflict of interest.
