# Supplementary material for: Eyeless razor clam Sinonovacula constricta discriminates light spectra through opsins to guide Ca2+ and cAMP signaling pathways
Source: J Biol Chem. 2023 Dec 1;300(1):105527. doi: 10.1016/j.jbc.2023.105527 (PMC10788561; doi:10.1016/j.jbc.2023.105527)
Supplement: Supplemental Tables S1–S7 and Figures S1–S11 [file mmc1.docx]

**Supplementary Information**

**Supplementary Tables**

**Table S1.** Distribution and differentiation of putative opsins from five marine bivalves investigated in this study

| Opsin family type | *Sinonovacula constricta* | *Mercenaria mercenaria* | *Modiolus*  *philippinarum* | *Crassostrea gigas* | *Mizuhopecten yessoensis* |
| --- | --- | --- | --- | --- | --- |
| r-opsins (Gq-opsins) | 2 | 5 | 5 | 4 | 6 |
| Xenopsins | 17 | 10 | 5 | 6 | 6 |
| Go-opsins | 0 | 0 | 1 | 1 | 2 |
| Retinochroms | 2 | 2 | 0 | 1 | 1 |
| Peropsins | 1 | 1 | 0 | 1 | 0 |
| Neuropsins | 1 | 1 | 0 | 2 | 1 |
| Total amounts | 23 | 19 | 11 | 15 | 16 |

**Table S2.** Detailed amino acid sequences of putative *S. constricta* opsins and Gα proteins

| Gene name | Sequence |
| --- | --- |
| Sc_opsin1 | MNEDFAINNSAIQCLGGSVSQRTELTCSARMSHSYWINMTEVPQTVHDLSGVLGVMVLFCSLAGNLSVIFLVIRFRSLRSISNIFVINLTVADSVLSIGNIPMFIASSFNGNWLFGYTGCQIYAFIGSLSSFVSINTLAAMAVERALVLLKPLPVQNRVSKKYFCIAITVIWIYSAFWATVPFFGFGRYILEGTDTSCTFDFFTQSILNRVYVLSISIAHFVLPVGIIVCSYTLIYRAIVFHQRMFSDAQTTYGEASIPLRLRKNNHGIRYETKVAKASVIVVTVFCASWTPYATVALIGVFSKQPIITRLGAGIPCVLAKFSTVVNPFLYALLHPKFRKKLHTFYACNSSPNDNKQLGIQRGVLHYRSTKLDCSPDIPGKMTTSM* |
| Sc_opsin2 | MYTNETDVDHTTEDPYAGLSILDIKRLTYPRWAHTIAGLILLTIGFFGTLENILVLHTFARSKQIRSATNIFIMCLSISDLGMALLGNPLAWFNALHGRWVVGEFMCYWEGFVVYTLGLTSLYLLTAVSVDRYIVIAKPLKAAFITKRVAALSCFGCFLGGLLWSIFPFFGWSSYGLEAPGVFCGLHYEDQSVSNTTYVLAITFFCFFFPMGIMIYCYYNVYMTVKNANKNSVWDMKSRVARRNLKIEKKMFKSCMIMCAVYWGCWTPYSIVSFWQTFGDADSIPLVLTAVPALFAKSQIVWNPIIYVATNKQFRQAFYANLPCTGLRQALVKREEVKEQSTKESNDADDDKTENVSTITKAHAANAVAPASATEMTKIDC* |
| Sc_opsin3 | MSVFEATMSNLQLTTIEPSGLSPLDIKRLNYPRWAHTAAGVFLLVIGIVGTVENVLVLRTFAKQKQLRSPTNIFIMSLAISDLTMALLGHPLAWYNAFHGQWVLGEFLCTWEGFVVYTFGLTSLYIMTAISVDRYIVIAKPMKAAIITKRVATFSCFACVLGGLLWSLCPFFGWSSYGLELSGVFCGLHFGNKSSSNTSFIVTIFLTCFLIPMSIMMFCYYHVFMTVKNANKNSVWDMRSRVARRNLKVEKKLFKSCIIMCACFWICWSPYAIESFWQAFGDADSVPLVLSAVPSMFAKSQIVCNPIIYVATNRQFRSAFYETLPCKSLREKLKQLDKKDVSAQDSNNDDEKTETHSITQLEIATIARPKDILSRINKVAPIVLLSPNATSVTRVYE* |
| Sc_opsin4 | MDNYSSFNNVTMVDFRDERLSELGYQLIGALLVIISLSAVGGNALVLVLVWRFKALRTNTNFLITNLAVADLGVSILGFPMSAVASFANKWMVGAVGCHWYGFSGMLFGLANIFLLTVISVDRYVLTCKHNTFLRMTIRHYVLLATLAWVSSSIWAAIPLTGACSYSLDPSGMMCVVTWLKKSPCYTPFILSVFVFCFVVPTIVITFCYVNTWRFIRSVGDGGQQENIEWTHEKQVAKMCAVAISLFLMAWSPYATTCLWAALGRPETIPAYMTVIPPLFAKASSCFNPIVYAFLNKRFRLALRKMLGLTQGPREEEAVRLHHLVS* |
| Sc_opsin5 | MNGTTVGSFLGEVTTSLAELTTSLYDVTSGVNSNSFSDNGTTELYGADELSAGTTGIPTTTHIFDTYNVFVHPHWKQFPLVANGWHYFIGVYITIVGITGVCGNALVIYMFSRVKTLRTPSNMFLVNLAISDMTFSIVNGFPLLTFSAFNKRWLFGQAACEFYGLIGGIFGLMSINTMATIALDRYFAIARPLHVAKNMTRKRSFLMIVVVWIWSFVSCMPPIFGWGRYIPEGFQTSCTFDYLTRSENNRSYILFMYIFGFAVPLGFIIVCYAMILKAINKHENEMKKTAKKLNAEMRTNQEKQRMEIKVAKIAMSILVLYLLSWLPYATIALIAQFGDASFVTPFWSEFPVMAAKASAMHNPIVYALSHPKFREALNEHMPWLMCCCKPKKPFISTASSTYNNRNASKRSISSVTGAMSMDSEISNIEDPVDLRMKKIEDKQLHNDRMARYNEERSTQSEDIPAGRIIQDLTKALVELSARTANQTARPIYLPSNVLQTTAGLNQGPGDTATLKTPDDGVFVLDSSTLPMLAAYISKITKNTSSGAGNVNPGFDMPEEKSEKGDGNGAKPKQKLNIKPEVPSTELQSDEDQL* |
| Sc_opsin6 | MDTDQLMLTFNETNITMQPYVASLNYSQDDLVNTSTVPQSVYISFCVLLILMFILGLGQNALTLYIFLKDKCIRKSHNVFIAGLALSDIAMCLFGVWMVVVSAICQKWVFRRPGCIYYGLATTTLGLTQIALLAAIAMDRYIIIVQPSCLKPLKMKCAIYIVIFCYLFGFIWALFPVLGWSSYQLEGIGIRCAINWRSKKQSDLSYLLSLLICAWVVPLFAIVFSYFEILRRIHKGENHPVRKAIRRQRKNICDTRRAFFLSWTPYSVVTIIVLFGNTENVPVHVSVVPALFAKSSIIWNPIIYVARHREFRRACRRHLKFLKFIQADTFSSSAMTPTSYKTRDTQTRNDHNEFEMITHTTRSTSLSNGANEPVLKRTALTSFDIESPNSDEV* |
| Sc_opsin7 | MGSDSEEQILTDVPFANQGLGLGYALTGMLGIVGNVLVFRTFWRERRSWLHLQLSIANIITLTSVAMSAPSALKGEWLFTPGLCTWTGFQVFLGGYAEMTFTSLICIETFLAVCQHNRYSKFSSKHFAIVSVCVWAYSLLLASLPLLGYSRYKKDASGSSCGIDLRRYNDHNRYYVNLVLGLTSFVTLAGVICIVLTCIKKPALDTHSYEPLFNDKQITKVSLLLMLFMVVCSIPYGYRTVWFVLKKDSNWRFVAVEFSHLAVKFGCCIQPLAYFLSSPQFKGHAVATVTGVTYEKKNN* |
| Sc_opsin9 | MNNTSHQFVQCSDGNMCGNGYFYSVATIVLSLALLVGVIANGTIIHLYSKIQTLQSGTNTLIVAMAIADLCSLMLGIPAVIVAAYRRSWYGGPMLCTLYASLVTQFGLWSTYVLALIAVDRFIVITQPADTPRINKYRATILLCASFGFTLIFTLAPILGFGSYKKEGLGISCAPDWTSRSSVDVAYNLTVMVLFVLLPLTVIIFSYSGIVNKVRRHVLIMSLQRRPQGDLRTRELRVGVSVFLTTALFFLSWLPYTVLCVLRITGYDGTIPLTLQSAAAIIAKMGFVWNPFVYACRNAEFRYFNLLN* |
| Sc_opsin11 | MNVTLVVAEQLSTKRWTEILAGVWLVLVIMIGTPLNLIVLVVFAKSRHLITPFSILMITLFILDLTMCSLMAVLPMIAEFSGGWLNLMDFKICIIEATSVYFIGLSSMFILAGIAISRQMVVRRLTSPDTWTRAHSFITVAVSVGLAAVFTVAPLLGFGSYGLEIHQTSCGLSWQDSSQLQVNYIIIIAIFCYALPFLIMTACYLTIHRTIRMSVDVAPQIQWYNQQRQRRLVKLSLVIIGAFFVAWTPYAVVSFYQAFGRSDNMHPLLTKSCSLLAKSEAVFNPLLFIITMKDIRERVISIVPGLASLCHRPENDTRTDTIPMNARTTNQTRTEGLTMMGQSTCIL* |
| Sc_opsin12 | MSTWLIRDMYQSDLKPNWNLSNESKAFWYNSNLGQNYSLETISQTTYVSFSVLLTFIFILGLVQNSMTIYVFLKDRYIQKTHNVFIAALAISDVAMCIFGVWMVIVSSIFQKWVFGYSGCVYYGFTTTTLGLTQIFLLATIAVDRYLIIVKPPFFKPVQMRRAVLMVVLCYAFGLFWASLPALGWSSYQLEGIRLRCAINWRSKKESDMSYLLTLLICAWVVPFIVILFCYIEICRRIRREESCPIRKVIRRQQKAKNKHIQSRDKKVAITVLLMIGAFFLSWTPYSVVTFIVLFGKPEDIPVQVSMVPALFAKSSIIWNPIIYVARHKEFRMACIRQFTYLKVVQDDTNNSSPSPSVVSKCLPTMAKTETTTLVTFNTNSPTQQQYTVTACTTMLQDFIKLSHLETVELV* |
| Sc_opsin13 | MNAKWDEMNYEIDETNNQTNTSLGFMTDPQIGQALSSQKDVVISQATFITFSILLTVVFVLGLVQNSLTIFVFMRDKCIRKTHNVFIVALAVSDVAMCLLGVWMVVVASIFQHWIFGYNGCVYYGFTTTALGLTQIMLLATIALDRYLIIVKPPCLKPIEMKRAIYMVLFCYLFGVFWALLPALGWSSYQLEGIRLRCAINWRSKKSSDLSYLLTLLICAWVVPLCIILFSYFEICRRIRRDEKAEVRVRIRRQQNRHNQKGIKTRDKKVAITVLLMIGAFFLSWTPYSVVTLIVLFGNPEDVPVQVSMVPALFAKSSIIWNPIIYVARHREFRLACIRQLTYLRLWQDTQTLRTASSIAPRGSPTACTNETQALFAIR* |
| Sc_opsin14 | MSSTENTTLTDVFYENHTNETPIAPIVYESIGASVVLLFIVALVEHLLVLVVFRRTPRLHTLTNYWVMSLIICDTLIVLNAFPLLAASCFAKDFILGDMGCLWDGFIVTCLGCSSIYLLTGLSIQRYCIMIRDAFGKNTNKTKTIIYISTCFGLGSGWGVFPLFGYGTYVPEGIGVSCAPDWRSKKVTDRIYTGLMFVGVLFIPLLIMCFCYCSILVKIWKAKVNNTTWFLQRRVKRDIQVTFSVAVIIVSFLVSWTPYSVVNIAGTFFDVDTYAIHPMLVQLPCLMAKTACVWNPIVYVCANSEFRKAIRKYFRLFRKSPSKEHGGQIEPSRKSSLELQSINYSCNV* |
| Sc_opsin16 | MDVNMCCVYNSTTKWPAEKAIGSAVFRTIGFFISFLFAFAMTEMIIILLVYRRTPKLHTLTNIWIVALIACDLVIAVVALPMLFISSVSETLVFGDVGCQIDGFIVTSFGSISIYLLTGVSVQRYLTMTQNYGYPNKIKRVRVLTFMSLTFPFGTVWGALPLLGYGHFIPEGIGVSCAPDWKSPVYSSKVYIGLLFLFVFFIPLGIILFCYGSVLKKIWNSKINNQTPHARRYTKRSIRVTWAIVLATFSFLVAWTPYSVLNLAGTFFFFNAYNIHPMMLQLPCLVAKTACVWNPIIYVCVNGEFRKAVITYLRQICQRTDIEQSSFHSLQINDTNTTAV* |
| Sc_opsin17 | MAGPIHIFNSSSVGWVQPLINASEQSRDRALWSSSLMAGQYMAVISTLGIITNILALCMFSRHPHVLHAQMHLIQYVCVSHLLMALIAFPFITVSCIAGEWVFGDPGCQFYGFFMTTTAFTSVSLLAAIALQRLLVVVSMQQASKLAKLALNRRIGLLCLTIGLTLGVLPFVGIGTYDLEPGFMSCTPKWWGREKRDFVYIIIILICAFFLPVSIICASYIGISIKIRSTKRSQISSSVQTKYQLRLAVIALLLIAFFFVSWGPYAIVAMLQAFDDEFQLTAMTSTLPVMLAKSCVLWDPVIYTLSFRKFREALRSDICRVISCIKPCTSTRRGDYGIALNDEQLNTSVGNRNGATKQSHHLDLPLVISCTQVSLIKSSPV* |
| Sc_opsin18 | MGLDKNNESIYEAVNQSTWAPNITETLDRMTKTLSLIAGHYMICVCLIGTFGNVTVLWIFRRHPDALNSSIQLVKFLCTCDLLMALLAFPFIVVSCIYNDWIFGKIGCQMYGFFMTTTSLTCVAILTVIGLQRFLLIVVMNNTTSSKLSKTSVHKYLALLCFVFGLIFGILPFLGFGSYGLEPGFMSCAPNWWSKVNYDFAYMILLLISAFFIPVCLIVTSYIGILIKVRKSSVSRPVNSAHAKYQISLTITTFMLVCKYETYMSIRRRHYDVNVGNSQEHFLNAAVLC* |
| Sc_opsin19 | MNDIDAKDILSGCVYYGVTTTALGLTQISLLAAIAVDRYLIINQPPGVKRLKVKDAKYIILICLIFGTMWAIMPALGWNSYQLEGIQLRCAMNWRAETWSDRSYLLTVMVCAWLLPLGVIIFCYVMIYRTIRTKEQTTIRQNMRRNSAGPKRFRSRDKNVALMSLMFIGAFFLSWSPYSVVTILMLAGDAHLVPPQAFIVPALVAKSSVIWNPIVYTIKDEEFRRACMKHVKISVFVFKLINLKVPAAVNSTRLTMANPSPSWQSGTLKTNDESRL* |
| Sc_opsin20 | MDVRENVSIFYTTEDSNTTRNNDSVYVLDFLSKLEITLQNPPQTKGTSLAAFVSFSFFITLIFCVGLLTNMTALYVHVKERRLWTRTHHALVSGLNISDTILCLSGMWMTVSSSFAKSWLFGRAGCVYYGVTTTALGLTQISLLAAIAVDRYLIINQPPGVKRLKVKDAKYIILICLIFGTMWAIMPALGWNSYQLEGIQLRCAMNWRAETWSDRSYLLTVMVCAWLLPLGVIIFCYVMIYRTIRTKEQTTIRQNMRRNSAGPKRFRSRDKNVALMSLMFIGAFFLSWSPYSVVTILMLAGDAHLVPPQAFIVPALVAKSSVIWNPIVYTIKDEEFRRACMKHVKISVFVFKLINLKVPAAVNSTRMTMANPSPSWQSGTLKTNDESRL* |
| Sc_opsin21 | MFILGLGQNALTLYIFLKDKCIRKSHNVFIAGLALSDIAMCLFGVWMVVVSAICQKWVFRRPGCIYYGLATTTLGLTQIALLAAIAMDRYIIIVQPSCLKPLKMKCAIYIVIFCYLFGFIWALFPVLGWSSYQLEGIGIRCAINWRSKKQSDLSYLLSLLICAWVVPLFAIVFSYFEILRRIHKGENHPVRKAIRRQRKNICDTRRTSRDKQVAVTVMLMIGAFFLSWTPYSVVTIIVLFGNTENVPVHVSVVPALFAKSSIIWNPIIYVARHREFRRACRRHLKFLKFIQADTFSSSAMTPTSYKTRDTQTRNDHNEFEMITHTTRSTSLSNGANEPVLKRTALTSFDIESPNSDEV* |
| Sc_opsin22 | MILIIVGAVLAIVWCFGVVLNAGVIHTVRKCRKFHTPINILIVGTAVCDVIMLVFAFPLIIISCFCERWIFGQRLCHFYGFIVTLFSVSNICIITAVTIDRYVVINKIKLGFKFTTYKASLTVLSCVWYGLLWATSPLVGWGHFVVDRGGVSCEPDWSNRNGTIRIYNISILVMTFIVPVAIITFCYSKILRKVRLNRIQVPSTDHQQSMEEVRVGISSLLAIVLYLAAWVPYIALSFWALDQHPTQVPVWSALIAPLLAKSTCVWNPVMFYVTDKTFRKALGKLVAEAAIKLMKSHETTDTSTINTITFRQA* |
| Sc_opsin23 | MYEVFGILFTLITCAGVFLDSAIIHTIRKCKRLHTPNNVLIVGCILCDMIKLTIALPLMVVATFNMGWVFGRFWCDFYGYLVTLTGVTNICILTAVTLERYLILTKTTIRMKITTYTAGMTVLSCFWYGLLWATFPLVGWGTYVVDQAGISCAPDWGHNNPSHRSYNITIFVMVVSLPVTCIVFCYTKILLQMRENKVHPTNATTRNTYMDEVRVGLAALVAIVSYMLACIPYLAASVLTFVTKSHFVPTWVSLTIMCLSVSTSVWHPILFFTNNRTFMAVFRNVLMCRKPET* |
| Sc_opsin24 | MNNSSVHIETDPYFKDSEAGKFYSVTFGVAFVVLFGIGFVINSVVLHTFRKVRSLHTTTNVLIIGCVVCDMSMLIIGFPFNIVSAFCGNWIFGDAFCTCYGFICTVIGITNVGILTAISIERYLVICYLPFGIKLTLFQSYLSLMACFGHGLLWAILPLSGVGRFVLERNRASCGPDWTNTSPSVRSYNYAIFVVAFLLPVCIVGFCYLSIYKKVRSNRHITVHVSDGLSEISRDHQYQVQMNREANVALSASLALVTFLAAWSPYASMSLYSMYQSVDDIPPWVTFIAPLMAKSACVWNPLVYVIRNKVFHQAVSTMVCTPVEHLRQTTMQTIVQVIHR* |
| Sc_opsin25 | MLKLYETAMTDVPNNRTQDHENIYFINDEERFLSKLTHWEDLLVGAYLSIICISAVFLNTMVIYTCFKNWTSLVMSDFYILNLACSDVLLPLSSFPLSIFSSFKHTWSFKNIGCTMYGLSGFFFGLVSISTLTMMGITRYISICQPHIDVNKRGITRNIIIGTYLFSLVWSLIPLSGWGSYTAEAYGTSCTLQWDQNRSFITLMSVCCIVTPSVVMVLSYGLILLKCRRSNRNITTWQNTRNGKLSRKESYLIKITFAMCWAFLICWMPYAIVSMWTAYGDPRILPIRMTVVAVLLAKSSIVVNPVIYFLLSKKFRPMLSESFGVLWCKLSCLREAVEKLYRRGSHDQISEETRSKFDDNKAMSSNSASINMESLTLIKVKSLEKGLGDVLL* |
| Sc_opsin27 | MDTRTIENRMMLFAIYVFVGMLGIVGNVLVFRTFWRERRSWLHLQLSIANIITLTSVTMSAPSALKGEWLFTPGLCTWTGFQVFLGGYAEMTFTSLICIETFLAVCQHNRYSKFSSKHFSIVSVCVWAYSLLLASLPLLGYNRYKKDASGSSCGIDLRRYNDHNRYYVNLVLGLTSFVTLAGVICIVLTCLKKPALDTHSSEPLFNDKQITKVSLLLMVFMVVCSIPYGYRTVWFVLKKDSNWRFVGVEFSHLAVKFGCCIQPLAYFLSSPQFKGHAVATVTGVTYEKKNN* |
| Gαq | MACCLSAEQQEQKRINQEIEKQLRKDKRDARRELKLLLLGTGESGKSTFIKQMRIIHGTGYSEDDKRSFIRIVYQNIFMAMNAMIRAMDTLKISYKDPTNEENASKTCRIISLFTPLQENASMIRQIDHETVTNFEQQFVEAIKRLWADSGIQECYDRRREYQLTDSAKYYLDDVDRITQPDYLPSLQDILRVRSPTTGIIEYPFDLDQIIFRMVDVGGQRSERRKWIHCFENVTSIMFLVALSEYDQVLVESDNENRMEESKALFRTIITYPWFQNSSVILFLNKKDLLEEKIMHSHLVDYFPEFDGPKKDAQSAREFILRMFVDLNPDPDKIIYSHFTCATDTENIRFVFAAVKDTILQLNLKEYNLV* |
| Gαo | MGCTLSAEERSAMERSKAIEKGLKEDGIQAAKDIKLLLLGAGESGKSTIVKQMKIIHEGGFTSEDNKQYKPVVYSNTIQSLVAIIRAMGTLNIPFGSNERESDAKMVLDVIARMEDTEPFSEDLLAAMKRLWQDAGVQECFGRSNEYQLNDSAKYFLDDLDRLGGKDYMPTEQDILRTRVKTTGIVEVHFSFKNLNFKLFDVGGQRSERKKWIHCFEDVTAIIFCVAMSEYDQVLHEDETTNRMQESLKLFDSICNNKWFTDTSIILFLNKKDLFEEKIKKSSLTVCFPEYTGKTTYEEAAAYIQAQFEAKNKSSTKEIYCHQTCATDTNNIQFVFDAVTDVIIANNLRGCGLY* |
| Gαi | MGCAVSTEDKIAQERSKAIDKDLRADGEKAAREVKLLLLGAGESGKSTIVKQMKIIHEKGYTQEECLQYKPVVYSNAIQSMIAIIRAMGQLKVDFGHPDRSDDAKQFFSLAGNADEGDLSGDLAAIMKRLWKDSGVQHCVGRSREYQLNDSAEYYLNALDRISMPNYIPTEQDVLRTRVKTTGIVETHFTFKDLHFKMFDVGGQRSERKKWIHCFEGVTAIIFIVAMSEYDLTLAEDQEMNRMMESMKLFDSICNNKWFTDTSIILFLNKKDLFEEKIKKSELKVCFPEYTGSNTYEEAAAYIQLQFENLNKKRDTKEIYTHFTCATDTNNVQFVFDAVTDVIIKNNLKDCGLF* |
| Gαs1 | MGCFRRNSGDEEEKLRKETNKKIERQLQKDKQTYRATHRLLLLGAGESGKSTIVKQMRILHVNGFSPEEKKQKIEDIKRNVRDAILTITGAMSVLNPPVALEKPEHKFRVEYIQNKTVDPDFNYPPEFYEHTEILWQDKGVQSCFERSNEYQLIDCAQYFLDRVHIVKQADYSPTEQDILRCRVLTSGIFETKFVVDKVNFHMFDVGGQRDERRKWIQCFNDVTAIIFVTACSSYNLVLREDPSQNRLKESLELFKSIWNNRWLRTISIILFLNKQDLLAEKVKAGKSKIEDYFTDFKRYTTPSEVQIEPGDDPEVVRAKYFIRDEFLRISTASGDGRHYCYPHFTCAVDTENIRRVFNDCRDIIQRMHLRQYELL* |
| Gαs2 | MRETDMSCCCGPCLAGDFRYYEGPEEAKQARLRNKQINKMLSEQHRQDLKKLKLLLLGTGESGKSTITKQMKIIHINGFDTSERLAKIADIRRNIMESIVVIMCAMPQLGIELELPENKSRMEHVLQEASSAEVCITDELLEVVEHLWADNGVKQCYSRSYEYQLIDSAKYFLDKIHEVRSEDFTPSDQDILRCRVLTTGIQHIEFDVVDDGLPVQFSVFDVGGQRGERKKWIQVFDSVVAILFLADCSSYDQTLREDRTKNRFLEALEIFEQVWKNRFLKNVSVLLFMNKMDILAEKVQNGRSIKYFTDKHPGIFPDFDAFSPSNSERLEFLDSYQRPPDGEGKKRRGSRSKSADVNPELIKTAVYIKHIFMKISKGEIELKPTVQEITKDWHQNHRCEYFYTCAVDTNNIQRVLDGCRTLIIRKHLERFGII* |
| Gα12 | MADVFVCCVSDPEKAQRNRSKTIDKQIAKEKIQFRRTVKILLLGAGESGKSTFLKQMRIIHGEDFDDETIRDYRNTVYSNIIKGMRVLIDAREKLGIPWGDQANSKHAQFVFGYDNSKLDENVFQSYVESLEKLWNDAGILAAFDRRREFQLGDSLRYFMSSLKRIGAPNYKPVRLDILHARKATKGIIEHAFDIKGIPFLFVDVGGQRSQRQKWFQCFDGITSILFLASSSEFDQVLMEDRKTNRLVESCDIFEIIINNKTFVRVSIILFLNKTDLLEEKIKYVSIRDTFPEFEGDPHNLADVQNFMLGLFDDRRRERNKPLFHHFTTAIDTENIKFVFQAVKDTILQDNLKSLMLQ* |

**Table S3.** Detailed amino acid sequences of putative opsins from four other investigated marine bivalves

| Species | | Sequence name | Sequence |
| --- | --- | --- | --- |
| *Mercenaria mercenaria* | Mm_opsin1 | | MEGCVYYGFSTTLLGITQISLLATIALDRYLVIVRPTHNTIGMNKAVIMVLCCYGYGFLWAVLPAIGWSSYQLEGIRLRCAINWRSKRPIDLSYSMSILVLAWVVPLGVILFCYGGICTLIYQEREKPLRAMMRKGKSTKHRRREQRVAITVLLMIGAFFLSWTPYSIVTIIVIFGKLDDVPVSVIMIPTLMAKSSIIWNPLIYVVRHNEFRKACVRHVPCISWIKNAFILSPSTTNTTSNSV |
|  | Mm_opsin2 | | MNNTLATKTGNLTDDWTVNQGLSSAVCILLAVIMIGTLIIGTIGNTLVLALFIKYKSLRTASNIFIASLTIADLMMCLCGLPALINNYLLVKMEPNSAEFICYSDTFIGAFTGFGAIWSLMALAVDRCVVITRSMPVQHPTDKMIAYFVTATIWIVALAFAMMPFLGFGRYSIEGSKIACSVINFTKSFSNMLYNILIQVLYFFIPIICMICCYSIIFIKVRTHERQYFNVKQSGQVDDSSFRRMRKSRKLERNEMKTARAGLILISVFCLSWTPYSIVSWIGLLGNRYTLTPLAVALPAVFAKMSTILNPLLYALLLRSFKLKLRLLYKQHFQLTRNFNVASERTHVEFISNLDGRRREAADRTRRKNLSSL |
|  | Mm_opsin3 | | MYLNESEFFNTSDGPPAKLSVLEIKRLNFPKWGHQIFGTFLLFVGIFGFFENIMVLFTFYKSKQLRSPTNIFIIGLAISDLSMAALGNPLATTSGLNGSWFAGRFFCYWEGFVVYTFGLTEMYLLTAISMDRYVVIAKPLKSAMITKRVAVLGVVACFAFGVLWSIFPFFGWSSYGLEAAGIYCGLLWEDKSLSTTSYVVTISIFCFFLPFGTMIYCYYQIYMTVSTQIKSSLEG |
|  | Mm_opsin4 | | MEDRVVEYEFENQIVGFLDAVTSIFGLIGNALTFFTFWKERRRSVFHMQLAVANIVTLSSTFLSAPSALRGRWLFGDPLCQWFGFQVFIVGFAELALTCTICIETYTTVCRPDDEKTPSKTVLSIVSVVVWGYALIFSLAPLFGWNSYKHESLGISCGIDIENNTPSNYSYLVITHIFFVVMAVPGFVCLMLTLLRKPGDSKAYQPLLSDSELVRVTFLLFTFICLGCLPYYLRVVYFLRGFPPFFDSFLGVEFSHIAMKVCCLLQPIAYMIVSENFRKVAFSAITGVDPVKKNA |
|  | Mm_opsin5 | | MSSDIVIHSFYNRSDREVASLSENNGSTVNASHVVTDITLFQPISYYIIGSLMTIIMVIGFSTNLAILYTYATNRKIQTVDNTLIIGLALSDIGQAIFGIPFVVISSFSKHWIFGHTLCQYYAFITTSFGIAQIAMLTVIAMERYFVIVRHDKRLTNTPFRCMIAILGCFVYGSSWATGPLLGWSGYKEEEVGIACCVKWEVKDSSALSYTISLCTFGWFIPLFLIAFGYISIACSVSISTLLLHKFLYKSPVSSLCYCPIYDERNKRLYMRREVASSKSNRERKVAITVLIMIGAFIISWTPYSVVSLWSAVGDFRRIPLWVQVVPALFAKCSIIWNPVIYVARHENFRQGVKQSLGHLTICARGDRNQQNMNRINSYQQATRRQQACKALEKKHTTDQKKQSLSSENCVTDIESPVRSKSSSSVFMKKPASFKRARFKRHLTFLSTLDSSQHIVERSETEYLKIEKGVIYTASKCTQTEKETVRPYFRNNKQMIDKFVQTVSFSPLPHLSNMPESATDRYTTPVDYSVEVIHVENDADVKRPVDIVGQYETDVSGESNLTKDFEHASTSTRNSTYQKTKNLEALKRKRIELKRQLEIIYYDKHQNDRLPTQRQNTQPLSFTPSYLYSNQHEPLVLVENRLGQRPSDTIKNIPLRQNFYMPSKHSKSIPKEYLTPCDRLFSYNFQRRDDFRNKMKRERIISEPTDIEMKTYIYKPFTAGSQSIKDTTTDRELMMNTYFMNKKTMIMRQSTV |
|  | Mm_opsin6 | | SMVGISSVYFMLISLFCRYKSLRTSSNIFVINLTIADLMLCLLCLPMLINSLIRDDVDYEIKALFCNINTFLGAFTGFLSIWSLVCLAFDRCIVISRSLPVRYSSDKVVAYYVITAVWICCFVAAGIPFCGFGRYSIEGISTSCETTDDFTRSFSNMFYNIMIQVLFFVIPIVCITSCYLVIYTKVRKHEKLYFVVRKSGQYDEVSFRRMRRSRKLERTEMKTARAGIILISVFCFSWTPFSVVSWLGLYGNRELLTPIIIALPAVFAKILTILNPLLYALLLKSFKVKLSLCYIQHCSPLSSTYGSIERSIPLTGCRTSNLPRSNNCIVLAPRDNIVITPV |
|  | Mm_opsin7 | | MDFKDALLSIEINNTAYDVNTTTQNLQTKYASQLVAWEDTAVGAYLLLVCILAFILNALVIYTCLRNWSALIMSDFYILNLACSDVLLPISAFPLPIFSSFFHRWMFGDIGCVIYGFLGFFFGVVSITTLTIMGFTRYISICHPSIDLSGQKFRRCTLILPYVYALVWSSLPLSGWGSYSLESYGTSCTIQWNENRAFITMMSLFCIFTPSVIIFFAYSLILLKCRTSHRNVREWQRRSRKMSRKEFFLIKITFAMCWGFLLSWMPYAVVSMWTAYGDVTMLPIRFTATAVLLAKSSTVVNPVIYFLMSKKFRPLLLRSLKLPDLGIQASSLKSWKVISELLPVRTSSGTGSTNTASESSFTGHKFLVKVKQVHMDVGSDVQL |
|  | Mm_opsin8 | | MDVDDCYMKVKLIISVLFLLSAIASAIGNSLVLILVWRFKTLRNKTTILIINLAIADLGISVFGFPMSASSNFAHGWQMGQVGCQWYGFTGMLFGIGNIGLLTAISLDRYLLTCRHSVFLQLSVYHYIFLVLIVWGNALFWAMTPLLGWCRYALDPSGTACMVTWTDDTRMYTSYVMSVFVSSFLIPSVIIVFCYVNTWRFIRSVGDGGRQENVEWTHEKQVAKMCAIAVSLFLLAWTPYAVVCIWAAIGNPRTIPPLLLVAPTVFAKSSACYNPVLYALVNKRFRLAMRKLVGLGKTRRDKEEMRLHHFEG |
|  | Mm_opsin9 | | MGITDADLYNANGNGLAGINDIPDATRIVLAILFFCIAVVGLAANSAIIHTFRKSSTLQTPSNVLIVGCVMCDITMILIGYPFVIASHFHGNWLFGAAWCQGYGFVVTLLGVSSICILTAVAIDRYFVITESPFAKKITTFKAAMTILSCLGYGLLWATFPLVGWGRFVVEPGGMSCGPDWANHASSPQSYAIAILVLVLLLPVTIIVFCYLRIFVTVQKKSGQGDEFAARTRSTEVNVAFSSFLIITSFLVAWAPYGVMSVWVMLNDVNQLPQSVAMFAPLLAKSASTWNPLVYVGRSKQFRQAFVKIIFRRICTNRVNVTSVSVEPTQGPSSSSGV |
|  | Mm_opsin10 | | MNDSFETTAFDVISTLSAATSDADTTWSTSAASSGSVTSAVTATATALTTNIVETTTHLFDTYTVFRHPHWRNFPLVSDEWHYLIGVCITIVGFTGIFGNSLVIWMFTREKSLRTSSNMFIVNLAISDLTFSIVNGFPLFSISAFNKKWIFGNAACEFYGLIGGIFGLMSINTMVAIAFDRYQAIARPLHVAKFMTRKRAFLMIVVVWIWSFVSATPPIFGWGRYITEGFGTSCTFDYLTRTDNNRSFIFFLYIFGFAVPLGIIFVCYAMIIRAVKLHEIEMKKTAKKLNAEMRTNQDKKXMEIKVAKIAMSILILYLLSWLPYATVALIGMFGDASFVTPFWAEIPVLFAKASAMHNPLVYALSHPKFRTAIQNRLPWLACCCKPETQTPTPSGTYRDRNASKRSVSSVTGAASVSSEMSEIGESMYTDMEFRLRKLEEEQSAKRDPRVMSKRKGNKDKVADQVVEHADDIPAGRIIQDLAHALVEVTGT |
|  | Mm_opsin11 | | MNSSNTSDDNSNLNVSSLVYNVIGILMSVLFVLALFENILVLMVYQANKKLQSKTNLWIIAVIVCDLFIVLNAFPFVIIASFAQEYIFGTSGCKWDGFVVTLLGTSSIFLLTGLSLHRYLIMMYRHXKTRTYKRSNIILCIVMCFVLGLFWGITPLIGWGSFSLEGIKISCAPDWRSQEVKDLTYTIAMYICVLFIPLLIMAYCYIRILFKLLVGSSNKARNMAGYKREEGEEEIQGLRPAGVTMVKWKPKAPTGAIPSTFQHRTQV |
|  | Mm_opsin12 | | MKSRVARRNLKIERKMLKSCFLMCGVYWVCWSPYAVMSFWQSFGDPDTIPLVLTEIPAAAAKSQVVWNPIIYVATNKQFRKAFYASLPCVEWREKLIKREDVREPSSKESDFDDKTAKNDGSQTQANATSLPTAVPKAPGVSQTKDCATTSHMDELATVSSAVPSFNMGSLNKVAPAPDTVATLTTTQIVASKTGPTEVDC |
|  | Mm_opsin13 | | MAFAIVSAKVNLMSFISVCNIDASVAALTGFVSIWSLVALAFDRCIVIRRCMPGTHPADKSVTCFILTAIWLFCISCSAMPFMGIGRYVLEGSNMSCTFDYFTRSSENILYNVFIQIAFFGIPIICIIFCYVSIYLNVKKHERRYFSVRGNGNLHQESLRRMSRSRKLERNELKTAKAGIVLITVFCFSWMPYSLVSLIALFGNSSWISPIVVTIPVIFAKVSTVLNPVLYALVHKRFKSKLILFVRRYFSITHTTGSSDGHEMEENVARSTRRSFV |
|  | Mm_opsin14 | | MTNTTDVVFEVNSAFQTVSTACGFISLLVFSCSFVLSTSVLWALWKTKQEQVPVYRIVAFLLMDEVLVAFLLVDEVLVRIFGSPMIVTSMLASWWLYGDFGCIFYGFLMTWLGVTSTSLFTCISFERYIIMCRPDIKRLLTKNVVNVLGLACYLHGLVWGFLPLVGVNRYVYEPSGISCTPDWSHDNIGYMLGLFSVCFLMPVLVSIGCYSAIFRELRVRRRERVTLHHDASVERHVAITVAITMFAFLFAWTPYSVIGLNKTLGFEEQFPISKEITLIPLITAKTAGFWNPVIFGFRNKDVNKILAGKIRRIKELLSFQIVCRRTNQVIPEAGSNIKSSITCTDNGSQSNQVSIFQTGSTSGMKPQPREPTTVPLASIEI |
|  | Mm_opsin15 | | MSKPGLKVSGFASTTAMFTPRWIEILSASWLCFILVGGTLLNGGTLFIFMKNKHLVTTNNVYVIALIINNLLICILATGMPIVAEIGGHWLQLMSMKSCIFEAFIVYLFGLASMFVLVAIAVSRFIAITRPFLAARISKRQAVFAVCICELFALCFAVAPLLGWGSYGLEAHGTSCGLDWRDRSDGFLSYLIVITAVCFIIPMATMMFSYIRIYKTVKQAGENSFMMHRHSIQTRKEMLLLKLSIAFVSAFLISWTPYTIVSFYIAYARPDHLDPLLSRSAALVAKSQVLWNPVLYIIIVDGFRRKIFDMLPCKKRNEPTTLATIAISNLVEYTDRNEQTSALRQKNHKINQNKHINTTEKVKENISMAEGQVKNPRIQEMATSKTENIQNVATEASCIPYEEGLSNIPKTARDKTVPLDSVEGKSKTFESLKGTSNEHMPYNAEGISPYALKGTSNITFVKSTSMKNVREKKNNICCNPDQLITKEIRIDHKTTVGSSSPLHSPVNRTAIVNLISEDKDLIPIDSL |
|  | Mm_opsin16 | | MINTNVLAMLATNNSSDIESQLEQHQSPLSRPVCIILGFVLILAFIIGTIGNITVLALFVRYKSLRSVSNIFVINLTIADLMMCLCLPVLINDYFRGHAENDIVCKINTFLGAFTGFLSIWSLVCLAFDRCIVISRSLPVRHSSDKVIATYIIISIWFCCLLAAAVPFFGYGRYVLEGQTTSCEKKDNFTVSFSNMFYNIMIQILFFLFLYAVLQVVIFVYSLQ |
|  | Mm_opsin17 | | MASPAGGDFSMAEHRVVGVLYLVSGFLGILGNILIIAILRGENAITGCRSKIHVQFAIANILIVAGIPLSGSSAFAGSWLFGDIGCQMYGVLTYTGGMGAVTFAGLLCIQRYYSISLLNIYDMSGRSLFLSVLGWIYSVGIAIGPVLGWNSYVIESSGTACGLNWHKDDQSHRSLFITLPVVSIVMFLISLWSLRTAFSRPTPEKIDEKDWFTNNQLNWIAMANLIIVTTGLAPYGFFFALVSQGN |
|  | Mm_opsin18 | | MSDLEIRMLTYPKWGHQLVGLYLMFIGIFGFFENSLVLLTFYKSKQLRSPTNIFILGLAIGDFSMVIFGNPLAFTSALNGGWFAGDFMCTWEGFTVYFIGSAQLYLMMAIAIDRYIVIAKPLKSSMITKRVAALCVVACFGGGLFWAVTPLLGWSSYGLEASGCFCGLHYDDSSKTYIIFMFLFDFLIPMILMIYCYFKVYMTVSNSFTSINIIHIYICKIHTLRMFI |
|  | Mm_opsin19 | | MFSNISYLANSSSTDVGMSDLEIQRLNYPKWGHQIVGLFLMCVGILGFIENSLVLFTFYKSKQLLSPTNVFILGLAIGDFSMVIFGNPLAFTSALNGGWFAGDFMCTWEGFIVYFIGCSQVYLMMAIAIDRYIVIAKPLKSSMITKRVAALCVAACFGGGLFWALMPFAGWSAYGLEASGSFCGLHYEDSSKSYIMLIFIFDFFIPLLVMIYSYFKVFMTVSTFIGISNTCINIKCHPKYSYCLKV |
| *Crassostrea gigas* | Cg_opsin1 | | MDLLNITMNEVEVSKYPIPIPLYYVIGSGLLIVVTLGPFMNITSLAVFAQNKHLRSPTNIFVISLLLGDVGMSCVALISMVAHFNRYYFWGDRVCVFEGFWLYLMGLTNLYTHAVIAVDRYIVIAKPLSAHRVTKRVAVVAVLVVWIQGLLWASFPHFGWGKYTYEPARTSCAVEWDSKEIGSASYNVAITIWSLCIPLGLIVFSYYRVFMTIRHVARSGIWDTSSRIARKNLKMEKKMFKTIAYMLASYLWSWTPYTVVSVWAIIGESRDIPVYIITIPAVVAKSSCIWDPLIYLWTNRQFRIAFYKTMPCKSLGEKLLQRDELKHRETESSPPQEPQELRHKSNRLTPLRQTTTKQGPAVSVSEYGQKTETMGVSQINVPHVVVQGPSKTGNVC |
|  | Cg_opsin2 | | MNSSNLTEKVNVMSKEIPDSVHYIFGVVYMLLNVVATTGNVVVLYVFFRSKKLHSAIYFMISALCLGDLLMSSVGLTILSIASFATYWVLGDWGCIAYGTLMTFLGLMQITLLTVIAVTRYIFVVHNNNIGPCSAKVITLLCTLYALGFSVAPLLGWSKFVLEPIGTSCGPNWVGIEWSDVSFNMTLFFLCFLAPLSFILFSYIMIFWKVRRVASLHTNLYFPKPIKQSINCFLQIKKKRIQKKNETYEIHVSTTILFMIVAFLGSWTPYAFMALYIVITKNNQIHPLLAALPLVFAKCAPIWNPYIYFMRDTKFNHECRKMLPILSRLGLWRVNPGSSQADHVMHLNDTLSRKTSTYV |
|  | Cg_opsin3 | | MNTSNISGVVPGVPLVKYFIPQPLYYIIGTGLLFVFTFGSFMNFTGLLVFAKNKHMRSPTNTFIISLLMGDFGMSICSFISMTAHYNRFYLWGDNVCTFEGFWMYFMGLTNMYTIMGISFDRYIVIAKPLQASKITTRVAVAACLAIWFQGFAWAAFPFLGWGRYTYEAGRTSCSVQWDTDDIESASYNISIFIWSLFLPLMLIFYCYYNVFMTIRHVARNGVWDMNSRIARKNLRIEKKMFKTIVYMLVSYVGSWTPYSIVSLWAIFGEAKDIPPYLMTVPAVIAKSACIWDPLIYVGTNRQFRMAFYNTLPCDGLGKMLIKREEQKDKEADASDDEDEAGKKAEGTAVKKTTLVAPIDDEAGQTTQVENFPDPNSPQPGQSGNP |
|  | Cg_opsin4 | | MIVVMNGTNSSKFWTIAREHEKLADAMHYAIGVAFVLIMLISTLGNGGVMYVFIRTRNLRTPNYFLVSALCLGDFLMSTLGMPMFITSCFFTRWILGRVGCLVYGTLMTFLGLSQITLLASIAFTRYRYVVNNCSIDTLVAKIVVIMCYLYAFIFSLAPLFGWSRPVVEPIGTSCGPNWAGTEPRDVSYNLTIFVLCFFVPLSIITFSYFMIYLKIKRKRIHKKTDGYENHVTVTILLMIVAFLLSWSPYAALAMYVIITKNSTMHLALSSVPVVLAKTAPLWNPYIYFVRDRRFNKECEKLLPIFKKLGIFESHRTEQHEWHQMGDTSSTNVPLCEHEKQKTVADLKKARTF |
|  | Cg_opsin5 | | MSSTLHLNCLSNATEVNESICEETRQQGELPGPMYVVLAVYLFFLTFFGILVNGAVIYLYFSRREIATVSNMYIVALCLCGFLIATLGIPFAAASSIRHHWLFGDGMCKLHGFLLTGLGIVMIALMTGIAIDKYIHIVWFQAHRKVTKSFALGIITLCYVYGVIWGILPLFGWNKYILEPARLTCSVEWTGDFSNHSYAITILFTGLLIPVGVIASLYSSILKKIHLQRKSSQQLVSLARKQKMIKREKKVAITLFLMVGSFIVAWLPYSIYGFICILGYSKDIPLVWHTIPSVFAKASILWNPLIYASRSKVMKKALAETFPFLRWLIRNPDKVQTNEQKNQTSLTLLRSRTLNSSDVVVQSSSENPAISASV |
|  | Cg_opsin6 | | MDSTATYLPPTTVTDISMNETTTHIFDTYTFFVHPHWYNFPLVSDNWHYAIGVFISIVGIIGIFGNATVIYIFSTTKNLKTPSNMFIVNLALSDMIFSLVMGFPLLTISAFNKKWIWGNTACELYGLVGGIFGLMSITTLSAISVDRYYAIAHPLRAARNMTRKKAFIMICIVWVWSLCASLPPLFGWGRYVAEGFQTSCTFDYLTTTPNNRTYIFFLYLFGFAAPLLVIALSYILIIRALKKHERKMQQMAAKMKVDDIKANQEKTKAEVKVAKVAIIIVFFYMLSWSPYATVALIGQFGPAEWVTPFLSELPVMLAKASAMHNPIVYALSHPKFREALYKRAPWIFCCCEPPSKATPRTSKATNVSRTMSGLSDTVGGAASELSSCVSNLSDTRENTLEMRRTGKNSNDQETGNSSQLIQGLVQALVGVTNQQNRQVVYLPQNQSAQQAQGADQNNVFVVDNGGQKIDIKAYLQEIIAAEKIADAVKDTETNSVEMRNEPSTSQDEKEASKVESHHV |
|  | Cg_opsin7 | | MTENLSFLHPYWHRFGQISPTYHVTIGVIMVFTTVGAIAGNGLVIWCFISFRSLRTSSNVFIINLSIANLLMSLIDFPLLIIASFYHDWPFGQTVCEAYATLTGLSGLVTINSLTVIAVDRYCAIVIRVKSVSPAPKWKTYKATIVIWTYALFWSLSPVLGWSKYQLEGVRTTCSFDYLTRDATTISFIVAIATCEFAIPVTVIILAYCKIVTAMIVRRRNLSICKKSENSSLNFRLQRYRVRAEIRTAFIIIGLVCLFVVSWLPYTVTAMVGLFGDRTLITPYLSSVAGLIAKTSTVFNPIVYAIIHPKFKSKIKCMLYRRNSLNSGPLSKMNSRMSSNKFSSSGFEKQQIEFDIKSNRNSDLS |
|  | Cg_opsin8 | | METFGNSTEMLAQEKLSAASYISIGVYMIVLTLTAILGNCLVLFVYWKRSLYKRPVNWFILNISVADLCVALFAHPLSASASFNRSWNLDGVGCQMYGFFCYIFACNNIMTYAAISYFRYQIVCENNYVARIQRGRILSVLISIWMFSLFWTVSPLVGWNGYELEPYRLTCSIRWYGHVDSDRAYICLVLLCVYVFPLSVMIFSYIQIARHARRLSCTYPSSNEGGNKAKFLYNLERGATKISLLMTLSFVFTWTPYAFMSTVAASGVTINSPVVLLPTLFAKSSCAYNPFVFFFSHSAFKSYHFGFSSCIKTPETQEHPGVYVSNVRFRNRVGPSGATHATSRAGIRSSTEAIPNGDHSKVIQISVHPVQQSNVL |
|  | Cg_opsin9 | | MDYSNTTCLNVTETIVLHPPHQPKLSTLQGNIIAIYLTVVGIFSLIGNGITIVVICRFPKLRTAANIFIANLAAADLAITILTFPFSVVSHFYNGWQFGQDVCRWYGFNCMLFGLGSIAFLAAISCDRYLVTCRHELYCKFSKRHYFIVSVLLWTNCMIWAAAPFMGWGCYDNDVTGVLCTITWTCNGRAAFASFIYALSVVCFILPCCIITVSYRKTYQFIKAVGAGGSQENIEWTHQKQITKMCVVAVILFLVAWMPFTIYFLIISIQEASTLPALFHVVPAVFAKTSTCYNPVIYAIVNKRFRIAIQKTILCEKNVNDLDCMPMRRVR |
|  | Cg_opsin10 | | MAAVTGEFSEVEHKGVAILYFIFGVSGVLANSFVLKTFMKEGVLVSPKNILHINLAFSNILVVLGFPFSGLSSWHGKWVFGIRGCQLYGVESYTGGMACPAFIFTLCLERYLANRQRHIYDTMTTGTWWLIALAVWLHAITWAILPILGWNSYSMEASGVSCGLSWLKHDFNHASFIMVMTIEYATLFLLAILFLRSAKSYVDSPTVLEVNTKNWFTEKQLVWITFAFLMIAGVGWGPYGYFGIWSQRTKITSVSMLAVTLPPLFAKASASLYIVPYLAASDHFREAIVGGATPVKKDQ |
|  | Cg_opsin11 | | MMENIIHTDVNFTSCELHKLIARHWLNYEGVSHVFHLILGVVVVIVGIISFTSNGFILWMFYRYRSLRISSNLLVLNLAITDTFLALGNLPWLAISSFRGVWIFGYIGCQVYGFVGAMSGFISINTLAMIAIERFFVIVIREPYRHIRTSNKTVLISIMFIWIYSFVWAICPLIGWGSIILEGSMTSCTFDFFSRDVNTKSYVASILVFCFTVQLFLIICSYVRIYLKVLQHEKEIVNCYSDGNNTLQIQNRKVRKFRSVHVKTAKISLVIISIFCLSWTPYAVVAIIGNFGDASVITPLASTIPGVFAKLSTVLNPMIYALLHPKFRNKLPFRKKKVLKAKSNFQALMKLDNESPPQTSSSLSIQFNANTPKEFHKETVV |
|  | Cg_opsin12 | | MASILFLGCRIYGCLGFFCGVVSISTLALMSFSRYIHVCKSSKSPFFSKHTNFFIIGSYVYACAWASFPMLGWGEYGVEAYGTSCTLKWTENRGFVTLMLISCIIFPVIIMKFCYGGVYLYLRRHCKAFRTDRSKMGINVRKREGYLIKMAFMMCCAFMLTWTPYAVVSFWAAYGDPNSIPVRLTLVSVLIAKTSTIWNPLIYFVLNKKFRPHIRFCLQNLFRNETNAQDRRTRSSWFFCNSRSSMTSSL |
|  | Cg_opsin13 | | MNSSNLTEKVNVMSKEIPDSVHYIFGVIYMLLNVVATTGNVVVLYVFFRSKKLHSAIYFMISALCLGDLLMSSVGLTILSIASFATYWVLGDWGCIAYGTLMTFLGLMQITLLTVIAVTRYIFVVHNNNIGPCSAKVITLLCTLYALGFSVAPLLGWSKFVLEPIGTSCGPNWVGIEWSDVSFNMTLFFLCFLAPLSFILFSYIMIFWKIKKKRIQKKNETYEIHVSTTILFMIVAFLGSWTPYAFMALYIVITKNNQIHPLLAALPLVFAKCAPIWNPYIYFMRDTKFNHECRKMLPILSRLGLWRVNPGSSQEDHVMHLNDTLSRKTSTYV |
|  | Cg_opsin14 | | MTFRQYMYVTNSDLMRAESTHAPFFSKHTNFFIIGSYVYACAWASFPMLGWGEYGVEAYGTSCTLKWTENRGFVTLMLISCIIFPVIIMKFCYGGVYLYLRRHCKAFRTDRSKMGINVRKREGYLIKMAFMMCCAFMLTWTPYAVVSFWAAYGDPSSIPVRLTLVSVLIAKTSTIWNPLIYFVLNKKFRPHIRFCLQNLFRNETNAQDRRTRSSWFFCNSRSSMTSSL |
|  | Cg_opsin15 | | MMENIIHTDVNFTSCELHKLIARHWLNYEGVSHVFHLILGVVVVIVGIISFTSNGFILWMFYRYRSLRISSNLLVLNLAITDTFLALGNLPWLAISSFRGVWIFGYIGCQVYGFVGAMSGFISINTLAMIAIERFFVIVIREPYRHIRTSNKTVLISIMFIWIYSFVWAICPLIGWGSIILEGSMTSCTFDFFSRDVNTKSYVASILVFCFTVQLFLIICSYVRIYLKVLQHEKEIVNCYSDGNNTLQIQNRKVRKFRSVHVKTAKISLVIISIFCLSWTPYAVVAIIGNFGDASVITPLASTIPGVFAKLSTVLNPMIYALLHPKFRNKLPFRKKKVLKAKSNFQALMKLDNESPPQTSSSLSIQFNANTPKEFHKETVV |
| *Modiolus philippinarum* | Mp_opsin1 | | MYYNDSITLIDYYKTVAAETPVWFHISIGVILLLGCVGSIFTNGLVLLVFAKDTKLLTRINSYIAIICFLAVIMAVFGVPMVLVSCFQYFWFWGDIGCQYYAFLMSFCGLATMLLLTAVSVDRYIFVVRNNLSARLPKAFKTAMICGCLGVALGFALCPLLGWNQFIYEGVGTSCAIDLIGEEKNGRSFVITLLVMFFFVPVSVMAFTYGSVFAKVLRESKAYMRSNENNGTLKKLNMEKELAVTILLIVGFFLLCWAPFACLLLWKIADPESDISAMVMTVPSMLTKVGGVFTPLVYVMKNHNFRKQALSIISCKKLNTAVHPQVNQPREIPDTTLSNRSKYEISAVTVKSTNS |
|  | Mp_opsin2 | | CQFYGFIGGLFGLMSIITLAAISLDRYYNIAEPLKAAQYMTRKKAFMMIVIVWMWALLWSVPPLFGWGKYLPEGFQTSCTFDYVSKEPHMRTYFIGLYVGGFVVPLMFIVVCYILIWKAIRKHDREMLKMAKKMKVEDIRANQEKTNAEVRIAKIAMMIVFLYLLSWSPYAIVALIAQFGNAELVNPYVAELPVMLAKAAAMHNPIVYAFSHPKFREALNKRVPWLMCCCDVKPTTSPSNSRARTTKRTVSRQVSNDSYYAGGNDSDVSSCISHIDDYGQSIEMRKTVSETSFTRQQRRGAATESAIADAGSGDVVRELIQALVSVTNRQPTASPHYHPNIPPQNNPQNPSNDVFVIDNGQKVNISSYLAQLIAAGGPNMIASNLKDLVNKEALGNKDSVVDNVSVGNKDSPEGKDYKTKTKDIDVGKKQNDTEKAEKKTDNNATNLPDDKTQDKEEVSAASYTNDAYVKDSGDQSKAENTDDGLV |
|  | Mp_opsin3 | | MYYNDNITLIDYYKTVAAETPVWFHISIGVILLLGCVGSIFTNGLVLLVFAKEPKLLTRINSYIAIICFLAVTMAVFGVPMVLVSCFQYFWFWGDIGCQYYAFLMSFCGLATMLLLTAVSVDRYIFVVRNNLSSRLPKAYKTAMICGCLGVALGFAICPFLGWNQFVYEGVGTSCAIDLIGEEKNGRSFVITLLVIFFFVPVSVMAFTYGSVFAKVLRESKAYMRSNENNGILKKLNMEKELAVTILLIVGFFLLCWAPFACLLLWKIVNPESDISAMVMTVPSMLTKVGGVFTPLIYVMKNHNFRKQALSIIRCKKSNTAVHPQVNEPREIPDTAVSNRSKYEISAITVKSTNGTK |
|  | Mp_opsin4 | | MIFNYARFDNISTDATTSGFIDTTSVFDSYDYYIHSHWKQFGKIPDHWHYLVGIYITIVGITGIIGNSVVIWIFSTYVFIFFFLYFRLTSLTCVFYGFIGGLFGCMSIHTLSAISIDRYINIAYPLTSVVFMTKKKSFMMIVCVWIWSLVWAVPPIFGWGAYIPEGFQTSCTFDYLSREPHMRSYIFCLYIAGFALPLLIIVVCYVLILRAIRNHDKEMIIMAKKLKAEDIRANQEKTGAEVRIAKIAMGIILLFILSWCPYATVALIGQFGPKEWITPLVSELPVMLAKACAMYNPIVYAFSHPKFREALQKRVPCIRKCCKTTKMQNNSDRGSIPRNTIKRYVSRVTSADTNEPIGSLSDESSCISNIDDSLCAQPCQRNRAVSETIPNGDYNDIVLELVRALVGVASRQQFLQPVFMSNLCSPGENNQAFSDSNMSSYLSQLVSKNSHSGETHRNPSHAAETQLDESFNLLKDDSKTGLVESTVV |
|  | Mp_opsin5 | | MEEIYKSFRRSTYMFILNLAIGDTFLILANVPLLVISSFKGKWVGGLIGCHLFASAGGFAGFVSINTLTVLAVERSIVITCSLPYAKRLSKRGVFILCICLWAYSFLWSIPPYLGWGGHMMEGSRTSCTFDYFTRTTNNRSYVVSILVCCFGVQLIVIVISYAKIWMSVFRHQREIVNFYTKRHRSERMSFSLRVSSHHKRRNIEWRTAKTVLCLISVFTLSWMPYAIVAIIGQFGHQSYITPLSSALPGIFAKMSSFTNPIIYTLLHSKNRKVILSWIQRKDFISRSRANTSLDRLDTNKFLIHECSIESPHSIARHSNTTSM |
|  | Mp_opsin6 | | CQFYGFIGGLFGLMSIITLAAISLDRYYNIAEPLKAAQYMTRKKAFMMIVIVWMWALLWSVPPLFGWGKYLPEGFQTSCTFDYVSKEPHMRTYFIGLYVGGFVVPLMFIVVCYILIWKAIRKHDREMLKMAKKMKVEDIRANQEKTNAEVRIAKIAMMIVFLYLLSWSPYAIVALIAQFGNAELVNPYVAELPVMLAKAAAMHNPIVYAFSHPKFREALNKRVPWLMCCCDVKPTTSPSNSRARTTKRTVSRQVSNDSYYAGGNDSDVSSCISHIDDYGQSIEMRKTVSETSFTRQQRRGAATESAIADAGSGDVVRELIQALVSVTNRQPTASPHYHPNIPPQNNPQNPSNDVFVIDNGQKVNISSYLAQLIAAGGPNMIASNLKDLVNKEALGNKDSVVDNVSVGNKDSPEGKDYKTKTKDIDVGKKQNDTEKAEKKTDNNATNLPDDKTQDKEEVSAASYTNDAYVKDSGDQSKAENTDDGLV |
|  | Mp_opsin7 | | TLGNLDAALTTTISLSNGTTSVFDTYDYYIHPHWKQFGLIPDLWHYLVGIYITIVGITGIIGNSIVIWIFSTTRNLKTPSNMFIINLALSDLMFSAVNGFPLLTISAFNKKWMWGDSACQFYGFIGGLFGLMSINTLAAISFDRYLNIARPLSAAKNMTRKKAFMMIVGVWIWSLVWSVPPIFGWGAYIPEGFQTSCTFDYLSTEPHMRSFIFGMYIGNFAVPLAIIFVCYFFILKAIRDHEREMSKVVDKLKAEDLRSKQDKASMEIKVARIAMIIITLFIVSWSPYATVALIGQFGPSEWVTPVVAEIPVMLAKACAMHNPIVYAFNHPKFREALAVRAPWLMCCCDVKSTATSSRVSTNPNATKKPLSRNISGDSNYYGSEASSYVSNISDSVYDKNALGMSRQQSPGNSLATNEVFIELVRALVAVSNRGSVPESVVPANETDVIMNPENLAKILETFTNTKQAQENAKSETTISMPKISIATAEDKKKY |
|  | Mp_opsin8 | | MWNNTTESGEDLSPLVKTALGVYMATASILGTFANGICLYVFITNKSLRSPTNVFIIGLLLSDFTMCSVGAPFPAASMLANKWLFGWAGCVFHGFIVYLTGLSDMYILAAIAVDRYIVIAKPLQAANINYRVVTISVLLCYLFGLLWTCMPFFGWSSYAFEGAGLSCAISYEYSDPGAFSYNVAIFINCFILPIGVMAYSYYFVYMTIRSMARSTAFDKKSRVAKRNIRIEKKMAKTIAAMIGVFLFGWTPYAITAFYVAFGFGDLPLIVATTPSFFAKTASVWNPIVYVVSNKQFRRAFYELIPCSDLREKLKKKEEEKEAESEESDVDDKGKSISKTTQPPRRHGVEPIKEDDDAGEITIVEDMRGESTRPDDTEMKDLSDPNQGTSKA |
|  | Mp_opsin9 | | MYYNDNITLIDYYKTVAAETPVWFHISIGVILLLGCVGSIFTNGLVLLVFAKEPKLLTRINSYIAIICFLAVTMAVFGVPMVLVSCFQYFWFWGDIGCQYYAFLMSFCGLATMLLLTAVSVDRYIFVVRNNLSSRLPKAYKTAMICGCLGVALGFAICPFLGWNQFVYEGVGTSCAIDLIGEEKNGRSFVITLLVIFFFVPVSVMAFTYGSVFAKVLRESKAYMRSNENNGILKKLNMEKELAVTILLIVGFFLLCWAPFACLLLWKIVNPESDISAMVMTVPSMLTKVGGVFTPLIYVMKNHNFRKQALSIIRCKKSNTAVHPQVNEPREIPDTAVSNRSKYEISAITVKSTNGTK |
|  | Mp_opsin10 | | IVAITGNLLVFVVVYKERSLNRPINLFILNLAVSDFSVAVLGYPMTIISAAEGRWIFGDVGCKIYAFVCYTFSLNSLLTHVMISQYRYTVMCNYKTKKVATTRVYLTLVAGWVYGLFWTLSPLLGWSSYVEEPWSISCSINWTGTHITDVIYVYCVTVFVLIVPLSVMIISYIKIAKQSKRMKKSDDGQRTATAQPRYMLDIQTRVTS |
|  | Mp_opsin11 | | MKMYYNDSNGLTDYYRTLAAETPVWFHSSIGVMLLIGTLGSIFTNGLVLIVFAKDRKLLTRINTYIVILCSLAIIMAIFGVPMVLVSCFKYYWFWGEIGCRYYAFLISYCGFATMLILTAISVERYIFVVRNNLSARLPKAFKTLIICVCMGVPLVFALCPLLGWNQFVYEGVGTSCAIDLIGEENNGRSFVVTLLVVFFLIPVSVMTFSYGSLYAKVLRESKARIRSTFNNTTVDKLNMEKELSVTILLIT |
| *Mizuhopecten yessoensis* | My_opsin1 | | MNSSIFTNICYQCYFITSDYEVFRKDVLQATAIVMLFLALFGSLFNGLFIYILLKHNIVKTRNNIYVITLCVSSIGIAVLVVPTVGIASLEDKWMFGEVGCALHGFIMTTFGLIQIFVLAVMSFEKYIIVVRNNWDSFVSKNGTRFTIAGCLMLGVILGSCPLFGWNSYKLELQRTSCSIDWGDKSIKSLTYTYLLLLVGLVVPVSVMFFSYINIYLEIRRHIFNLHNVLRNSGKSYANMLKREVKVIKTMFILVCAFIFSWVPYSVMSIYAMFNDINSLDPLLYMLPTLFAKASVIWNPIIYLFINKSFKKALLDKIPILRKCCSCRLSKGRRELLSNETGPSPTYFKDGKSFDQQHTLRTSSQTQMAITIL |
|  | My_opsin2 | | MEVSTYVDGLVDNATTSSDALTSTVTSPGVVHIPSIGHDIIGGVLIVTMAVGICTNSLGIAIFIKDKTLRSPTNLFIAGLALCDLSMLVVATPLPTASSFAHRWLWGHTGCVFEGFMVYFLGLTSLYLLCAISVDRYIVIALPLKIALVTKRAATLTIVACYGLGFFWAMLPLVGWNSYQLEGLMTSCSVVWNTSNPKDYSYNVVIFFTCLIFPIGVMVYCYYHVYMTVRTVTRNNQWDTSSRIAKRNLEVERKISRTILVMIGVFIGSWTPYSIVSFWAAFGVASDIPVAVAGVPPYIAKTASVWNPIIYICTNKQFRRSFFNILPFANLQRESEEEQVESCEMMAAGSNEASVSKIQIRPLQPTDCHMTPQHDNPDVSVAHQARQSVDNCNIVHDDVMQENCVEQIHIPRQVVNEC |
|  | My_opsin3 | | MNFSSISKDLYACNCTSTDNEAFRRGMLQATAIFMLLVAIFGSLFNSMFIYILLEQSIRKSRTNIYVITLCVASILIAVFIVPSLGISSLADNWVFGEHGCILHGFAMTALGLFQIFILTVMSFEKYIIMAKKSWERLLSRTGTTVTIIGCSIIGLFLGSCPLIGWNSYKLEEQKTACAIDWSDRSPSALCYTVLLMFIGLVIPVSIMIFSYVNIFLVIRGHRHKLHQTLRSNGKCYENMLKREIKVIKTMFILVCAFVFSWLPYSAISLYAVFDDVTKVNPVLGMLPALFAKASVIWNPIIYMFINQSYKKVLKEKLSLYTPAACACCHCKCIRRGDNTATVTDTNNAVHGSFIIAYHE |
|  | My_opsin4 | | MPSPTESAVGMFSAWEFQCVGIAYFIFGAAGVLANAFTVVTFMRETPISSPRHILQLNMAVANLLVCAPFPFSGLSSFRGKWLFGDLGCQLYGTESFLGGMAATTFIPVVCVEHYLASCKKDFYDTLSSGTWWTVAMLCWMYAALWAILPLFGWNSYAIESSGVACGINWLKKDSNHMTYLQAMVITASILYVMAFYGLYQSRVHWDSVQVKSDPKSDANNWFTERQQAWICLAFMCIMFIGFGPYAILGLWAALTDSTTVSTLAIIIPSLACKASTSLYPIPYLVASDKFRAAYLGYRVTEHEAKTN |
|  | My_opsin5 | | MADNKSTLPGLPDINGTLNRSMTPNTGWEGPYDMSVHLHWTQFPPVTEEWHYIIGVYITIVGLLGIMGNTTVVYIFSNTKSLRSPSNLFVVNLAVSDLIFSAVNGFPLLTVSSFHQKWIFGSLFCQLYGFVGGVFGLMSINTLTAISIDRYIVITKPLQASQTMTRRKVHLMIVIVWVLSILLSIPPFFGWGAYIPEGFQTSCTFDYLTKTARTRTYIVVLYLFGFLIPLIIIGVCYVLIIRGVRRHDQKMLTITRSMKTEDARANNKRARSELRISKIAMTVTCLFIISWSPYAIIALIAQFGPAHWITPLVSELPMMLAKSSSMHNPVVYALSHPKFRKALYQRVPWLFCCCKPKEKADFRTSVCSKRSVTRTESVNSDVSSVISNLSDSTTTLGLTSEGATRANRETSFRRSVSIIKGDEDPCTHPDTFLLAYKEVEVGNLFDMTDDQNRRDSNLHSLYIPTRVQHRPTTQSLGTTPGGVYIVDNGQRVNGLTFNS |
|  | My_opsin6 | | MSLSSVLSASLVTGVVGNILVIWMFTTNKKLKTPSNMLIVNLALSDLTFSAVNGFPLKSISAFSKKWVFGMVACELYGLIGGIFGFMSISTLAAISIDRFICITKPLQAARIMTRKKAFIMIVVVWTWSVFWSIPPLFGFGAYIPEGFQTSCTFDYITKSTSNRIFIIGMYVFGFLMPCLIIIGCYIQILKAIKAHGKEMTRMADKLNAEEADKSKKAKAEMKIAKIALMLISLFILSWSPYATIALIAQFGSPDFVTPLMSELPVLLAKTSAMHNPLVYALSHPRFRAALAEKAPCFMVCCPPEKTPTSTLSRHVAGRTASQSSVTSCATNMSDCQGGIQMQPTPTTVNHGDLVKDLVGALVNMATQNANHVVQPVYLPNGVLHSNGTGIITVPQVNVISGQGHQNAAFVPEVETVDTGKVNTDKEKENDNVTSKDAVKV |
|  | My_opsin7 | | MVLGVTELATENTTHLYDTYDYFVHPHWKQFDPVPHTWHYFIGIFISIVGVSGVIGNIVVITMFSTTKTLKSPSNMLIVNLALSDLTFSAVNGFPLLTVSAFNTKWVFGDTACQFYGLIGGIFGLMSINTLAMISIDRCICITRPLQAMRIMTRKTSFIMIVVVWVWAVGWSLLPLFGLGAYIPEGFQTSCTFDYLTKTVSNRIYIIGMYVFAFALPLVLIIVSYIMIISSIRKHAREMANMADKMNAEEADKHEKTKAEIKITKIAMTLISLFILSWSPYATIALIAQFGDPSFVTPLMSEMPVMLAKSSAMHNPIVYALSHPKFRAALYQKAPCFLNCCKPSPKPPAEAPSRAQVGRTASDCSQSTIASSYGPGYEMQPTMNQGVNSGELVKELVGAIVSMANNQPPTVQPVFIPGTGGTVVTPIAATPAPAPTAEQVQTTTAAIIEKATKEPKV |
|  | My_opsin8 | | MSREAGTAILSVVEEVTTINTSASHPFDTYDYYIHPHWRQYPPVSDNWHYFIGLFITVVGISGVVGNIVVIWMFSSTKTLKSPSNMLITNLALSDLTFSAVNGFPLLTISAFNKRWVFGDAACEFYGLIGGIFGLMSINTLAMISIDRYICITKPLQAARLMTRKKAFFMIVIVWSWAVGWSLLPLFGLGAYIPEGFQTSCTFDYLTKTTLNRIYIIGMYLFAFALPLVIIIGCYIAILKAIRKHAKEMASMADKMNAEEADKKEKSKTEIKIAKIAMMLISLFILSWSPYATIALMAQFGDPSFVTPFMSELPVMLAKASAMHNPIVYALSHPKFREALMKKAPCLLSCCAPSEKPKKVNKAPVPQKPVMHRQLTHTFSDASMASVQTNISECFEMKTAKEQWIDSAEMVRQLVGVIVGMATKKSGDKPNALPQNGNVQMTEDGPSTETMQQPEEGVFTVNDGNLDLVAAAGALIDALGTECETEKPVDEGEVTTDGKDNPAFEPQEDDTQV |
|  | My_opsin9 | | MEVYNETSPDLMLGMDSNDINVSDYVYMLMALSMFAIFMMGLLFNALFIYILLFHNKLRTRNNVYIITLCVASILISVLAVPFVGVSAVSHHWLFGTNGCLFHGFIVTWLGLIQICILSVISVEKYLIIVRRNREDFLSSKATLTLVRRRRKIRFASCKDGTQTKAKEVKVIKTIFLLIAAFIVSWLPYSGLVFITMFYPYKSIHPVLATVPALFAKSSIIWNPVIYLMTNTTFKRAVVKLIPCAGVLILINSTPIFGAVTVIADVGNADSSEDD |
|  | My_opsin10 | | MNTQSPRESNNSVIVLFKNVTSVENVEEDFLNKSSASITEAEYAVLAFFMFSVSLAGILFNGIFVYVFLVHSKLKTRPNILLISLCISSFLIAALAIPFVGASAISGKWLFGRFGCVFHGFIVTALGLTQIAILTVLSFEKYISIVKYHWSHLVTQSATLLLLFGCFMYGFLLAAYPLLGWNRYTIEGANISCSIDWTARSPIDLSYSLCLLLIGLVFPLAVMSYVYISILVLIKRQRSIAQRYKGFQQHHRASRREVKVMKTIFLLVSAFLISWIPYSVYAMTSILGYADDVHPLIGTLPSVFAKASIIWNPLIYVCRNRSFKRALFDTFPSLLVLYRCTHRCRRRDSGEIASESTKMVHMKGPMSLSSQKSEINCEDNFDSCVQETGLDRNECCVSV |
|  | My_opsin11 | | MECFLNSEAKFRSMRKPSNVLLLNLSVTDLILSLSTLPVLGVASLRQAWVFGESGCLLYGFIGSVTSIVSITTLTMIAIERSIVITFSRKVTLKQLIMAIQFTWIYGIFWATMPLAGWGRYIIEGSRISCTFDFLSRDTLYKSFVISLQTCVFYVPVSLIMFSYASIFVKVSRNEREMDRLKNTQQLWRSSRFNIEIKVAKTALVITMVFCLSWLPYAVVALIGSFGNIQLITPLTSAIPGFCAKLSTSINPLIYVLLNGKYRSKLGKDFRSMLRCVCPST |
|  | My_opsin12 | | MAVGNLPAFAVASFNMRWVFSNIGCQIYGFVGSIASLTSITTLTLIAIERSIVIVHQLPWYQKTSVIWHEREIDQIALKTNSKIYFTNVSRERRKQKREFKTAKIAFGIILVFCFSWLPYAVVAMIGCFNGKLSLITPMAVSVSGLCAKIATAVNPILYALIHPKYRGKVRKDMKKGVSFVFKTVNNQDSRISHSNDSNEELQFSKFNRKESITNRPKEHTLLQKTDDFL |
|  | My_opsin13 | | MDIPCLCTGIPLYEDYYVNDSKLALNTSIDVAHIHKKDVMDLSIFYSKLSPVEDTLVALYLGVVGATSIFLNAMVLFVCFKKRRTLKSIDYYIVNLAITDLFLPLFGFPLVVTSSLQHEWQFGVYGCYVYGFLGFFCGTVSISTLAMMSFVRYMSVCEMQKSVHLTKHTSVLVFFTYVYACIWSIPPFLGWGNYGVEPHGTSCTLNWSGSRSFVTVMLIMCIILPVVIMTFCYGRVLLFLKRSTDNLNKWTPHRPNRKHANRKLERSLIKLTFTMCVAFVGTWTPYAVFSLWTAYGNKEEIPIRLTLSSILIAKLSTIINPTIYFVLNRKFRPFIKRYLSMPFHGILNLIGDKSSTSG |
|  | My_opsin14 | | MPFPLNRTDTALVISPSEFRIIGIFISICCIIGVLGNLLIIIVFAKRRSVRRPINFFVLNLAVSDLIVALLGYPMTAASAFSNRWIFDNIGCKIYAFLCFNSGVISIMTHAALSFCRYIIICQYGYRKKITQTTVLRTLFSIWSFAMFWTLSPLFGWSSYVIEVVPVSCSVNWYGHGLGDVSYTISVIVAVYVFPLSIIVFSYGMILQEKVCKDSRKNGIRAQQRYTPRFIQDIEQRVTFISFLMMAAFMVAWTPYAIMSALAIGSFNVENSFAALPTLFAKASCAYNPFIYAFTNANFRDTVVEIMAPWTTRRVGVSTLPWPQVTYYPRRRTSAVNTTDIEFPDDNIFIVNSSVNGPTVKREKIVQRNPINVRLGIKIEPRDSRAATENTFTADFSVI |
|  | My_opsin15 | | MQPASMTTDSMKGEMDSNTSDDVLGPLSGTTYLCIGIYQTLMGLSSILCNGFVIIVLLKGKPKYNVMHNILLLNMAITDLLISVIAYPLSASSSLNGSWVYSDETCVFYGFWVFCLAMGNMNTLAVIAICRYIVAVRPEYNYLLTKKNAKYFLLVIWTYAILWTGPPLVGWSTYTFEAYRTSCTINWGGRSLSDKTYNVTITFTCYLCHLIICCFCYYHVLKKYVTANNSNIGITLRSSSVKQQNTEESFNVEAVVSYHKVTTSRKVTTMCIAMLFSYLFAWTPYTVLSIWVMFVGDVEPWVHVIPTMMAKTSTLSNSIVYGILSSKFRESAKAMFKRNRNRVAPASITTHRNQTDSVATSSRSQIHPTAVTDHPRPWDKHPKGRCHTYNVHLIKNDVYIGKNSVTLPHEEQPF |
|  | My_opsin16 | | MEGNNTTNGSSLTLDPEEEYYIPDIYYNMFAVILFFTWILGSFFNGSALLVFFKNKNLRTATNMFVMALAVDDLAMSSICLFAASASYNKRWIHGDLICTIEGFLVYVLGLTDLYLLCAISLDRYIVIAKPLQANKINHGVAALSITACWMGGLFWSATPFFGWNYYRLEDSGVSCGVSFEPNDPSIQSYILSIFIFCFVGPFVLIMFSYYGVYMT |

**Table S4.** Specific FPKM values of putative *S. constricta* opsins expressed at various developmental stages

| Gene name | zygotes | | | trochophore larvae | | | veliger larvae | | | umbo larvae | | | creeping larvae | | | single pipe larvae | | | juvenile clams | | | |
| --- | --- | --- | --- | --- | --- | --- | --- | --- | --- | --- | --- | --- | --- | --- | --- | --- | --- | --- | --- | --- | --- | --- |
|  | 1 | 2 | 3 | 1 | 2 | 3 | 1 | 2 | 3 | 1 | 2 | 3 | 1 | 2 | 3 | 1 | 2 | 3 | | 1 | 2 | 3 |
| Sc_opsin1 | 0.069 | 0.000 | 0.000 | 1.223 | 1.744 | 0.853 | 2.618 | 1.630 | 2.049 | 1.344 | 1.984 | 1.538 | 1.772 | 1.786 | 1.220 | 0.912 | 1.013 | 0.747 | | 0.229 | 0.390 | 0.675 |
| Sc_opsin2 | 0.107 | 0.113 | 0.030 | 1.436 | 1.111 | 0.942 | 1.149 | 1.071 | 1.133 | 1.274 | 1.994 | 1.323 | 0.889 | 1.163 | 1.387 | 0.444 | 0.497 | 0.326 | | 0.044 | 0.188 | 0.111 |
| Sc_opsin3 | 0.000 | 0.000 | 0.000 | 0.665 | 1.033 | 0.820 | 0.546 | 0.810 | 0.600 | 0.308 | 0.385 | 0.509 | 0.293 | 0.367 | 0.394 | 0.072 | 0.183 | 0.124 | | 0.028 | 0.048 | 0.024 |
| Sc_opsin4 | 0.100 | 0.000 | 0.000 | 1.261 | 1.646 | 1.396 | 1.058 | 0.900 | 1.365 | 0.522 | 0.489 | 0.444 | 0.698 | 0.445 | 0.264 | 0.554 | 0.332 | 0.144 | | 0.331 | 0.141 | 0.139 |
| Sc_opsin5 | 0.000 | 0.000 | 0.000 | 0.037 | 0.085 | 0.144 | 0.033 | 0.061 | 0.100 | 8.212 | 8.270 | 7.852 | 3.603 | 4.835 | 2.317 | 1.611 | 1.370 | 1.639 | | 0.399 | 0.986 | 1.282 |
| Sc_opsin6 | 0.000 | 0.000 | 0.000 | 0.000 | 0.000 | 0.000 | 0.063 | 0.000 | 0.000 | 0.000 | 0.000 | 0.000 | 0.000 | 0.000 | 0.000 | 0.000 | 0.000 | 0.000 | | 0.000 | 0.000 | 0.000 |
| Sc_opsin7 | 0.163 | 0.289 | 0.368 | 18.642 | 16.009 | 16.149 | 17.349 | 14.289 | 15.098 | 5.608 | 5.878 | 5.320 | 2.195 | 2.010 | 1.895 | 1.793 | 1.666 | 1.448 | | 0.991 | 1.015 | 0.853 |
| Sc_opsin9 | 0.000 | 0.000 | 0.000 | 0.000 | 0.000 | 0.000 | 0.058 | 0.106 | 0.116 | 0.172 | 0.000 | 0.057 | 0.000 | 0.068 | 0.000 | 0.000 | 0.000 | 0.165 | | 0.000 | 0.000 | 0.000 |
| Sc_opsin11 | 0.000 | 0.000 | 0.212 | 0.553 | 0.637 | 1.080 | 0.781 | 0.650 | 0.285 | 0.561 | 0.656 | 0.417 | 0.219 | 0.835 | 0.166 | 0.065 | 0.125 | 0.000 | | 0.078 | 0.000 | 0.327 |
| Sc_opsin12 | 0.037 | 0.312 | 0.000 | 0.371 | 0.142 | 0.271 | 0.389 | 0.356 | 0.306 | 0.795 | 1.000 | 0.489 | 1.025 | 1.437 | 1.423 | 0.993 | 0.488 | 0.422 | | 0.365 | 0.490 | 0.256 |
| Sc_opsin13 | 0.000 | 0.000 | 0.000 | 0.258 | 0.255 | 0.684 | 0.596 | 0.182 | 0.399 | 0.523 | 0.337 | 0.162 | 0.850 | 0.702 | 0.772 | 0.486 | 0.525 | 0.063 | | 0.508 | 0.524 | 0.305 |
| Sc_opsin14 | 0.000 | 0.000 | 0.000 | 0.431 | 0.283 | 0.300 | 0.553 | 0.506 | 0.888 | 0.546 | 0.766 | 0.758 | 1.077 | 1.171 | 0.966 | 0.558 | 0.146 | 0.210 | | 0.121 | 0.154 | 0.305 |
| Sc_opsin16 | 0.000 | 0.000 | 0.000 | 0.000 | 0.000 | 0.287 | 0.066 | 0.121 | 0.199 | 0.000 | 0.061 | 0.000 | 0.000 | 0.155 | 0.154 | 0.061 | 0.000 | 0.000 | | 0.000 | 0.000 | 0.000 |
| Sc_opsin17 | 1.960 | 1.348 | 1.372 | 3.252 | 2.513 | 3.350 | 3.402 | 1.943 | 2.932 | 2.055 | 1.221 | 1.764 | 1.026 | 1.036 | 1.540 | 0.404 | 0.458 | 0.305 | | 0.131 | 0.074 | 0.111 |
| Sc_opsin18 | 0.000 | 0.000 | 0.000 | 0.121 | 0.070 | 0.297 | 0.164 | 0.300 | 0.164 | 2.371 | 1.413 | 1.390 | 1.456 | 1.734 | 1.591 | 1.301 | 1.729 | 1.505 | | 0.359 | 0.457 | 0.553 |
| Sc_opsin19 | 0.000 | 0.000 | 0.133 | 0.397 | 0.457 | 0.485 | 0.624 | 1.716 | 1.432 | 0.088 | 0.330 | 0.175 | 0.092 | 0.105 | 0.104 | 0.000 | 0.000 | 0.000 | | 0.000 | 0.000 | 0.000 |
| Sc_opsin20 | 0.000 | 0.000 | 0.111 | 0.623 | 0.956 | 0.365 | 0.597 | 0.342 | 0.449 | 0.147 | 0.000 | 0.000 | 0.038 | 0.000 | 0.087 | 0.000 | 0.033 | 0.000 | | 0.000 | 0.035 | 0.034 |
| Sc_opsin21 | 0.000 | 0.000 | 0.000 | 0.000 | 0.000 | 0.065 | 0.000 | 0.000 | 0.000 | 0.000 | 0.000 | 0.000 | 0.000 | 0.000 | 0.000 | 0.000 | 0.000 | 0.000 | | 0.000 | 0.000 | 0.000 |
| Sc_opsin22 | 0.171 | 0.290 | 0.424 | 1.467 | 1.358 | 1.180 | 2.197 | 1.350 | 2.101 | 0.561 | 0.980 | 0.683 | 0.424 | 0.547 | 0.362 | 0.190 | 0.227 | 0.270 | | 0.085 | 0.096 | 0.024 |
| Sc_opsin23 | 0.061 | 0.130 | 0.069 | 4.969 | 5.307 | 4.878 | 5.854 | 4.853 | 4.481 | 0.822 | 1.005 | 0.861 | 0.309 | 0.136 | 0.270 | 0.042 | 0.081 | 0.022 | | 0.025 | 0.129 | 0.085 |
| Sc_opsin24 | 0.136 | 0.000 | 0.000 | 0.459 | 0.264 | 0.728 | 0.309 | 0.614 | 0.673 | 0.051 | 0.191 | 0.050 | 0.212 | 0.182 | 0.120 | 0.142 | 0.045 | 0.196 | | 0.000 | 0.000 | 0.000 |
| Sc_opsin25 | 0.000 | 0.000 | 0.000 | 0.226 | 0.087 | 0.221 | 0.068 | 0.310 | 0.340 | 0.067 | 0.094 | 0.332 | 0.035 | 0.199 | 0.040 | 0.093 | 0.000 | 0.000 | | 0.037 | 0.032 | 0.187 |
| Sc_opsin27 | 0.448 | 0.238 | 0.378 | 2.260 | 2.494 | 1.656 | 0.762 | 2.403 | 1.953 | 1.671 | 0.548 | 0.497 | 0.521 | 0.896 | 0.592 | 0.233 | 0.149 | 0.966 | | 0.278 | 0.472 | 0.390 |

**Table S5.** Specific FPKM values of putative *S. constricta* opsins expressed in various tissues

| Gene name | gill | | | intestine | | | labial palp | | | foot | | | mantle | | | siphon | | |
| --- | --- | --- | --- | --- | --- | --- | --- | --- | --- | --- | --- | --- | --- | --- | --- | --- | --- | --- |
|  | 1 | 2 | 3 | 1 | 2 | 3 | 1 | 2 | 3 | 1 | 2 | 3 | 1 | 2 | 3 | 1 | 2 | 3 |
| Sc_opsin1 | 0.265 | 0.445 | 0.195 | 0.170 | 0.056 | 0.220 | 0.199 | 0.252 | 0.285 | 13.920 | 24.169 | 12.980 | 0.724 | 0.491 | 0.391 | 1.136 | 1.344 | 1.495 |
| Sc_opsin2 | 0.000 | 0.000 | 0.000 | 0.000 | 0.000 | 0.000 | 0.000 | 0.000 | 0.000 | 0.000 | 0.000 | 0.000 | 0.000 | 0.000 | 0.000 | 0.031 | 0.333 | 0.132 |
| Sc_opsin3 | 0.000 | 0.000 | 0.000 | 0.000 | 0.000 | 0.000 | 0.000 | 0.000 | 0.000 | 0.000 | 0.000 | 0.000 | 0.000 | 0.000 | 0.000 | 0.000 | 0.000 | 0.000 |
| Sc_opsin4 | 0.076 | 0.000 | 0.000 | 0.000 | 0.000 | 0.000 | 0.000 | 0.000 | 0.069 | 0.000 | 0.000 | 0.505 | 0.000 | 0.000 | 0.000 | 0.000 | 0.346 | 0.123 |
| Sc_opsin5 | 0.000 | 0.000 | 0.031 | 0.000 | 0.000 | 0.000 | 0.000 | 0.000 | 0.060 | 0.030 | 0.025 | 0.028 | 0.000 | 0.000 | 0.000 | 0.257 | 0.334 | 0.324 |
| Sc_opsin6 | 0.000 | 0.000 | 0.000 | 0.000 | 0.000 | 0.000 | 0.000 | 0.000 | 0.000 | 0.000 | 0.000 | 0.000 | 0.000 | 0.706 | 0.067 | 0.000 | 0.000 | 0.000 |
| Sc_opsin7 | 1.061 | 0.699 | 1.151 | 1.092 | 2.278 | 0.993 | 3.084 | 1.964 | 2.506 | 16.099 | 10.653 | 18.641 | 1.850 | 3.550 | 4.412 | 8.005 | 7.924 | 13.264 |
| Sc_opsin9 | 0.000 | 0.000 | 0.000 | 0.000 | 0.000 | 0.000 | 0.000 | 0.000 | 0.000 | 0.000 | 0.000 | 0.000 | 0.000 | 0.000 | 0.000 | 0.000 | 0.000 | 0.000 |
| Sc_opsin11 | 0.431 | 0.201 | 0.463 | 1.231 | 4.425 | 3.648 | 0.269 | 0.822 | 0.774 | 0.000 | 0.054 | 0.000 | 0.210 | 1.398 | 1.742 | 0.110 | 0.391 | 0.058 |
| Sc_opsin12 | 0.000 | 0.000 | 0.052 | 0.000 | 0.000 | 0.000 | 0.000 | 0.107 | 0.177 | 0.579 | 0.000 | 0.116 | 0.000 | 0.000 | 0.000 | 1.054 | 0.738 | 0.883 |
| Sc_opsin13 | 0.000 | 0.000 | 0.031 | 0.000 | 0.000 | 0.000 | 0.000 | 0.032 | 0.000 | 0.030 | 0.000 | 0.055 | 0.000 | 0.000 | 3.463 | 0.000 | 0.000 | 0.000 |
| Sc_opsin14 | 0.112 | 0.209 | 0.000 | 0.000 | 0.119 | 0.000 | 0.052 | 0.053 | 0.653 | 0.050 | 0.042 | 0.000 | 0.109 | 0.000 | 0.000 | 0.043 | 0.203 | 0.045 |
| Sc_opsin16 | 0.000 | 0.000 | 0.000 | 0.000 | 0.000 | 0.000 | 0.000 | 0.000 | 0.000 | 0.000 | 0.000 | 0.000 | 0.000 | 0.000 | 0.000 | 0.000 | 0.000 | 0.000 |
| Sc_opsin17 | 0.121 | 0.113 | 0.037 | 0.130 | 0.387 | 0.420 | 0.000 | 0.039 | 0.036 | 0.036 | 0.031 | 0.000 | 0.000 | 0.038 | 0.000 | 0.155 | 0.073 | 0.196 |
| Sc_opsin18 | 0.000 | 0.000 | 0.000 | 0.000 | 0.000 | 0.000 | 0.466 | 0.263 | 0.198 | 0.050 | 0.000 | 0.000 | 0.162 | 0.205 | 0.000 | 0.169 | 0.100 | 0.000 |
| Sc_opsin19 | 0.000 | 0.000 | 0.000 | 0.000 | 0.000 | 0.000 | 0.000 | 0.000 | 0.000 | 0.081 | 0.000 | 0.000 | 0.000 | 0.000 | 0.000 | 0.000 | 0.000 | 0.000 |
| Sc_opsin20 | 0.000 | 0.000 | 0.000 | 0.000 | 0.000 | 0.000 | 0.000 | 0.000 | 0.000 | 0.068 | 0.000 | 0.000 | 0.000 | 0.000 | 0.000 | 0.000 | 0.000 | 0.000 |
| Sc_opsin21 | 0.000 | 0.000 | 0.000 | 0.000 | 0.000 | 0.000 | 0.000 | 0.000 | 0.000 | 0.000 | 0.000 | 0.000 | 0.000 | 0.000 | 0.000 | 0.000 | 0.000 | 0.000 |
| Sc_opsin22 | 0.157 | 0.293 | 0.096 | 0.056 | 0.083 | 0.000 | 0.147 | 0.174 | 0.023 | 0.164 | 0.178 | 0.065 | 0.281 | 0.145 | 0.110 | 0.280 | 0.355 | 0.148 |
| Sc_opsin23 | 0.023 | 0.000 | 0.000 | 0.025 | 0.000 | 0.000 | 0.000 | 0.156 | 0.021 | 0.231 | 0.071 | 0.039 | 0.091 | 0.043 | 0.025 | 0.143 | 0.085 | 0.170 |
| Sc_opsin24 | 0.000 | 0.000 | 0.000 | 0.000 | 0.000 | 0.000 | 0.000 | 0.000 | 0.000 | 0.000 | 0.040 | 0.000 | 0.051 | 0.000 | 0.000 | 0.000 | 0.000 | 0.000 |
| Sc_opsin25 | 0.000 | 0.000 | 0.000 | 0.037 | 0.000 | 0.000 | 0.000 | 0.000 | 0.000 | 0.277 | 0.208 | 0.028 | 0.000 | 0.000 | 0.000 | 0.000 | 0.000 | 0.000 |
| Sc_opsin27 | 0.000 | 0.000 | 0.079 | 0.000 | 0.000 | 0.000 | 0.000 | 0.000 | 0.000 | 0.000 | 0.000 | 0.424 | 1.170 | 0.079 | 0.000 | 0.000 | 0.000 | 0.690 |

**Table S6.** Detailed information of sequences used as queries to identify *S. constricta* opsins and Gα proteins

| Species | Accession number | Sequence name |
| --- | --- | --- |
| *Homo sapien* | NP_000530.1 | rhodopsin |
|  | AAB05207.1 | c-opsin, blue cone pigment |
|  | CAA92342.1 | c-opsin, cone pigments, long-wave-sensitive |
|  | NP_150598.1 | melanopsin |
|  | AAB92384.1 | RPE-retinal G protein coupled receptor |
|  | AAC51757.1 | peropsin |
|  | AAR21109.1 | neuropsin |
|  | AAD32671.1 | encephalopsin |
| *Drosophila melanogaster* | NP_524407.1 | rhodopsin1 |
|  | NP_524398.1 | rhodopsin2 |
|  | NP_524411.1 | rhodopsin3 |
|  | NP_476701.1 | rhodopsin4 |
|  | NP_477096.1 | rhodopsin5 |
|  | NP_524368.5 | rhodopsin6 |
|  | NP_524035.2 | rhodopsin7 |
| *Branchiostoma floridae* | BAE00065.1 | melanopsin |
|  | BAC76023.1 | peropsin |
|  | BAC76019.1 | Go-opsin |
|  | BAC76021.1 | rhodopsin |
| *Mizuhopecten yessoensis* | BAA22218.1 | Go-opsin |
|  | XP_021358232.1 | neuropsin |
| *Argopecten irradians* | ALO02514.1 | Gq-opsin1 |
|  | ALO02515.1 | Gq-opsin2 |
|  | APB88016.1 | Xenopsin |
|  | APB88017.1 | Xenopsin |
|  | APB88020.1 | retinochrome |
| *Euprymna scolopes* | ACB05672.1 | rhodopsin |
|  | ACB05673.1 | rhodopsin |
| *Homo sapien* | NP_002061.1 | Gαi1 |
|  | NP_002064.1 | Gαi2 |
|  | NP_006487.1 | Gαi3 |
| *Homo sapien* | NP_000507.1 | Gαs |
|  | NP_004288.1 | Gαq1 |
|  | NP_002063.2 | Gαq2 |
|  | NP_002058.2 | Gαq3 |
|  | NP_066268.1 | Gαo |
|  | NP_006563.2 | Gα12 |
| *Drosophila melanogaster* | NP_725191.1 | Gαq |
|  | NP_523684.2 | Gαo |
|  | NP_477502.1 | Gαi |
|  | NP_001036421.1 | Gα12 |
|  | NP_477506.1 | Gαs |

**Table S7.** Detailed information of primers used in this study

| Primer | Sequence (5’→3’) | Application |
| --- | --- | --- |
| opsin1-F | ATGAATGAGGATTTTGCTATTAACAAT | ORF amplification |
| opsin1-R | CTACATCGAAGTAGTCATTTTACCAGG |  |
| opsin5-F  opsin5-R | ATGAATGGCACAACCGTTGG |  |
|  | TCACAGCTGATCTTCATCACTTTGT |  |
| opsin7-F  opsin7-R | ATGGGTTCAGATTCAGAGGAACA |  |
|  | CTAGTTATTCTTCTTCTCGTATGTGACC |  |
| opsin12-F | ATGAGTACTTGGTTGATTCGGGA |  |
| opsin12-R | TTAAACCAGTTCCACAGTTTCTAGATG |  |
| q-opsin1-F | ACCACGGCATACGTTACGAGAC | qPCR |
| q-opsin1-R | AGCACACATGGAATACCAGCAC |  |
| q-opsin5-F | TGACGCCATTCTGGTCTGAGT |  |
| q-opsin5-R | GCAACACATCAACCAAGGCAT |  |
| q-opsin7-F | ATGTCGGCACCATCAGCACT |  |
| q-opsin7-R | AACGGTTGTGTTGGCAGACTG |  |
| q-opsin12-F | CTGTTCGCCAAATCTTCAATCA |  |
| q-opsin12-R | CCGAAGGTGATGGAGACGAA |  |
| p-opsin1-F | CCCAAGCTTATGAATGAGGATTTTGCTATTAACAAT | Construction of expression vector |
| p-opsin1-R | TCCCCGCGGCATCGAAGTAGTCATTTTACCAGG |  |
| p-opsin5-F | CCCAAGCTTATGAATGGCACAACCGTTGG |  |
| p-opsin5-R | TCCCCGCGGCAGCTGATCTTCATCACTTTGTAGTT |  |
| p-opsin7-F | CCCAAGCTTATGGGTTCAGATTCAGAGGAACA |  |
| p-opsin7-R | TCCCCGCGGGTTATTCTTCTTCTCGTATGTGACCC |  |
| p-opsin12-F | CCCAAGCTTATGAGTACTTGGTTGATTCGGGA |  |
| p-opsin12-R | TCCCCGCGGAACCAGTTCCACAGTTTCTAGATGG |  |

**Supplementary Figures**

**
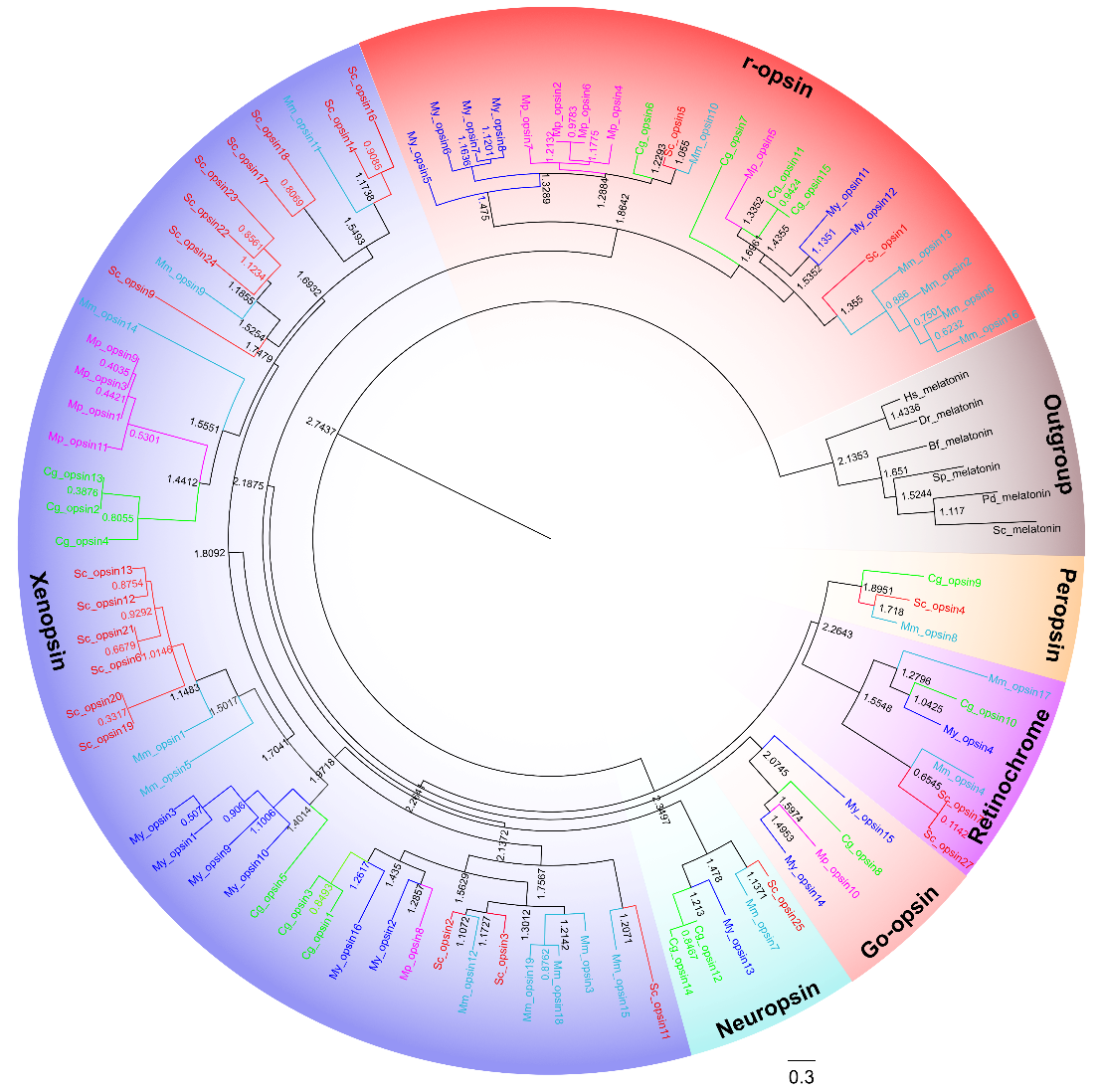
**

**Figure S1. Phylogenetic tree comparing deduced amino acid sequences of putative opsins from five marine bivalves investigated in this study.** The species included four eyeless species (*S. constricta*, *Mercenaria mercenaria*, *Modiolus philippinarum*, and *Crassostrea gigas*), and one species featuring numerous noncephalic eyes (*Mizuhopecten yessoensis*). Their respective putative opsins were highlighted in different colors. Detailed sequences were referred to Tables S2 and S3. The phylogenetic tree was constructed using the maximum-likelihood method via SeaView software (PhyML algorithm) and visualized using Figtree v1.4.3 and Adobe Photoshop CS (version 6.0). Branch lengths of the tree topology were determined by minimizing the sum of squared differences between evolutionary and patristic distances. Different background colors denoted the opsin subfamilies, with the melatonin group selected as an outgroup to root the phylogenetic tree.


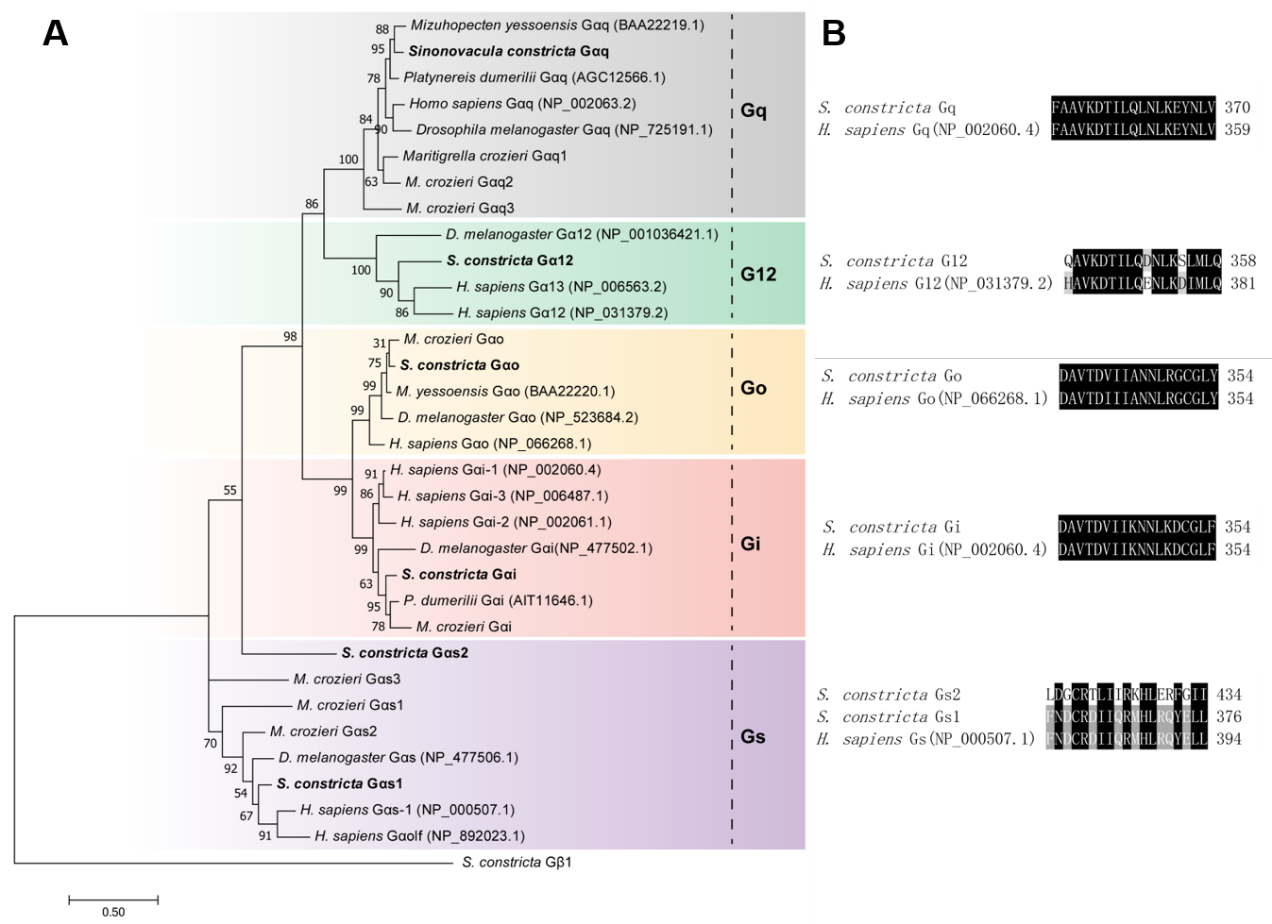


**Figure S2.** **Phylogenetic tree comparing deduced amino acid sequences of putative Gα proteins from *S. constricta* with typical Gα proteins from representative organisms (A), and their C-terminal sequence alignment with those from *H. sapiens* (B).** The phylogenetic tree construction was performed using MEGA 7 software, employing the maximum-likelihood method based on the JTT matrix-based model. Confidence in the resulting branch topology of the phylogenetic tree was assessed through bootstrapping with 1,000 iterations. Sequence alignment was conducted using Clustalx 2.1 software.


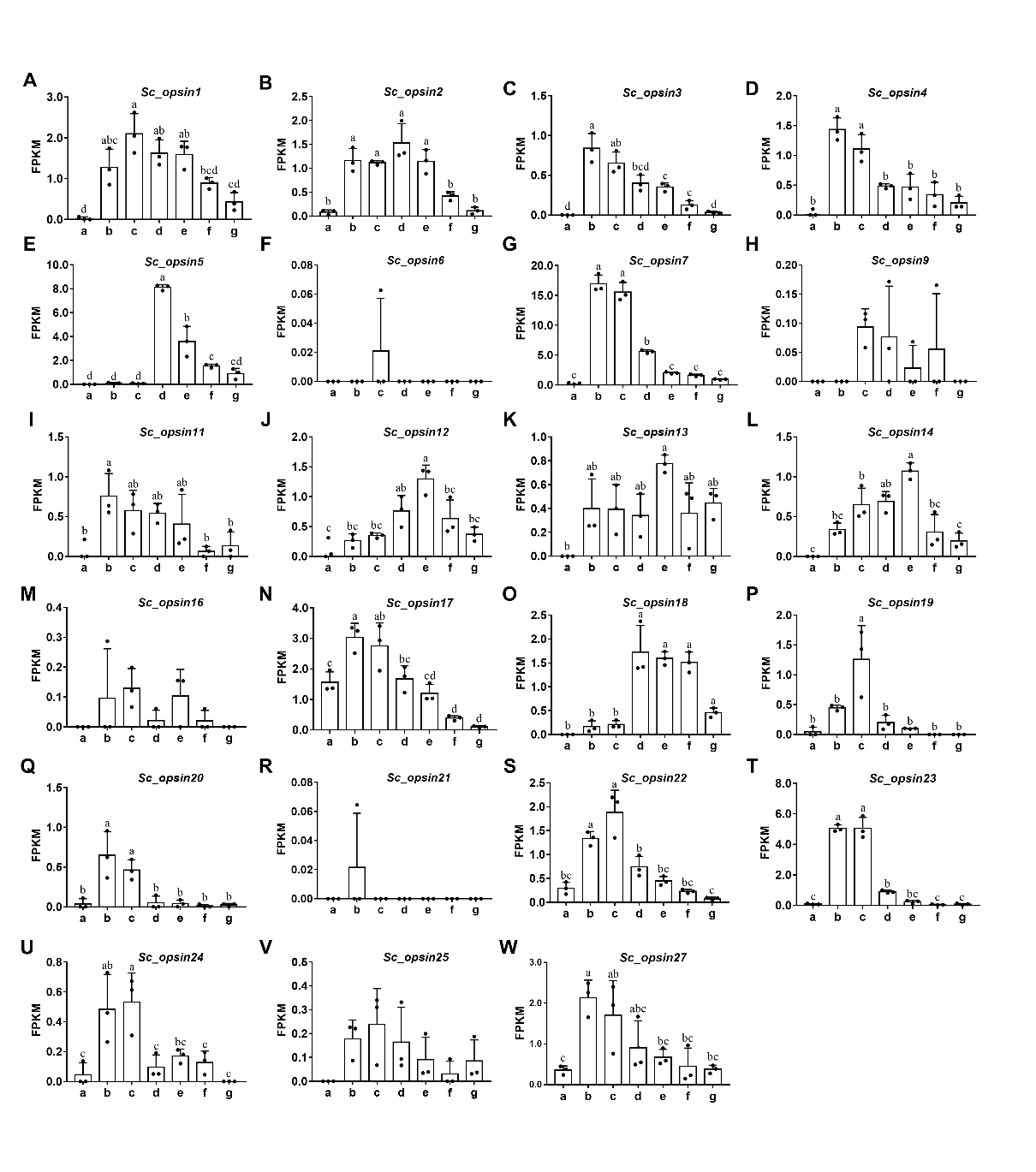


**Figure S3.** **Expression patterns of the same *S. constricta* opsin across various developmental stages (a: zygotes; b: trochophore larvae; c: veliger larvae; d: umbo larvae; e: creeping larvae; f: single pipe larvae; g: juvenile clams).** The values (mean ± SD) were obtained from FPKM values in the transcriptomic data (Table S4). Different letters indicate significant differences at *P* < 0.05. Statistical analysis was performed using SPSS 20 software with one-way ANOVA followed by Tukey’s test.


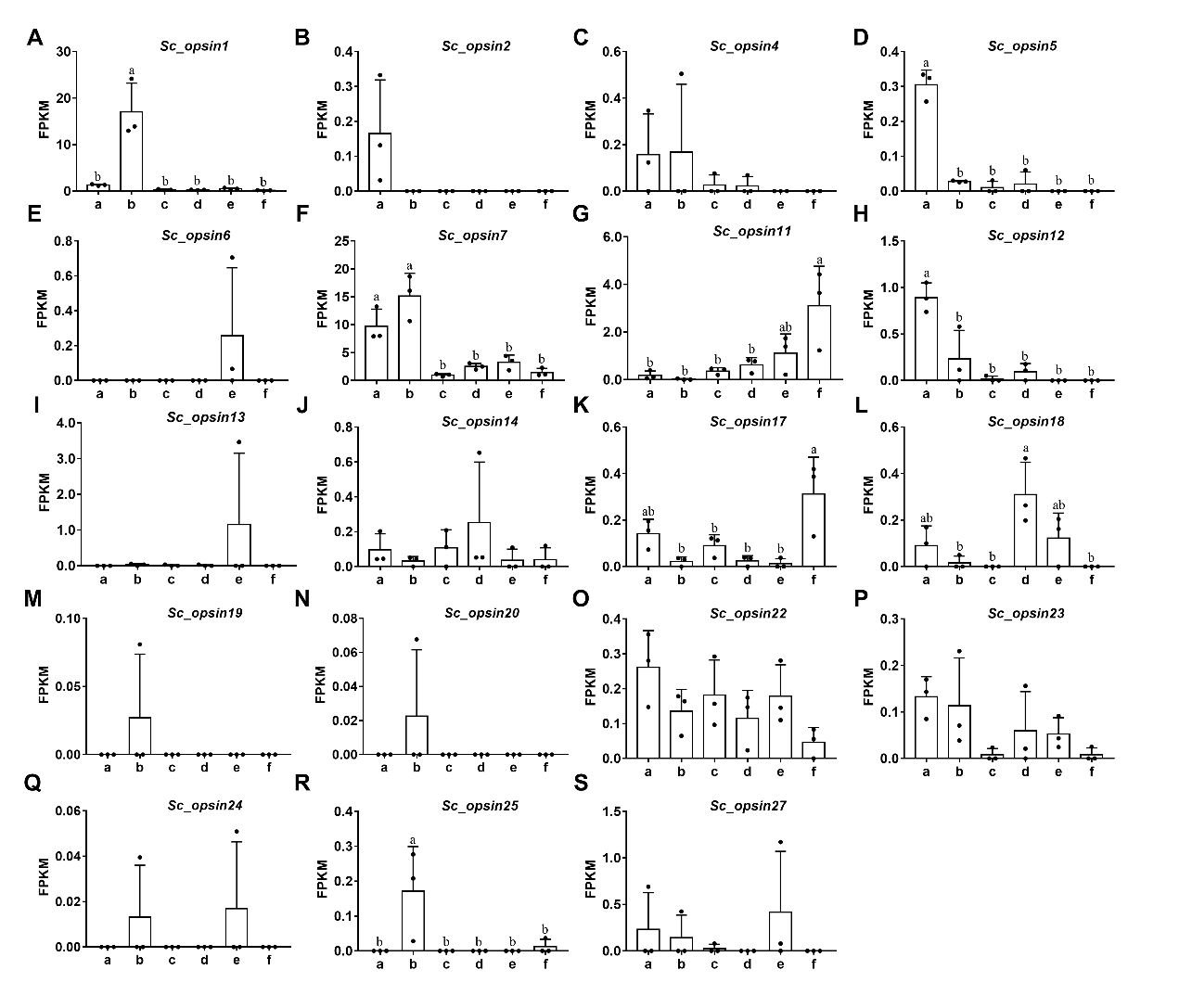


**Figure S4. Expression patterns of the same *S. constricta* opsin across various tissues (a:** **siphon; b:** **foot; c: gill; d: labial palp; e: mantle; f: intestine).** The values (mean ± SD) were obtained from FPKM values in the transcriptomic data (Table S5). Different letters indicate significant differences at *P* < 0.05. Statistical analysis was performed using SPSS 20 software with one-way ANOVA followed by Tukey’s test.


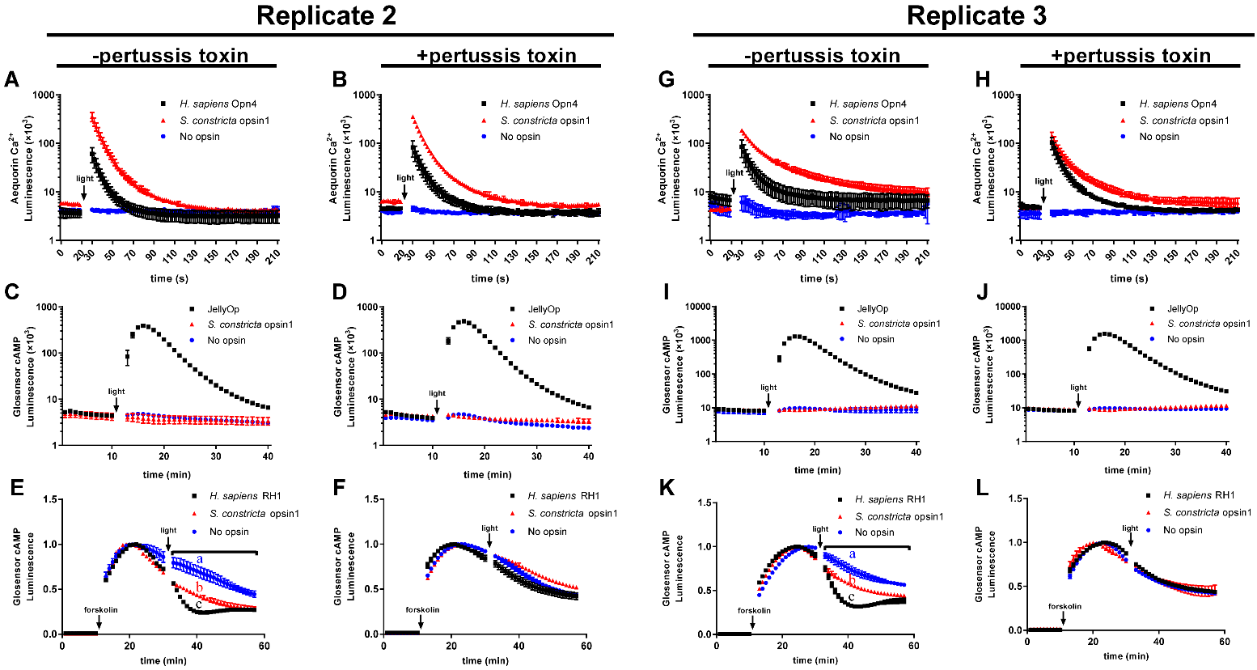


**Figure S5. Two additional biological replicates of the secondary messenger assays of *S. constricta* opsin1.** Cells transfected with the empty vector (no opsin) served as the negative control. Cells transfected with *H. sapiens* Opn4 were used as the positive control to measure the luminescence of Ca^2+^ increase (A, B, G, and H). Cells transfected with JellyOp served as the positive control to detect the luminescence of cAMP increase (C, D, I, and J), while cells transfected with *H. sapiens* RH1 were used as the positive control to detect the luminescence of cAMP decrease (E, F, K, and L). The mean luminescence values (mean ± SD) for each treatment were obtained from one biological experiment with three technical replicates. Following an initial equilibration period, as indicated in the panels, cells were exposed to white light at approximately 20 μmol/m^2^/s for 5 s. Additionally, cells were treated with either (-) (A, C, E, G, I, and K) or (+) (B, D, F, H, J, and L) pertussis toxin, which specifically inhibits the Gαi but not the Gαs or Gαq-coupled signal pathway. To measure the Gαi cascade (E, F, K, and L), cells were pre-treated with forskolin to artificially elevate cAMP levels before light exposure. The statistical analysis for comparing luminescence values among different trials in panel E was conducted using repeated measures ANOVA with SPSS 20 software, and significant differences were denoted by different letters (*P* < 0.05).

**
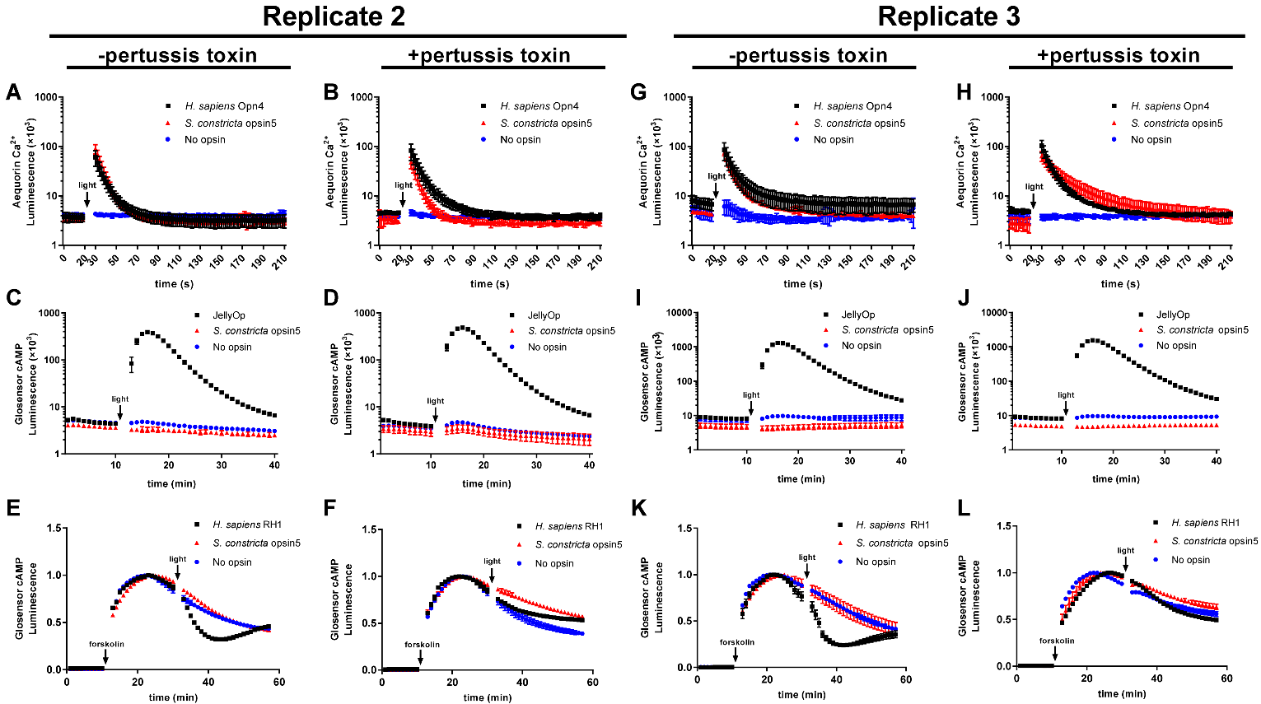
**

**Figure S6. Two additional biological replicates of the secondary messenger assays of *S. constricta* opsin5.** The detailed figure information was identical to that presented in Figure S5. Notably, the values of the negative and positive controls in panels A, B, C, D, G, H, I, and J are duplicated from Figure S5, respectively.


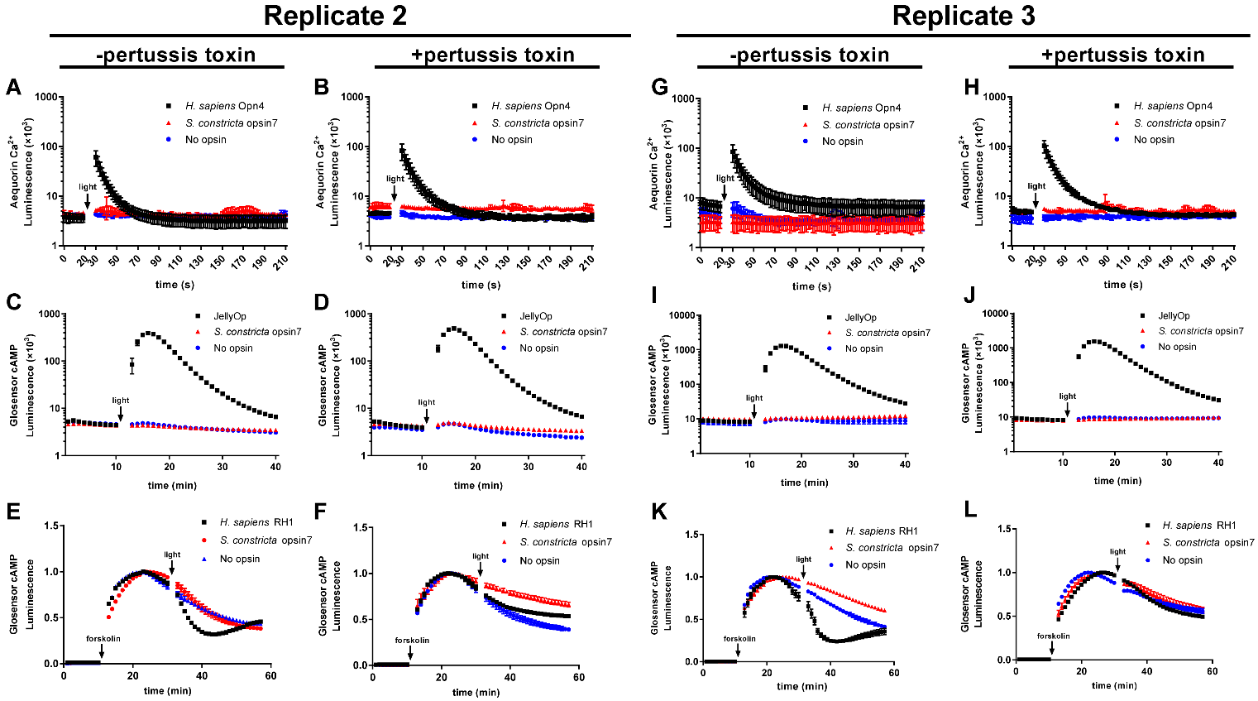


**Figure S7. Two additional biological replicates of the secondary messenger assays of *S. constricta* opsin7.** The detailed figure information was identical to that presented in Figure S5. Notably, the values of all negative and positive controls are duplicated from Figure S6, respectively.

**
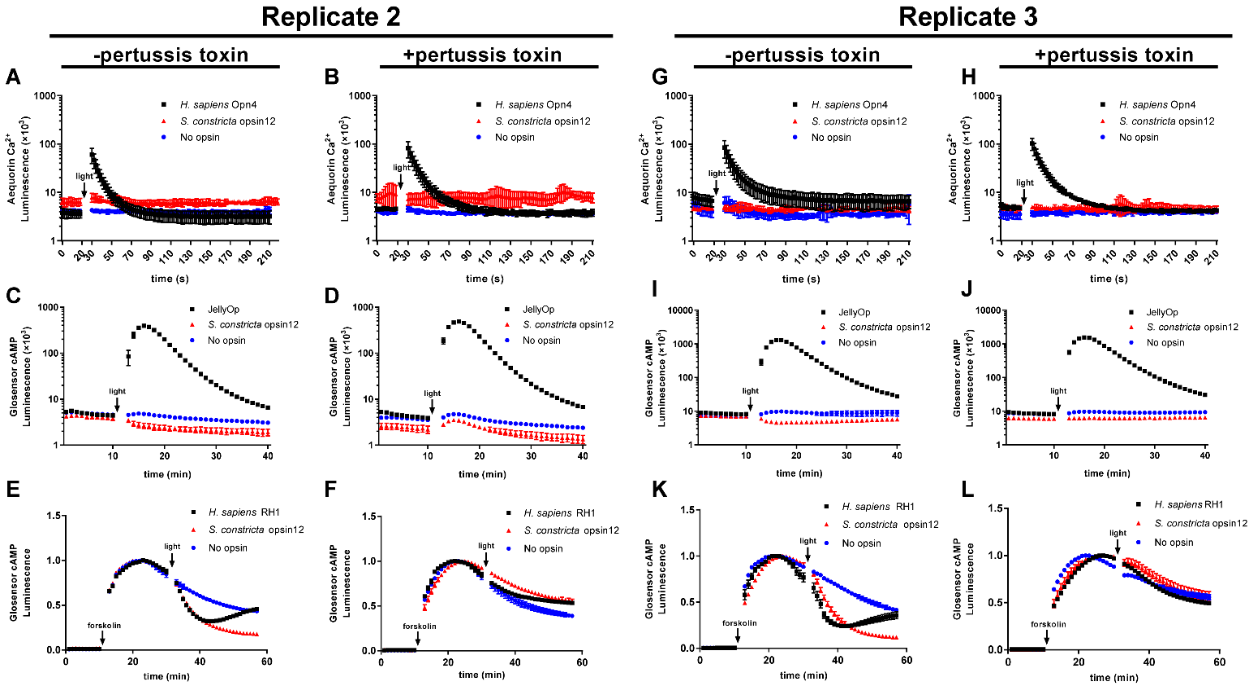
**

**Figure S8. Two additional biological replicates of the secondary messenger assays of *S. constricta* opsin12.** The detailed figure information was identical to that presented in Figure S5. Notably, the values of all negative and positive controls are duplicated from Figure S6, respectively.


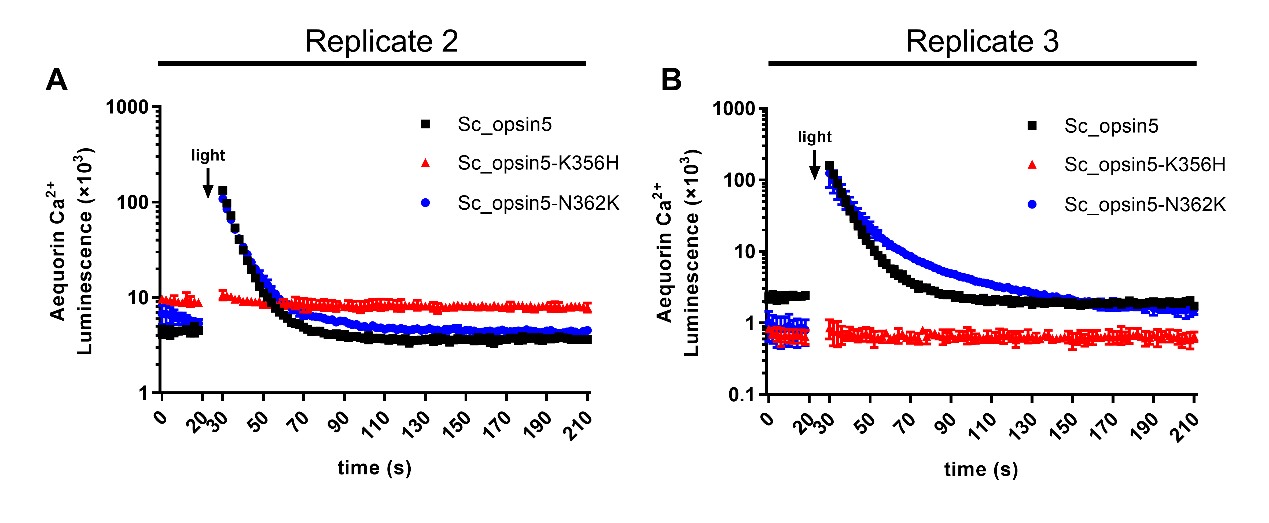


**Figure S9. Two additional biological replicates of the phototransduction sensitivity of *S. constricta* opsin5 mutants determined by measuring luminescence of Ca^2+^ levels upon exposure to light.** Cells transfected with wild-type Sc_opsin5 served as the positive control. After an initial equilibration period, as indicated in the panel, cells were exposed to white light at approximately 20 μmol/m^2^/s for 5 s. The mean luminescence values (mean ± SD) for each treatment were obtained from one biological experiment with three technical replicates.


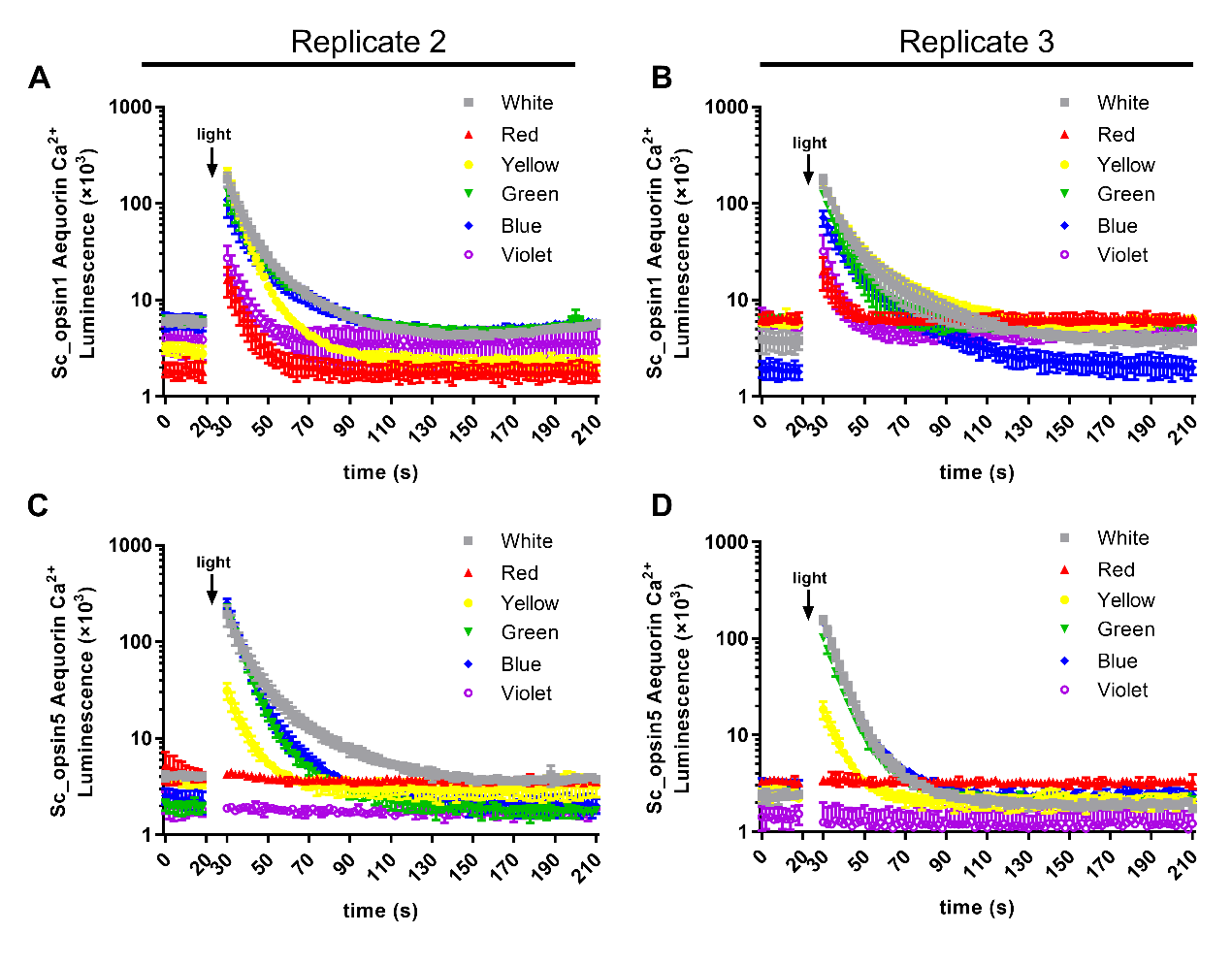


**Figure S10. Two additional biological replicates of phototransduction sensitivity driven by Sc_opsin1 (A and C) and 5 (B and D), as detected by luminescence of Ca^2+^ levels upon exposure to different light spectra.** The light spectra included white, red, yellow, green, blue, and violet. The mean luminescence values (mean ± SD) for each treatment were obtained from one biological experiment with three technical replicates. After an initial equilibration period, as indicated in the panels, cells were exposed to the respective light spectra at approximately 20 μmol/m^2^/s for 5 s.


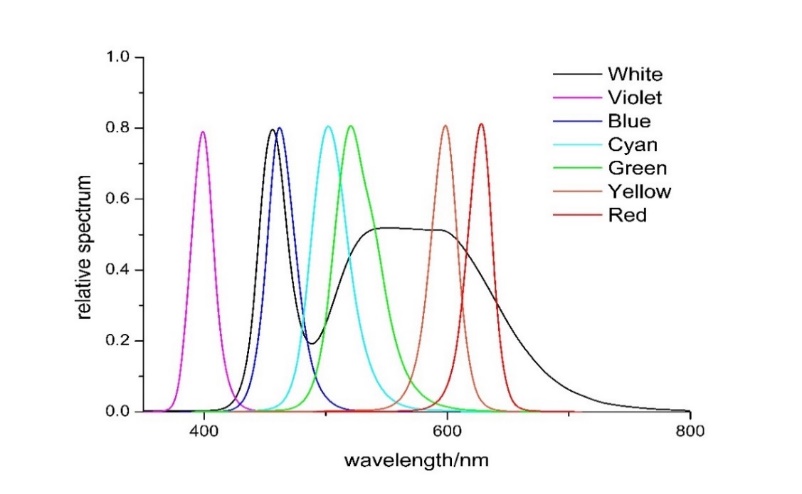


**Figure S11. Characteristics of light spectra used in this study.** The light spectra included white light (with a peak at 400-800 nm), red light (with a peak at 627 nm), yellow light (with a peak at 591 nm), green light (with a peak at 523 nm), cyan light (with a peak at 501 nm), blue light (with a peak at 463 nm), and violet light (with a peak at 397 nm).
